# Inability of *Prevotella bryantii* to Form a Functional Shine-Dalgarno Interaction Reflects Unique Evolution of Ribosome Binding Sites in *Bacteroidetes*

**DOI:** 10.1371/journal.pone.0022914

**Published:** 2011-08-12

**Authors:** Tomaž Accetto, Gorazd Avguštin

**Affiliations:** Animal Science Department, Biotechnical Faculty, University of Ljubljana, Domžale, Slovenia; University of Edinburgh, United Kingdom

## Abstract

The Shine-Dalgarno (SD) sequence is a key element directing the translation to initiate at the authentic start codons and also enabling translation initiation to proceed in 5′ untranslated mRNA regions (5′-UTRs) containing moderately strong secondary structures. Bioinformatic analysis of almost forty genomes from the major bacterial phylum *Bacteroidetes* revealed, however, a general absence of SD sequence, drop in GC content and consequently reduced tendency to form secondary structures in 5′-UTRs. The experiments using the *Prevotella bryantii* TC1-1 expression system were in agreement with these findings: neither addition nor omission of SD sequence in the unstructured 5′-UTR affected the level of the reporter protein, non-specific nuclease NucB. Further, NucB level in *P. bryantii* TC1-1, contrary to hMGFP level in *Escherichia coli*, was five times lower when SD sequence formed part of the secondary structure with a folding energy -5,2 kcal/mol. Also, the extended SD sequences did not affect protein levels as in *E. coli*. It seems therefore that a functional SD interaction does not take place during the translation initiation in *P. bryanttii* TC1-1 and possibly other members of phylum *Bacteroidetes* although the anti SD sequence is present in 16S rRNA genes of their genomes. We thus propose that in the absence of the SD sequence interaction, the selection of genuine start codons in *Bacteroidetes* is accomplished by binding of ribosomal protein S1 to unstructured 5′-UTR as opposed to coding region which is inaccessible due to mRNA secondary structure. Additionally, we found that sequence logos of region preceding the start codons may be used as taxonomical markers. Depending on whether complete sequence logo or only part of it, such as information content and base proportion at specific positions, is used, bacterial genera or families and in some cases even bacterial phyla can be distinguished.

## Introduction

Shine-Dalgarno (SD) sequence of the prokaryotic mRNA is commonly regarded as a key element involved in the selection of the authentic start codons versus internal AUG codons. It is usually 4–5 bp long, positioned 5–8 bp upstream from the start codon, and basepairs with the complementary sequence (anti SD) at the 3′ end of 16S rRNA of the 30S ribosomal subunit. This interaction also makes possible the translation initiation in the presence of mRNA secondary structures weaker than −6 kcal/mol, while translation initiation rapidly becomes inefficient in the presence of more stable secondary structures in *Escherichia coli*. [1]–[3]. The SD sequence is found in majority of translation initiation regions of *Escherichia coli* [4]. It is thought that 4–5 bp Shine-Dalgarno sequence-16S rRNA interaction is usually sufficient since SD sequence lengthening only rarely results in increased translation efficiency and longer, 8 or 10 bp interaction actually inhibits translation [1], [5], [6]. It was shown, on the other hand, that *E. coli* can efficiently initiate translation also in A/U rich translation initiation sites lacking SD sequence altogether [7] and that translation initiation of leaderless mRNA proceeds without mRNA-16S rRNA interaction [8]. It has recently been observed, as the number of sequenced bacterial genomes increased, that a significant share of genes is not preceded by SD sequence [9]. Further, the share of genes preceded by SD sequence was found to be phylum specific. It was suggested that phyla exhibiting low fractions of genes preceded by SD sequence in their member's genomes rely primarily on the ability of ribosomal protein S1 to mediate translation initiation [10]. The ribosomal protein S1 binds to the A/U rich stretch of mRNA upstream of the start codon and is not universally conserved in bacteria: in *Firmicutes* which possess the largest fraction of genes preceded by SD sequence, the protein S1 is predicted to be non-functional in translation initiation [11].

Ribosome binding sites of an organism may be conveniently represented by a sequence logo. Sequence logo primarily describes the information content of individual sites in the sequence alignment. The information content is a measure of sequence conservation at the specific site and is represented by the height of the logo at that position. Relative frequency of bases, for a nucleic acid logo, is described by the height of individual stacked symbols specific for given bases at that site. Sequence logo thus combines different information in one picture and is usually used to display protein binding sites of nucleic acids, and protein motifs [12].

*Prevotella bryantii* is a strictly anaerobic gram negative bacterium involved in the degradation of plant cell wall polysaccharides in the rumen of cattle and sheep [13]. It belongs to the large bacterial phylum *Bacteroidetes* whose representatives inhabit diverse habitats such as fresh and salt water, sediments and animal gastrointestinal tract [14]. The *Bacteroidetes* are known for their somewhat unique genetic make up. The consensus promoter sequences and their spacing are for example distinct from those known in *E. coli* [15], [16], which is thought to be the consequence of the unusual primary sigma factor [17]. The development of general gene manipulation techniques in *Bacteroidetes* was therefore rather slow, with the exception of the genus *Bacteroides* [18] to a certain extent. Recently we developed a gene expression system for *Prevotella bryantii* TC1-1 strain based on a shuttle plasmid pRH3. The expression was regulated by transcriptional fusion of the expressed gene with *tetQ*. Expression was not seen in the absence of tetracycline when *nucB*, a gene coding for a non-specific nuclease, and 108 bp of its upstream sequence were cloned into pRH3 [19]. The abovementioned studies on translation initiation diversity in bacteria by Chang et al. [9] and Nakagawa et al. [10] touched the *Bacteroidetes* only briefly by analyzing genomes of four species in total. These studies found that the genomes from *Bacteroidetes* genera *Bacteroides*, *Porphyromonas* and *Cytophaga* contain a low fraction of genes preceded by SD sequence. The translation initiation in *Bacteroidetes* was predicted to rely on ribosomal protein S1 which should be functional according to sequence analysis [11]. In order to comprehensively analyze the start codon upstream regions in *Bacteroidetes* we examined almost forty *Bacteroidetes* genomes which span the wide diversity of this phylum. The *Prevotella bryantii* TC1-1 expression system was used to examine the effects of sequence manipulation involving the start codon upstream region on NucB protein level. Bioinformatic analysis confirmed that the low proportion of genes preceded by SD sequence is truly a phylum wide trait in *Bacteroidetes* and also presented the way the translation initiation region evolved in *Bacteroidetes* presumably to serve in ribosomal protein S1 mediated initiation. Experimental evidence suggests that the ability to form a functional SD-anti SD interaction during translation initiation was lost in *Bacteroidetes* evolution explaining lack of SD sequence in the majority of genes.

## Results

### Sequence logos detect SD sequence in the genomes of many prokaryote phyla

**Figure 1:**
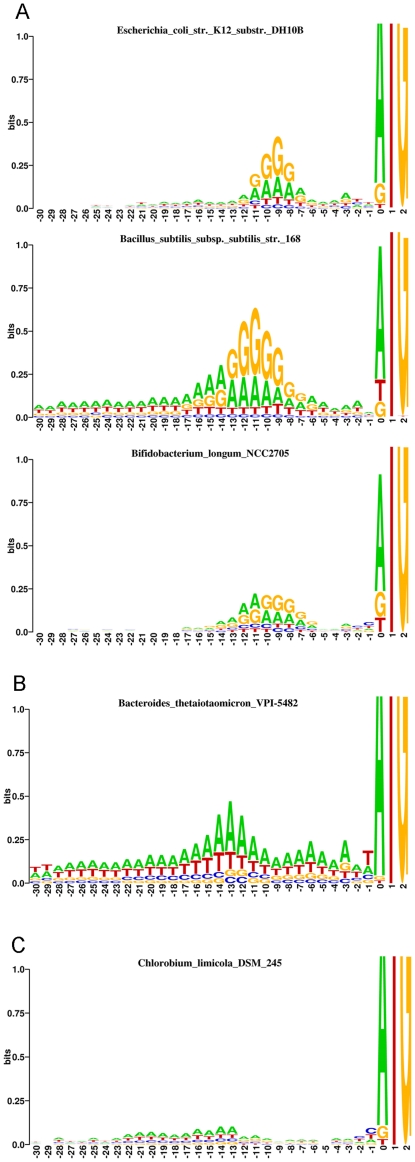
Sequence logo analysis of start codon upstream regions. A: Sequence logos showing the presence of SD sequence in some well known bacteria. Sequence logos of *Bacteroidetes* (B) and *Chlorobi* (C) demonstrate the lack of SD sequence. The position 0 represents the nucleotide position of start codon.

The genome wide presence of SD sequence leads to the enrichment of adenine and guanine bases in sequence logo spanning the −5 to −10 bp region relative to the start codon of a gene [4], [12]. Such enrichment can be clearly found in sequence logos of major bacterial phyla: *Proteobacteria*, *Firmicutes*, *Actinobacteria*, *Thermotogae*, *Chloroflexi*, and *Aquificae* (Fig. 1 A and S2, S6, S9, S10, S12, S14). Notable exception in *Proteobacteria* are the low GC % parasites or symbionts from the family of *Rickettsiales* and the *Tenericutes* from the genus *Mycoplasma*, which possess only slight enrichment in guanine in SD sequence region (Fig. S10 and S15). The sequence logos of species containing the SD sequence and belonging to the same phylum are not uniform, however. The heterogeneity sometimes reflects the differences in GC content of the species in the absence of drastic logo changes in SD sequence area e.g. *Moorella thermoacetica* with 55,8% GC and *Clostridium acetobutylicum* with 30,9% GC genomic content (Fig. S9), but in other cases the overall shape of a logo in SD sequence area is changed e.g. between *Bifidobacterium longum* and *Arthrobacter aurescens* (Fig. S14). The SD sequence is less obvious but still detectable in sequence logos of certain phyla e.g. *Spirochetes*, *Acidobacteria*, *Chlamydiae*, *Deinococcus*-*Thermus*, *Fibrobacteres* and some *Cyanobacteria* (Fig. S1, S3, S7, S8, S11, S13).

### The genomes from the phyla *Bacteroidetes* and *Chlorobi* lack detectable SD sequence

When the regions preceding the start codons in almost forty available genomes from the phylum *Bacteroidetes,* spanning the diversity of this phylum, are examined, no similarity to above mentioned logos of other major phyla is apparent. Instead, the major feature of *Bacteroidetes* logo is an adenine and thymine enrichment centered at 12–13 bases ahead of the start codon (Fig. 1 B, S5). *Chlorobi*, however, lack the information almost totally i.e. they have random base composition in regions preceding the start codons. The only recognizable features shared by most *Chlorobi* species is a slight adenine and thymine enrichment in the region spanning 23–11 bases ahead of the start codon and an enrichment in cytosine and thymine directly before the start codon (Fig. 1 C, S4). The exceptions in *Bacteroidetes* are *Rhodothermus marinus* DSM 4252 and *Salinibacter ruber* DSM 13855 whose logos are aberrant (Fig. S5), the logo of the former more resembling those of *Chlorobi*.

### Sequence logos of regions preceding the start codons *in Bacteroidetes* are conserved at the genus-family level

The logos of all of the eight sequenced species belonging to genus *Bacteroides* examined are characterized in addition to abovementioned AT enrichment centered at −13 bp relative to start codon (Fig. 1 B) also by (i) a similar yet smaller enrichment spanning the −8 to −6 region, (ii) A enrichment at −3 position and (iii) T enrichment at -1 (Fig. 1 B, S5). This type of logo is conserved also in all four strains of genus *Prevotella*, all three strains of genus *Parabacteroides*, strain of *Alistipes,* and all four strains of genus *Porphyromonas* examined, the latter containing very low information content and resembling *Chlorobi* in this respect. In three strains from the genus *Porphyromonas* a shift of the AT enrichment center from −13 to −14 (Fig. S5) can be seen. The essential features of the logo excluding its height are thus conserved also at the level of order *Bacteroidales* encompassing *Prevotellaceae*, *Bacteroidaceae*, *Porphyromonadaceae* and *Rikenellaceae*. The shape of a logo is, however, not completely conserved e.g. the enrichment centered at −13 bp is not so pronounced in *Prevotella* as in *Bacteroides* or is moved as mentioned above. The variation in logo height in genera *Bacteroides, Prevotella*, *Porphyromonas* and *Parabacteroides* can be observed too. The species with lower % GC in the 30 bp preceding the start codons predictably produce higher logos and vice versa (Fig. S5). The *Flavobacteriaceae* logos differ from those in *Bacteroidales* in the placement of the main AT enrichment which is centered at −12, except for the *Capnocytophaga ochracea* DSM 7271, and by the shape of the −8 enrichment (Fig. S5). *Cytophagaceae* are distinguished by a flat logo, with the enrichment at -13 sometimes barely detectable.

### Translation initiation regions of the *Bacteroidetes* and *Chlorobi* genomes have low GC ratio in comparison to coding regions

Generally, the species from the *Bacteroidetes* phylum have genome GC content below 50%. When the 30 bp regions preceding the start codons are examined the GC content decreases by 12,6±2,3% relative to genome GC content (Table S1). This decrease can be observed also in other phyla but is typically around 5% relative to genome GC content with the exception of the *Chlorobi* with 9,9±1,3% GC decrease (Table S1). The % GC decrease in regions preceding the start codons is conserved also in those rare species from the phylum *Bacteroidetes* which are characterized by somewhat higher GC genome content. In *Robiginitalea biformata* for example, which has a 55% GC genome content and belongs to the family *Flavobacteriaceae* with the predominantly 32–39% GC genome content, a 13% GC decrease can be seen. It is similar in *Dyadobacter fermentans* and *Spirosoma linguale*, members of the family *Cytophagaceae* (Table S1). Again, *Rhodothermus marinus* and *Salinibacter ruber* are the exceptions among *Bacteroidetes* with merely 8,5 and 5,4% GC drop in regions preceding the start codons, respectively.

### 16S rRNA genes of all the analyzed *Bacteroidetes* and *Chlorobi* genomes contain anti SD sequence

All of the 16S rRNA coding *Bacteroidetes* genes contain the anti SD sequence 5′ACCUCCUU 3′ at the expected position at the 3′ end of the gene (Fig. S16) with the exception of *Rhodothermus marinus* and *Salinibacter ruber*, where the last U in the anti SD sequence is replaced by A. It is also apparent from the alignment that *Chlorobi* and *Bacteroidetes* 16S rRNA genes, excluding those of *Rhodothermus marinus* and *Salinibacter ruber*, contain a deletion of approximately 9 nucleotides in comparison to representative 16S rRNA sequences of other phyla. The deletion is located 80 bp upstream of anti SD sequence in the helix 44 of the 16S rRNA [20].

### The SD sequence does not significantly influence NucB protein level in *Prevotella bryantii* TC1-1 strain

Since bioinformatic analysis demonstrated a general lack of SD sequence in *Bacteroidetes*, a question arises whether the SD sequence in the 5′-UTR still has any effect on the translation initiation in these bacteria. To resolve this we used a recently developed gene expression system for *Prevotella bryantii* TC1-1 strain [19] which is one of only four systems for gene delivery and expression in *Bacteroidetes* to our knowledge.

**Figure 2:**
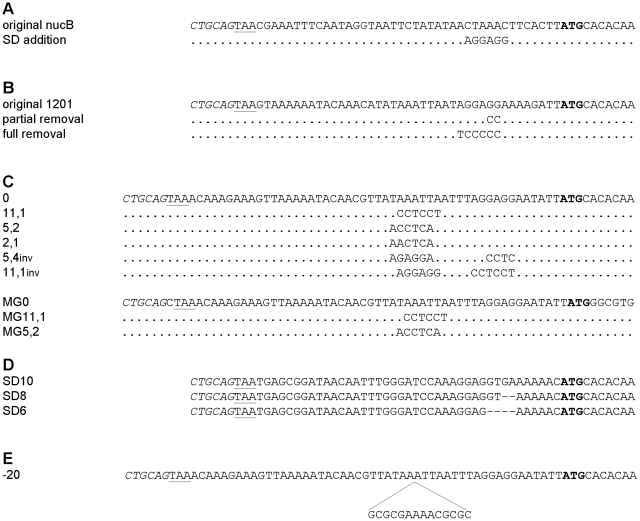
Start codon upstream sequences of plasmid borne reporter genes. The sequence starts with the *Pst*I site of the pRH3 or pUC19 vector (shown in italics). The stop codon at the start of the upstream sequence is underlined and the start codon of *nucB* or *hMGFP* is in boldface. A and B: *nucB* constructs with added (A) or removed (B) SD sequences. C: start codon upstream sequences containing SD sequences that are involved in formation of mRNA secondary structures with different stability. The sequences starting with mg were used in *E. coli* to translate the MGFP. D: Start codon upstream sequences of plasmid pRH3 borne *nucB* constructs containing SD sequence length of 10, 8, and 6 nucleotides. E: *nucB* upstream sequence used to asses the effect of secondary structure 20 bp upstream of the start codon. The secondary structure element was inserted into construct 0 from C after the designated adenosine.

The effect of SD sequence addition or omission on NucB level was tested using pRH3 constructs containing the cognate *nucB* start codon upstream sequence to which the SD sequence was added, or the upstream sequence of PINA_1201, a gene of *Prevotella intermedia* 17, which originally contains the SD sequence, but from which the SD sequence was omitted partially or fully (Fig. 2 A and B). The results are presented in tables 1 and 2. Although there may be minor reductions in the NucB protein levels, both when SD sequence was added or omitted, the NucB levels stayed within the experimental error in strains harboring plasmid constructs with omitted or added SD sequence.

**Table 1:** NucB protein level in *P. bryantii* TC1-1 cultures harboring pRH3 constructs containing added SD sequence relative to the wild type upstream sequences.

plasmid construct name/condition of growth	relative NucB amount	*nucB* mRNA amount
wild type *nucB*	1	1
SD sequence added to *nucB* upstream sequence	0,58±0,28	1,4±0,34
wild type *nucB* in the absence of tetracycline	not done	0,23±0,06

The *nucB* mRNA amount is relative to the amount expressed from the wild type *nucB* plasmid construct.

**Table 2:** NucB protein level in *P. bryantii* TC1-1 cultures harboring pRH3 constructs containing omitted SD sequence relative to the wild type upstream sequences.

plasmid construct name	relative NucB amount	*nucB* mRNA amount
wild type PINA_1201	1	1,14±0,36
SD sequence partially omitted in PINA_1201	0,73±0,36	1,32±0,3
SD sequence fully omitted in PINA_1201	0,45±0,37	0,78±0,17

The *nucB* mRNA amount is relative to the amount expressed from the wild type *nucB* plasmid construct.

### mRNA Secondary structure in the SD sequence region interferes with translation to a different degree in *Prevotella bryantii* TC1-1 and *Escherichia coli*

On the basis of PINA_1201 upstream sequence, further upstream sequences in which SD sequence was part of a secondary structure of different stability were designed (Fig. 2 C). The upstream sequences and pRH3 plasmid constructs were named according to the stability of secondary structure as computed by mFold [21], thus the upstream sequence forming the secondary structure of stability ΔG =  −11,1 kcal/mol was named 11,1 and so forth (Fig. S17). The NucB protein levels in *P. bryantii* TC1-1 strains harboring these constructs as determined by western blot can be seen in table 3. The essentially same upstream sequences were used to initiate translation of *hMGFP* mRNA in *Escherichia coli* TOP10 where upstream sequences and plasmid constructs were named as above and adding the mg prefix. The relative amount of MGFP was determined by western blot and fluorescence. In contrast to *P. bryantii* TC1-1, the upstream variant, which forms a secondary structure containing SD sequence with the stability of -5,2 kcal/mol at 37°C, produced roughly the same amount of protein and fluorescence as the variant forming no secondary structure (table 4). We further designed upstream sequence variants based on 11,1 and 5,2 sequences in which the SD element and its complementary sequence were swapped and named them 11,1inv and 5,4inv (Fig. 2 C, S17). We anticipated that by moving SD sequence further upstream, any extant SD-anti SD interaction would be impaired even more. The amounts of NucB produced from these constructs in *P. bryantii* TC1-1 were comparable to those obtained with constructs 11,1 and 5,2 (table 3) thus strengthening the validity of the former experiments. Minor changes within the experimental error are possible yet while the analysis of construct 11,1inv suggests a rise in NucB protein levels it is the opposite in the case of construct 5,4inv.

**Table 3:** Relative NucB protein level in *P. bryantii* TC1-1 cultures harboring pRH3 constructs containing start codon upstream sequences in which SD sequences form part of a secondary structures with different stabilities.

plasmid construct name	relative amount/western blot	*nucB* mRNA amount
0	1	1,16±0,25
2,1	1,08±0,19	1,60±0,24
5,2	0,12±0,05	0,72±0,05
11,1	0,007±0,0027	0,997±0,13
11,1inv	0,015±0,06	0,63±0,16
5,4inv	0,04±0,02	0,71±0,03

The *nucB* mRNA amount is relative to the amount expressed from the wild type *nucB* plasmid construct. Protein level measurements using western blot were performed at least three times except in the case of constructs 5,2 and 11,1 for which six and five measurements respectively were made.

**Table 4:** Relative hMGFP protein level in *E. coli* TOP10 harboring pUC19 constructs containing start codon upstream sequences in which SD sequences form part of a secondary structures with different stabilities.

plasmid construct name	relative amount/western blot	relative fluorescence	*hMGFP* mRNA amount
mg0	1	1	1
mg5,2	1,21±0,20	1,24±0,19	1,29±0,25
mg11,1	not detected	not detected	not done

### Strong secondary structure 20 bp upstream from start codon does not inhibit translation in *P. bryantii* TC1-1

A hairpin loop was inserted at -21 bp relative to start codon in the upstream sequence 0 (Fig 2 E). The stability of the secondary structure in resulting upstream sequence -20 was computed by mFold to be −9 kcal/mol (Fig. S17). The amount of NucB in *P. bryantii* TC1-1 cultures harboring plasmid construct -20 relative to the plasmid construct 0 was 1,34±0,06. The *nucB* mRNA level was not different in construct −20 relative to the amount in wild type *nucB*: 1,12±0,11.

### Long SD sequence does not affect NucB protein amount in *Prevotella bryantii* TC1-1

The pRH3 based plasmid constructs SD6, SD8 and SD10 containing *nucB* and the identical upstream regions as used in study by Komarova *et al.* [5] in *Escherichia coli* (Fig.2 D) were produced and the NucB protein level was quantified. No correlation between SD sequence length and NucB protein levels was observed (table 5). Typical blot from which the quantifications were made is presented in figure S18.

**Table 5:** Relative NucB protein level in *P. bryantii* TC1-1 cultures harboring pRH3 constructs containing start codon upstream sequences with SD sequences of different length.

plasmid construct name	relative NucB amount	*nucB* mRNA amount
SD10	1	0,77±0,19
SD8	1,26±0,31	0,71±0,1
SD6	0,34±0,12	0,97±0,12

The *nucB* mRNA amount is relative to the amount expressed from the wild type *nucB* plasmid construct.

### Comparison of the NucB protein levels in constructs containing wild-type *nucB*, PINA_1201 or SD10 upstream sequences in *Prevotella bryantii* TC1-1

NucB level was highest in cultures containing PINA_1201 plasmid construct. The NucB protein level in cultures containing *nucB* and SD10 was lower: 0,4±0,17 and 0,13±0,09 respectively, relative to PINA_1201. The mFold mRNA secondary structure prediction in the upstream sequence region revealed lack of secondary structure in PINA_1201 derived mRNA whereas the mRNA from *nucB* and SD series of constructs form a hairpin loop starting at positions preceding the start codon -18 and -14, -16, -18 bp, respectively, (Fig. S19).

### The folding energies of start codon and intra gene methionine codon upstream regions in *Prevotella bryantii* B_1_4 genome

**Figure 3:**
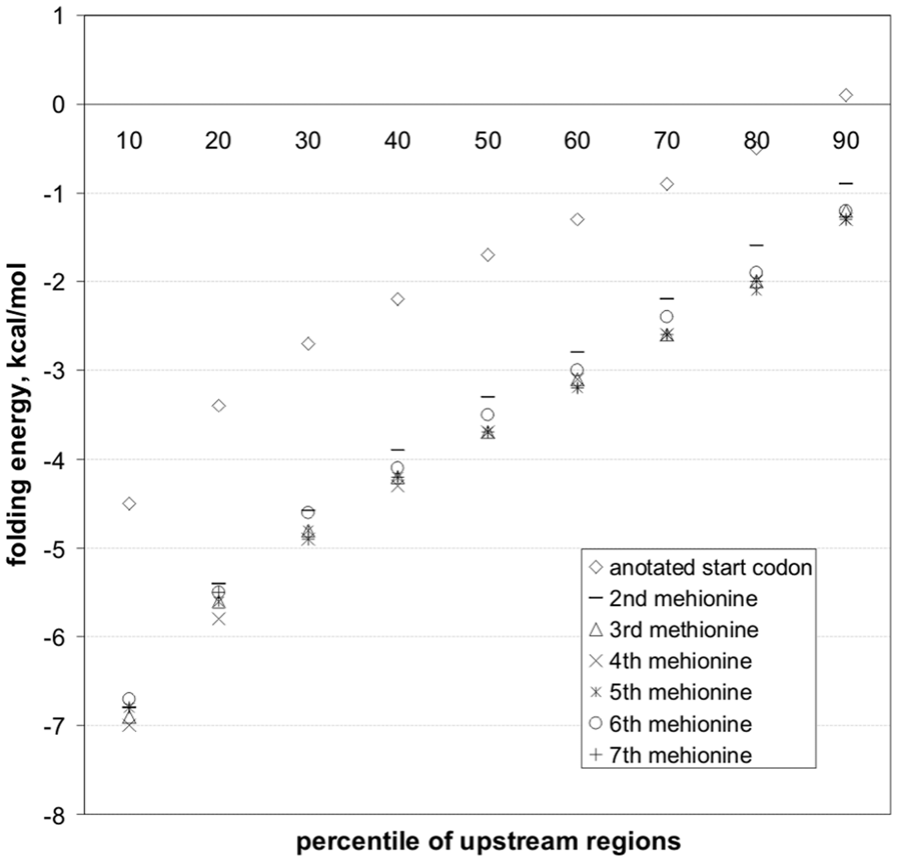
Folding energy distribution of start codon upstream regions in *P. bryantii* B_1_4 genome compared to distributions of successive methionine upstream regions.

In *P. bryantii* B_1_4, the distribution of folding energies of start codon upstream regions differs markedly from those of successive methionine codon upstream regions, the considerable share of the latter being more prone to fold a stable mRNA secondary structure (Fig 3). The same result was obtained using other *Prevotella* genomes. The difference is much smaller in *E. coli* K12 and *Bacillus subtilis* subsp. *subtilis* strain 168 (Table S2).

## Discussion

Any bioinformatic analysis, including the sequence logo of the ribosome binding site region, critically depends on accurately annotated coding sequence start sites. Since the most of the available genome data from various members of the phylum *Bacteroidetes* is currently incomplete and annotation was done generally without human intervention, we focused initially on the complete published genomes of the bacteria from the genus *Bacteroides* only [22]–[25]. In these genomes, the start site annotation was done or verified manually except for the genomes of *B. vulgatus* and *B. distasonis*, now often referred to as *Parabacteroides distasonis* [26], where an additional step using a self training program MED-Start [27] was introduced to refine the start site placement. It was expected that manual annotation favored start sites preceded by canonical SD sequence which was also stressed by Kuwahara *et al.* [23] in *B. fragilis* YCH46 annotation. This did not affect the sequence logos much since they do not reveal canonical SD sequence and do not differ from the *B. vulgatus* and *B. distasonis* logos. Subsequently, we additionally examined almost forty mostly unfinished *Bacteroidetes* genomes and discovered logos similar to ones obtained from genus *Bacteroides*, as described. The only two species lacking the conserved *Bacteroidetes* logo but still not exhibiting the SD sequence, *Rhodothermus marinus* and *Salinibacter ruber*, belong to the *Bacteroidetes* family *Rhodotermaceae* which is only distantly related to other *Bacteroidetes* [14]. Given its AT richness, the *Bacteroidetes* logo superficially resembles logos of parasites from the proteobacterial order *Rickettsiales* or from *Mycoplasma*. Yet there is no −13 AT enrichment in the latter two groups. Further, strains from species with low % GC e.g. *Clostridium acetobutylicum* ATCC 824 and *Streptococcus pneumoniae* D39 with 28,7 and 33,4% GC in the regions preceding the start codon, respectively, resembling the *Bacteroidetes* in this respect, have logos exhibiting typical SD sequences (Fig. S9).

The *Bacteroidetes* logo with the lack of SD sequence and -13 AT enrichment thus represents a true phylum wide characteristic which may reflect the basic features of translation initiation as does the SD sequence in other phyla. Also, the lack of SD sequence in *Bacteroidetes* sequence logos is in agreement with findings of Nagakawa et al. [10] who used the calculation of free energy change resulting from basepairing between anti SD sequence and 5′ untranslated regions of mRNA to identify genes preceded by SD sequence. The logo approach not only detects the SD sequence, but also informs of the translation initiation site structure at the same time. The other prominent characteristic of the *Bacteroidetes* regions preceding the start codons beside the sequence logo is that they are GC poor relative to the whole genome, which is a unique trait shared only by *Chlorobi* in analyzed bacterial phyla (Table S1). Interestingly, as described in results, the 12,6±2,3% GC drop is conserved universally in *Bacteroidetes* species whether their % GC genome content is high or not, e.g. *Alistipes putredinis* with 53% GC and *Flavobacterium psychrophilum* with 32% GC (Table S1). This suggests that the % GC drop itself, not the absolute % GC, is important. This fits the extended Unique accessibility hypothesis of translation initiation [28] well. The hypothesis claims that the decisive factor in authentic start codon selection is not the SD sequence but rather the masking of gene internal methionine codons in mRNA by secondary structure. In *Bacteroidetes* therefore, the local drop in % GC in regions preceding the start codons may result in a stretch of mRNA lacking or forming less stable secondary structures than the rest of the mRNA thereby exposing the authentic start codon regions. The start codon is then chosen according to Nakamoto [28] by multiple interactions with mRNA in the ribosome binding site including SD sequence if extant. *Bacteroidetes,* exhibiting the −13 AT enrichment, which is a possible target for mRNA binding ribosomal protein S1 [5], lack the SD sequence, and it appears that they don't make use of the SD sequence in this step. This is in agreement with our experiments in *P. bryantii* TC1-1 where the addition or omission of SD sequence preceding the start codon did not change the NucB yield appreciably. Lengthening the SD sequence does not result in lower reporter protein level as in *E. coli* [5], [6] where it was suggested that strong SD duplex deriving from mRNA and 16S rRNA stalls the ribosome and thereby slows the translation. This poses the question whether *P. bryantii* is able to form the above mentioned duplex at all. We addressed the issue by constructing series of start codon upstream sequences which trap the SD sequence in secondary structures of different stability. It was found that the secondary structure with the folding energy of -5,2 kcal/mol reduced the NucB protein level in *P. bryantii* TC1-1 to approximately one fifth, whereas in *Escherichia coli* the protein level produced from *hMGFP* preceded by the identical upstream sequence was not affected. The latter is in the agreement with de Smit and van Duin [3] who found that secondary structures less stable than -6 kcal/mol usually don't affect translation initiation in *E. coli*. Also, when the *nucB*, PINA_1201 and SD series of upstream sequences driving the translation are compared, the upstream sequence PINA_1201, lacking secondary structure as predicted by mFold, is the most efficient while the SD6 construct which contains a hairpin loop 14 bp upstream of start codon is the least efficient. Taken together, these experiments suggest that the translation in *P. bryantii* and possibly other *Bacteroidetes* is easily inhibited by secondary structure since there is no SD interaction to compensate for it as in *E. coli* [29]. This could therefore explain both the lack of SD sequence in *Bacteroidetes* sequence logos and the % GC drop in start codon regions. We also verified that the latter leads to a diminished propensity of these regions in mRNA to fold into stable secondary structures relative to intra gene methionine codon upstream regions (Fig 3, table S2) thereby favoring the translation initiation at the authentic start codon.

The % GC drop in genome regions preceding the start codons and the sequence logos of these regions, which lack the SD sequence, clearly separate *Bacteroidetes* and *Chlorobi* from other phyla which is in agreement with a notion that these two groups evolved from a common ancestor not shared by other bacteria [14]. In results we showed that merely by visual inspection of sequence logos one could discriminate genera and families in *Bacteroidetes*. It is similar in *Spirochaetes* where the sequence logo clearly separates *Leptospiraceae* from *Spirochaetaceae* and *Brachyspiraceae* (Fig. S11) and more such cases can be found. If each position in the logo was represented numerically, by its information content and by frequencies of individual bases at each position, one could construct a signature sequence logo along with deviations for each position at the genus or family level which could be used as an additional taxonomic marker. At the phylum level, however, the logo may not be very conserved as exemplified by % AT rich parasites and symbionts in *Proteobacteria* and *Firmicutes*. Excluding such, the GC genome content at the phylum level still varies enough to introduce bias in a sequence logo e.g. *Moorella thermoacetica* and *Listeria monocytogenes* with 55,8 and 38% GC, respectively. This two organisms share a similar shape of SD sequence region, yet the *L. monocytogenes* logo is enriched by AT (Fig. S9) and thus drastically changed as a whole. A correction for % GC deviation at the level of phylum seems needed yet it is not justified since it is evident that the % GC is only one of the factors influencing the start codon upstream region make up. Consequently the shape and placement of SD sequence region and any additional phylum-wide peculiarity of a logo, not the whole logo including its height, is a better candidate marker distinguishing at least some additional bacterial phyla.

## Materials and Methods

### Strains, plasmid, primers and growth conditions

The *P. bryantii* TC1-1 [30] was cultured anaerobically at 37°C in rumen fluid containing M2 medium [31] or modified DSMZ medium 330 [19]. When appropriate, tetracycline was added to the medium prior to inoculation at the final concentration of 3.75 µg ml^−1^. The shuttle vector pRH3 [32] originates from the laboratory of Harry J. Flint (Rowett Research Institute, Aberdeen, Scotland). The phMGFP vector coding for Monster green fluorescent protein was obtained from Promega (USA). The *Escherichia coli* TOP10 and pUC19 vector were from Invitrogen (USA). The oligonucleotide primers were synthesized at Eurofins MWG OPERON (Germany) and Microsynth GmbH (Switzerland) and are shown in table S3.

### Bioinformatic tools

Nucleotide sequences of bacterial genomes were obtained from the EBI Genomes server (http://www.ebi.ac.uk/genomes/). When the unfinished, but annotated whole genome shotgun data for *Bacteroidetes* genomes was examined, the contigs were joined using the union of the EMBOSS package [33]. The 30 bp of upstream sequence of all coding sequences in a given genome were retrieved with Artemis v9 [34]. Sequence logos were then created by WebLogo [35] and the information axis was limited to 1 bit for clarity. The logo position 30 corresponds to nucleotide site directly preceding the start codon. RNA secondary structure was predicted by mfold 3,2 [20]. The nucleotide sequences of 16S rRNA coding genes from the *Bacteroidetes*, *Chlorobi* and other phyla representative genomes were extracted using Artemis and aligned by ClustalX 2.0.12 [36]. The folding energies of the start codon and intra gene methionine codon 30 bp long upstream regions in genome were calculated using UNAFold's hybrid-ss-min [37]. The intra gene mathionine upstream sequences were obtained using Artemis v9.

### PCR, cloning and expression of the *nucB* and *hMGFP* genes

These were essentially performed as described earlier [19]. Briefly, the *nucB* gene variants containing different upstream sequences were produced by PCR, cloned into pRH3, checked for orientation, protected toward *P. bryantii* TC1-1 type II restriction and electroporated into *P. bryantii* TC1-1. The plasmid DNA of the prepared constructs was isolated, sequenced and inspected. The recombinant strains expressed *nucB* in modified DSMZ 330 medium containing tetracycline for 24 hours upon subculturing from overnight culture. All *nucB* constructs contained a stop codon immediately after the *Pst*I site in order to terminate any possible translation coming from the truncated *rteA* gene which was cloned into pRH3 during its construction along with *tetQ*. Constructs with added or omitted SD sequence had 41 bp of upstream sequence. The constructs containing variable SD sequence length, namely SD10, SD8 and SD6, had a 41, 39 and 37 long upstream sequence, respectively. The constructs containing SD sequence involved in secondary structures had 50 bp of upstream sequence.

*hMGFP* variants were also produced by PCR. These *hMGFP* variants contained the same upstream regions as *nucB* variants containing SD sequence involved in secondary structure, except for a cytosine which was inserted immediately downstream of *Pst*I site in order to bring the stop codon following it in frame to end the translation of the lacZ alpha fragment of pUC19 (Fig 2 C). The *hMGFP* variants were digested using *Pst*I, ligated with pUC19 and transformed into *E. coli* TOP10. The resulting recombinant strains were checked for orientation using PCR and verified by sequencing. The strains were grown overnight in LB containing 100 µg/ml of ampicilin, diluted 100x into fresh medium containing ampicillin and grown to OD_600_ = 0,5. For western blot, cells from 0,1 ml of culture were analyzed using anti-his_6_ antibodies since His_6_ was added to MGFP at the C-terminal end. For fluorescence measurement, 1 ml of culture was centrifuged and the cells were resuspended in 0,1 ml of 50 mM Tris-HCl pH = 7,5 0,15 mM NaCl. 50 µl of cell suspension was then transferred to 384 well transparent bottom microplate (Brand, Germany) and the MGFP was excited using blue light converter placed over the transiluminator of Chemigenius^2^ bio imaging system (Syngene, UK).The fluorescence was quantified using GeneTools from Syngene from at least three independent experiments. Prior to that the cultures expressing *hMGFP* were checked using epifluorescence microscopy to assure all cells fluoresce i.e. the plasmid constructs were not lost during cultivation.

### *nucB* and *hMGFP* mRNA quantification

The amount of *nucB* mRNA in *P. bryantii* TC1-1 harboring pRH3 constructs and *hMGFP* m RNA in *E. coli* was measured as described earlier [19] during the exponential growth at OD_600_ = 0,5. Briefly: the total RNA was reverse transcribed using specific primers and cDNA obtained was quantified by real-time PCR using standard curve method and Sybr green as the reporter dye. The amount of the 16S rRNA amplicon was used for *nucB* normalization. Real-time PCR quantification was performed for each sample in triplicates in at least three real-time PCR runs. For *hMGFP* mRNA quantitation new primers were designed: mgrtF and mgrtR amplyfing the *hMGFP* and ecort16SF and ecort16SR for endogenous control which was used for normalization (Table S3).

### NucB and MGFP quantification using western blot

The *P. bryantii* culture supernatans containing NucB were concentrated using Amicon ultra-4 10000 MWCO centrifugal filter devices from Millipore and proteins separated by standard SDS-PAGE gel. When analysing MGFP content, the cells from 0,1 ml of culture were centrifuged and resuspenced in 50 µl of water. The western blot and immunodetection was performed as described earlier [19] except for the change of the HRP substrate, which was SuperSignal from Novagen (Merck, Germany). The chemiluminiscence was recorded using the Chemigenius^2^ bio imaging system (Syngene, UK) and relative quantification was done using GeneTools from Syngene from at least three independent experiments.

## Supporting Information

Figure S1 **Sequence logos of start codon upstream regions of** ***Acidobacteria*** **.**
(DOC)

Figure S2 **Sequence logos of start codon upstream regions of** ***Aquificae*** **.**
(DOC)

Figure S3 **Sequence logos of start codon upstream regions of** ***Chlamydiae*** **.**
(DOC)

Figure S4 **Sequence logos of start codon upstream regions of** ***Chlorobi*** **.**
(DOC)

Figure S5 **Sequence logos of start codon upstream regions of** ***Bacteroidetes*** **.**
(DOC)

Figure S6 **Sequence logos of start codon upstream regions of** ***Chloroflexi*** **.**
(DOC)

Figure S7 **Sequence logos of start codon upstream regions of** ***Cyanobacteria*** **.**
(DOC)

Figure S8 **Sequence logos of start codon upstream regions of** ***Fibrobacteres*** **.**
(DOC)

Figure S9 **Sequence logos of start codon upstream regions of** ***Firmicutes*** **.**
(DOC)

Figure S10 **Sequence logos of start codon upstream regions of** ***Proteobacteria*** **.**
(DOC)

Figure S11 **Sequence logos of start codon upstream regions of** ***Spirochaetae*** **.**
(DOC)

Figure S12 **Sequence logos of start codon upstream regions of** ***Thermotogae*** **.**
(DOC)

Figure S13 **Sequence logos of start codon upstream regions of** ***Deinococcus-Thermus*** **.**
(DOC)

Figure S14 **Sequence logos of start codon upstream regions of** ***Actinobacteria*** **.**
(DOC)

Figure S15 **Sequence logos of start codon upstream regions of** ***Tenericutes*** **.**
(DOC)

Figure S16 **Multiple sequence alignment of 16S rRNA genes from** ***Bacteroidetes*** **and representatives of other bacterial phyla.**
(PDF)

Figure S17 **mRNA secondary structure prediction of 11,1, 5,2, 2,1, 0, 11,1inv, 5,4inv and -20 start codon upstream regions.**
(DOC)

Figure S18 **A typical western blot used for quantification.**
(DOC)

Figure S19 **mRNA secondary structure prediction of PINA_1201,** ***nucB*** **, SD6, SD8 and SD10 start codon upstream regions.**
(DOC)

Table S1 **GC content in the start codon upstream regions in major bacterial phyla.**
(DOC)

Table S2 **Folding energy of the annotated start codon and successive methionine codons 30 bp upstream regions in genus** ***Prevotella*** **,** ***Escherichia coli*** **K12 MG1655 and** ***Bacillus subtilis*** **subsp.** ***subtilis*** **168.**
(XLS)

Table S3 **Primers used in the study.**
(DOC)

## References

[1] Kozak M (2005). Regulation of translation via mRNA structure in prokaryotes and eukaryotes.. Gene.

[2] Laursen BS, Sørensen HP, Mortensen KK, Sperling-Petersen HU (2005). Initiation of protein synthesis in bacteria.. Microbiol Mol Biol Rev.

[3] de Smit MH, van Duin J (1994). Control of translation by mRNA secondary structure in Escherichia coli.. J Mol Biol.

[4] Shultzaberger RK, Bucheimer RE, Russ KE, Schneider TD (2001). Anatomy of Escherichia coli ribosome binding sites.. J Mol Biol.

[5] Komarova AV, Tchufistova LS, Supina EV, Boni IV (2002). Protein S1 counteracts the inhibitory effect of the extended Shine-Dalgarno sequence on translation.. RNA.

[6] Vimberg V, Tats A, Remm M, Tenson T (2007). Translation initiation region preferences in Escherichia coli.. BMC Mol Biol.

[7] Fargo DC, Zhang M, Gillham NW, Boynton JE (1998). Shine-Dalgarno-like sequences are not required for translation of chloroplast mRNAs in Chlamydomonas reinhardtii chloroplasts or in Escherichia coli.. Mol Gen Genet.

[8] Moll I, Grill S, Gualerzi CO, Bläsi U (2002). Leaderless mRNA in bacteria: surprises in ribosomal recruitment and translational control.. Mol Microbiol.

[9] Chang B, Halgamuge S, Tang SL (2006). Analysis of SD sequences in completed microbial genomes: Non-SD-led genes are as common as SD-led genes.. Gene.

[10] Nakagawa S, Niimura Y, Miura K-I, Gojobori T (2010). Dynamic evolution of translation initiation mechanisms in prokaryotes.. Proc Natl Acad Sci U S A.

[11] Salah P, Bisaglia M, Aliprandi P, Uzan M, Sizun C (2009). Probing the relationship between Gram-negative and Gram-positive S1 proteins by sequence analysis.. Nucleic Acids Res.

[12] Schneider TD, Stephens RM (1990). Sequence logos: a new way to display consenus sequences.. Nucleic Acids Res.

[13] Miyazaki K, Miyamoto H, Mercer DK, Hirase TMartin (2003). Involvement of the multidomain regulatory protein XynR in positive control of xylanase gene expression in the ruminal anaerobe Prevotella bryantii B_1_4.. J Bacteriol.

[14] Gupta RS, Lorenzini E (2007). Phylogeny and molecular signatures (conserved proteins and indels) that are specific for the Bacteroidetes and Chlorobi species.. BMC Evol Biol.

[15] Bayley DP, Rocha ER, Smith CJ (2000). Analysis of cepA and other Bacteroides fragilis genes reveals a unique promoter structure.. FEMS Microbiol Lett.

[16] Chen S, Bagdasarian M, Kaufman MG, Walker ED (2007). Characterization of strong promoters from an environmental Flavobacterium hibernum strain by using a green fluorescent protein-based reporter system.. Appl Environ Microbiol.

[17] Vingadassalom D, Kolb A, Mayer C, Rybkine T, Collatz F (2005). An unusual primary sigma factor in the Bacteroidetes phylum.. Mol Microbiol.

[18] Salyers AA, Bonheyo G, Shoemaker NB (2000). Starting a new genetic system: lessons from Bacteroides.. Methods.

[19] Accetto T, Avguštin G (2007). Studies on Prevotella nuclease using a system for the controlled expression of cloned genes in P. bryantii TC1-1.. Microbiol-SGM.

[20] Schluenzen F, Tocilj A, Zarivach R, Harms J, Gluehmann M (2000). Structure of functionally activated small ribosomal subunit at 3.3 Å resolution.. Cell.

[21] Zuker M (2003). Mfold web server for nucleic acid folding and hybridization prediction.. Nucleic Acids Res.

[22] Cerdeño-Tárraga AM, Patrick S, Crossman LC, Blakely G, Abratt V (2005). Extensive DNA inversions in the B. fragilis genome control variable gene expression.. Science.

[23] Kuwahara T, Yamashita A, Hirakawa H, Nakayama H, Toh H (2004). Genomic analysis of Bacteroides fragilis reveals extensive DNA inversions regulating cell surface adaptation.. Proc Natl Acad Sci U S A.

[24] Xu J, Bjursell MK, Himrod J, Deng S, Carmichael LK (2003). A genomic view of the human-Bacteroides thetaiotaomicron symbiosis.. Science.

[25] Xu J, Mahowald MA, Ley RE, Lozupone CA, Hamady M (2007). Evolution of symbiotic bacteria in the distal human intestine.. PLoS Biol.

[26] Sakamoto M, Benno Y (2006). Reclassification of Bacteroides distasonis, Bacteroides goldsteinii and Bacteroides merdae as Parabacteroides distasonis gen. nov., comb. nov., Parabacteroides goldsteinii comb. nov. and Parabacteroides merdae comb. nov.. Int J Syst Evol Microbiol.

[27] Zhu HQ, Hu GQ, Ouyang ZQ, Wang J, She ZS (2004). Accuracy improvement for identifying translation initiation sites in microbial genomes.. Bioinformatics.

[28] Nakamoto T (2007). The initiation of eukaryotic and prokaryotic protein synthesis: A selective accessibility and multisubstrate enzyme reaction.. Gene.

[29] de Smit MH, van Duin J (1994). Translational initiation on structured messengers. Another role for the Shine-Dalgarno interaction.. J Mol Biol.

[30] van Gylswyk NO (1990). Enumeration and presumptive identification of some functional groups of bacteria in the rumen of dairy cows fed grass silage-based diets.. FEMS Microbiol Ecol.

[31] Hobson PN, Norris JR, Ribbons DW (1969). Rumen bacteria.. Methods in microbiology, Vol 3B.

[32] Daniel AS, Martin J, Vanat I, Whitehead TR, Flint HJ (1995). Expression of a cloned cellulase/xylanase gene from Prevotella ruminicola in Bacteroides vulgatus, Bacteroides uniformis and Prevotella ruminicola.. J Appl Bacteriol.

[33] Rice P, Longden I, Bleasby A (2000). EMBOSS: The European Molecular Biology Open Software Suite.. Trends Genet.

[34] Rutherford K, Parkhill J, Crook J, Horsnell T, Rice P (2000). Artemis: sequence visualization and annotation.. Bioinformatics.

[35] Crooks GE, Hon G, Chandonia JM, Brenner SE (2004). WebLogo: a sequence logo generator.. Genome Res.

[36] Larkin MA, Blackshields G, Brown NP, Chenna R, McGettigan PA (2007). Clustal W and Clustal X version 2.0.. Bioinformatics.

[37] Markham NR, Zuker M, Keith JM (2008). UNAFold: software for nucleic acid folding and hybriziation.. Bioinformatics, Volume II.

