# Supplementary material for: Inability of Prevotella bryantii to Form a Functional Shine-Dalgarno Interaction Reflects Unique Evolution of Ribosome Binding Sites in Bacteroidetes
Source: PLoS One. 2011 Aug 12;6(8):e22914. doi: 10.1371/journal.pone.0022914 (PMC3155529; doi:10.1371/journal.pone.0022914)
Supplement: Figure S1 — Sequence logos of start codon upstream regions of Acidobacteria . (DOC) [file pone.0022914.s001.doc]

***ACIDOBACTERIA***


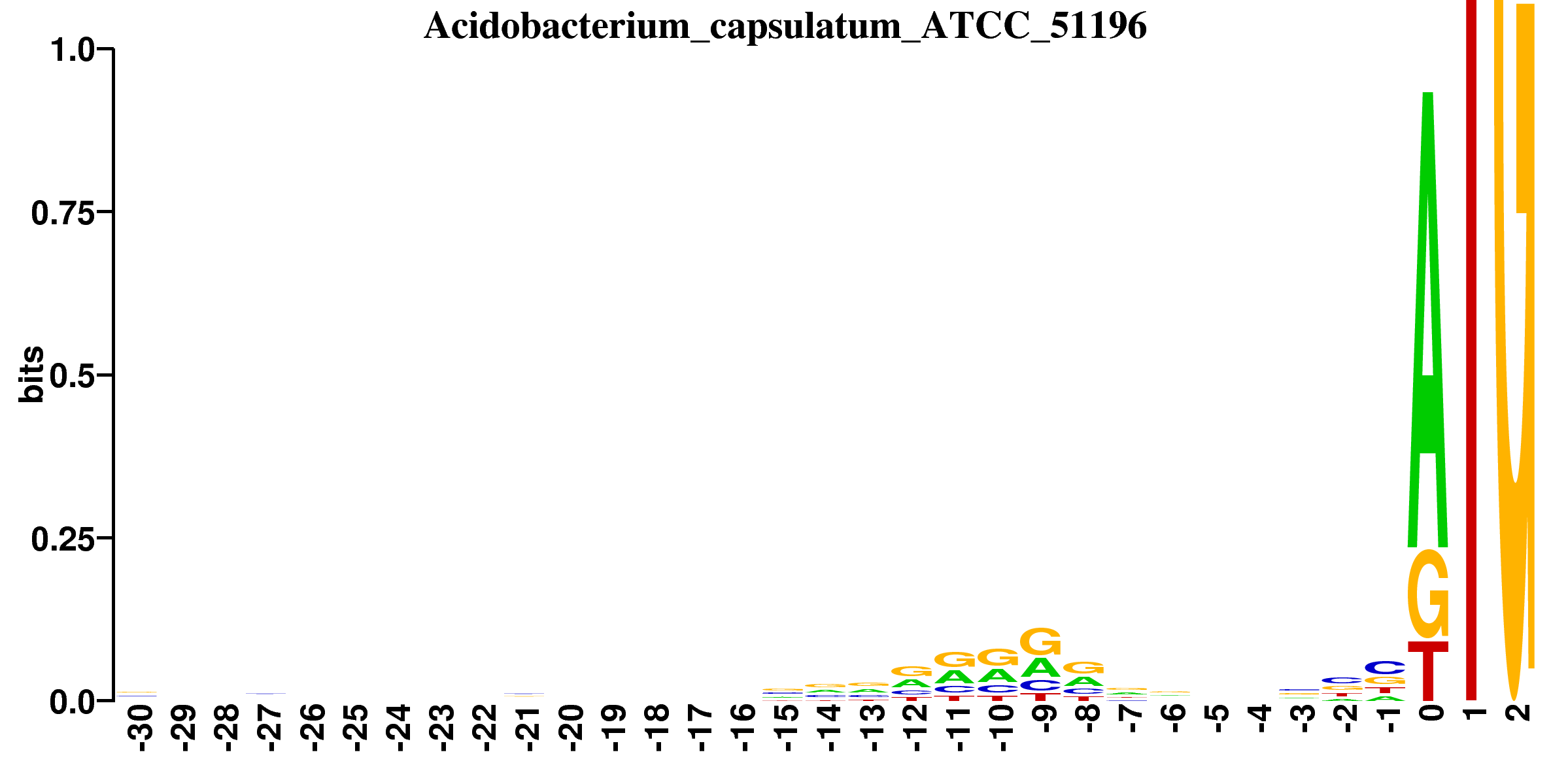


| genome % GC | start codon upstream region % GC | difference %GC | genome size [ Mb] |
| --- | --- | --- | --- |
| **60,5** | **55,5** | **5** | **4,1** |


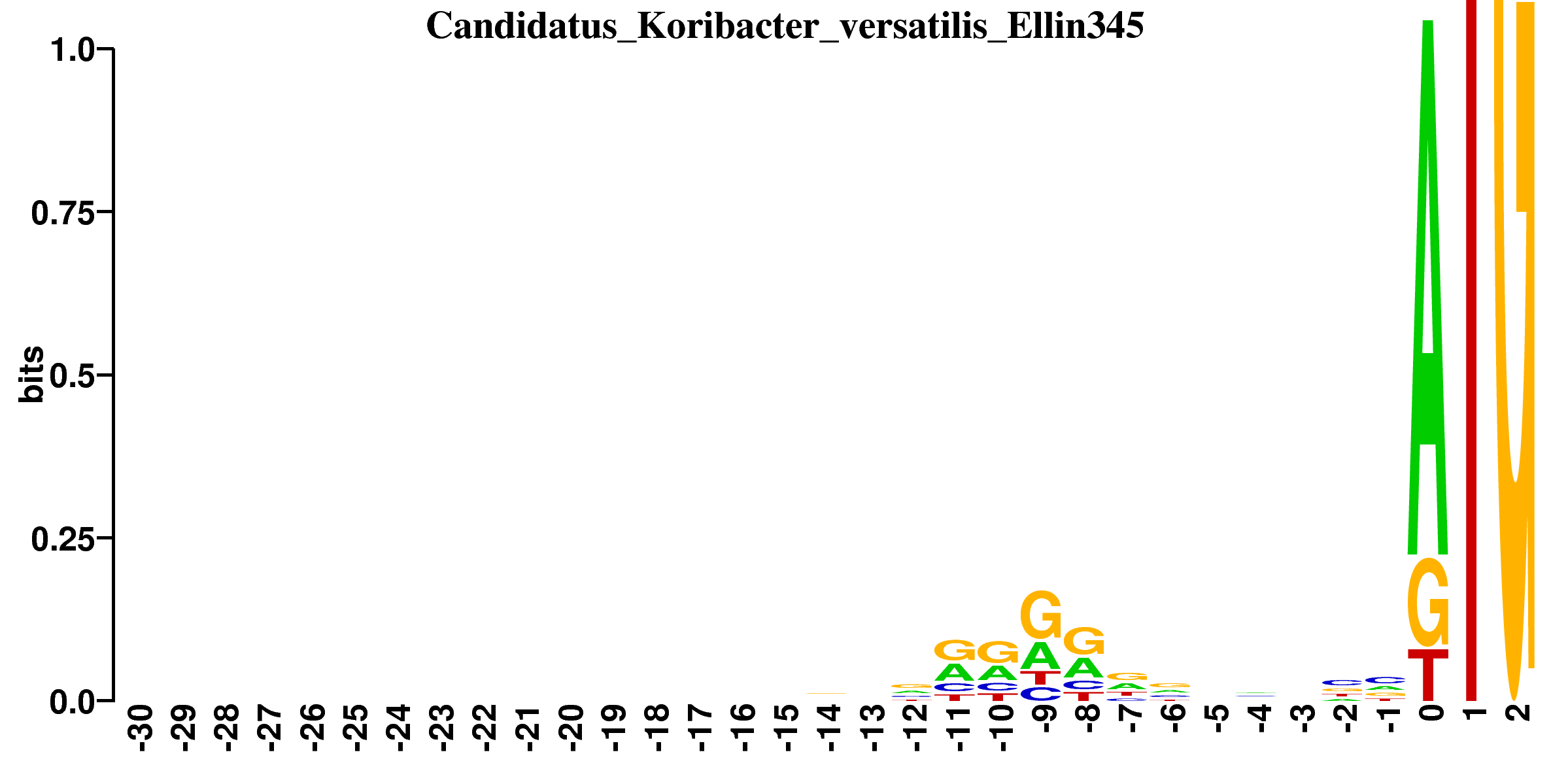


| genome % GC | start codon upstream region % GC | difference %GC | genome size [ Mb] |
| --- | --- | --- | --- |
| **58,4** | **53,9** | **4,5** | **5,8** |


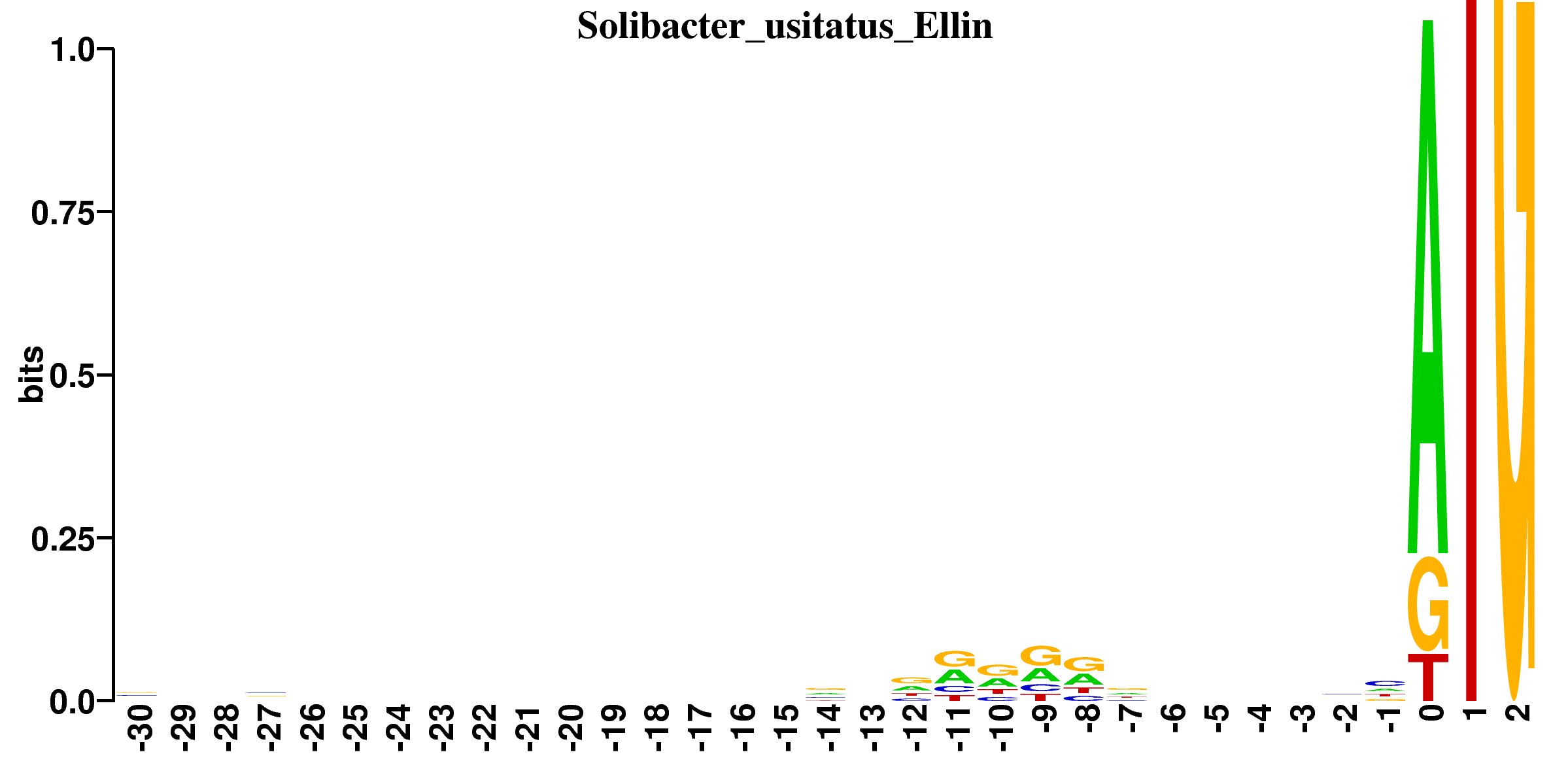


| genome % GC | start codon upstream region % GC | difference %GC | genome size [ Mb] |
| --- | --- | --- | --- |
| **61,9** | **54,1** | **7,8** | **10** |
