# Supplementary material for: Inability of Prevotella bryantii to Form a Functional Shine-Dalgarno Interaction Reflects Unique Evolution of Ribosome Binding Sites in Bacteroidetes
Source: PLoS One. 2011 Aug 12;6(8):e22914. doi: 10.1371/journal.pone.0022914 (PMC3155529; doi:10.1371/journal.pone.0022914)
Supplement: Figure S2 — Sequence logos of start codon upstream regions of Aquificae . (DOC) [file pone.0022914.s002.doc]

***AQUIFICAE***


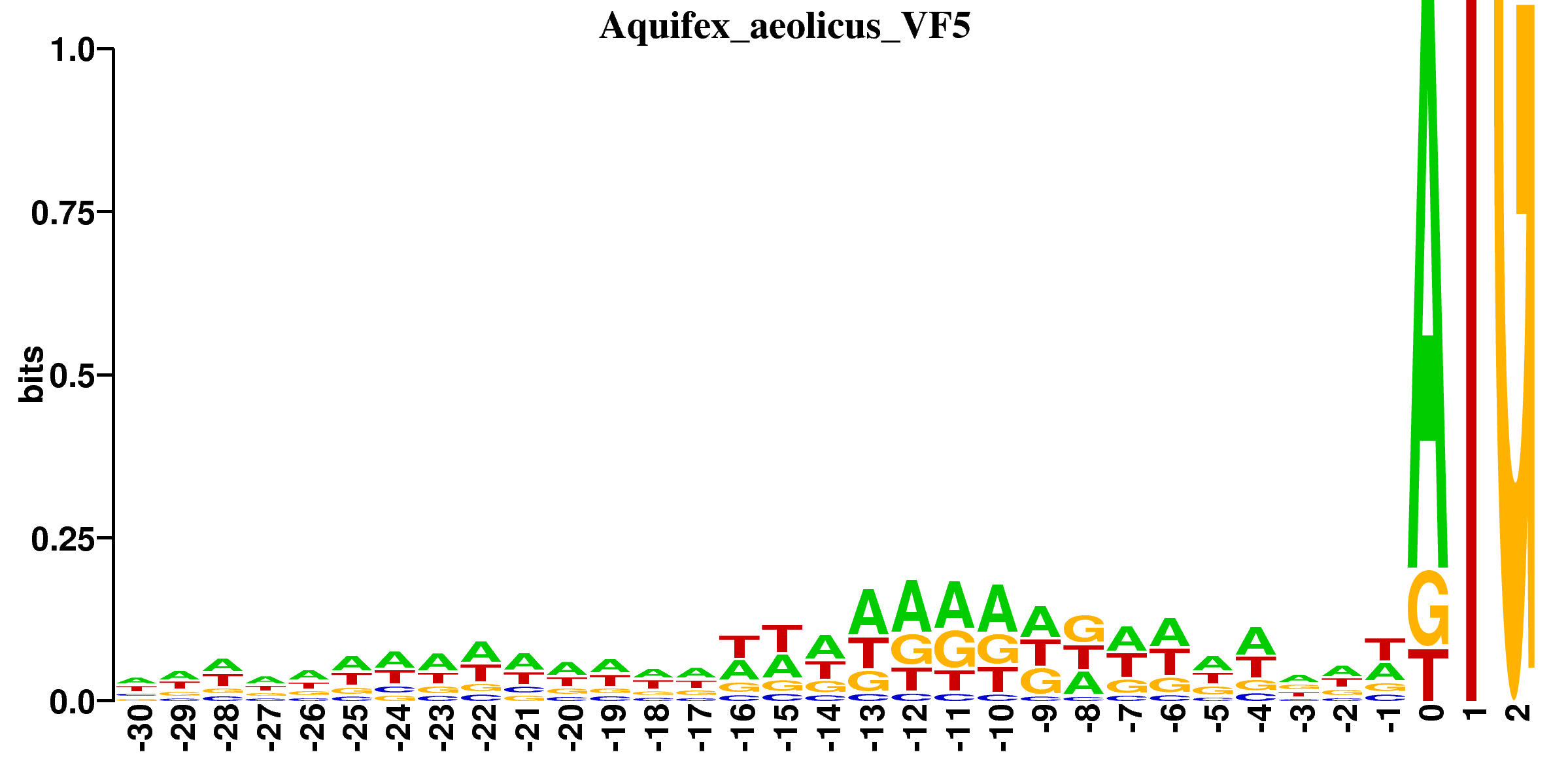


| genome % GC | start codon upstream region % GC | difference %GC | genome size [ Mb] |
| --- | --- | --- | --- |
| **43,5** | **34,8** | **8,7** | **1,6** |


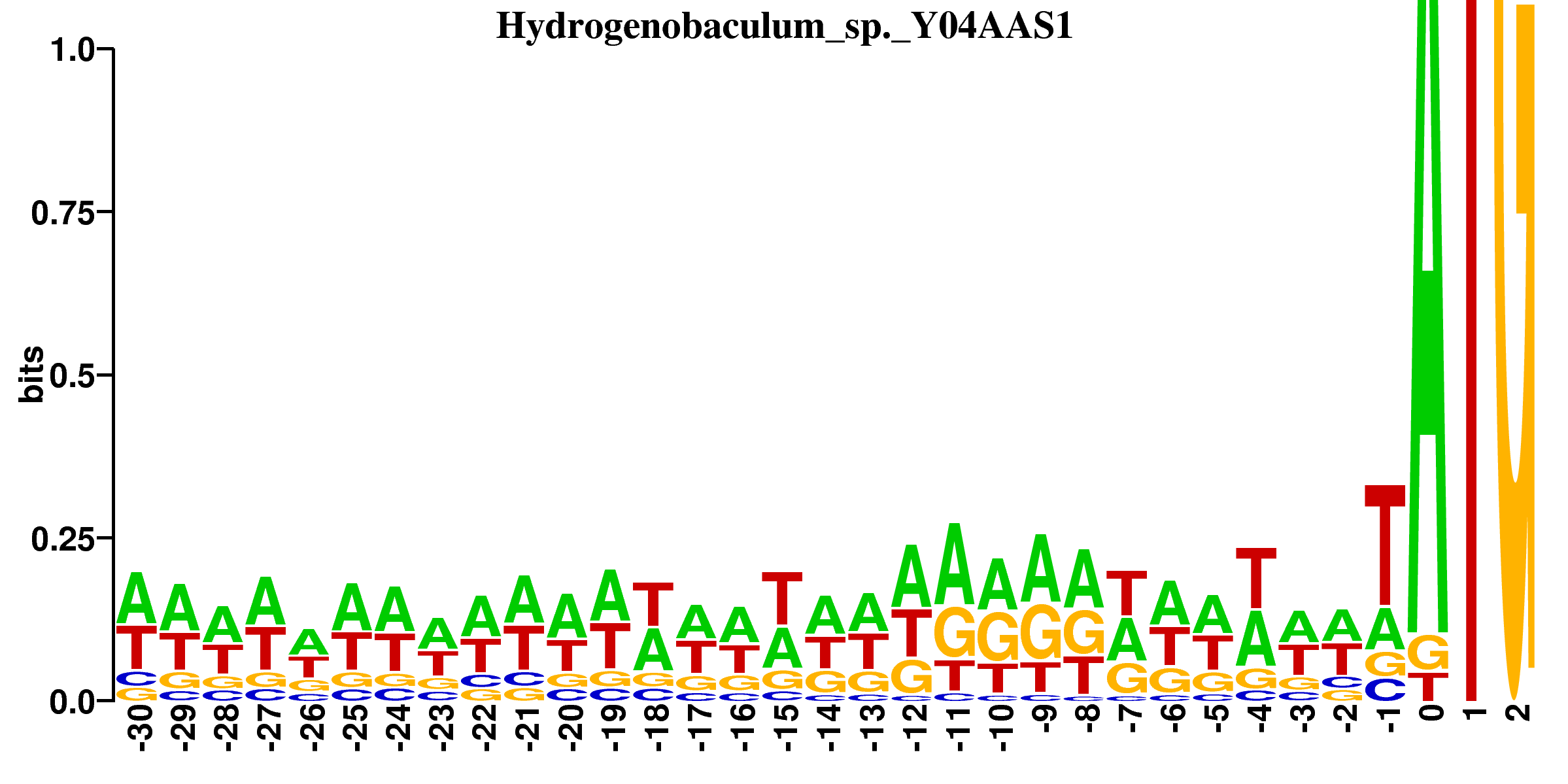


| genome % GC | start codon upstream region % GC | difference %GC | genome size [ Mb] |
| --- | --- | --- | --- |
| **34,8** | **28,5** | **6,3** | **1,6** |


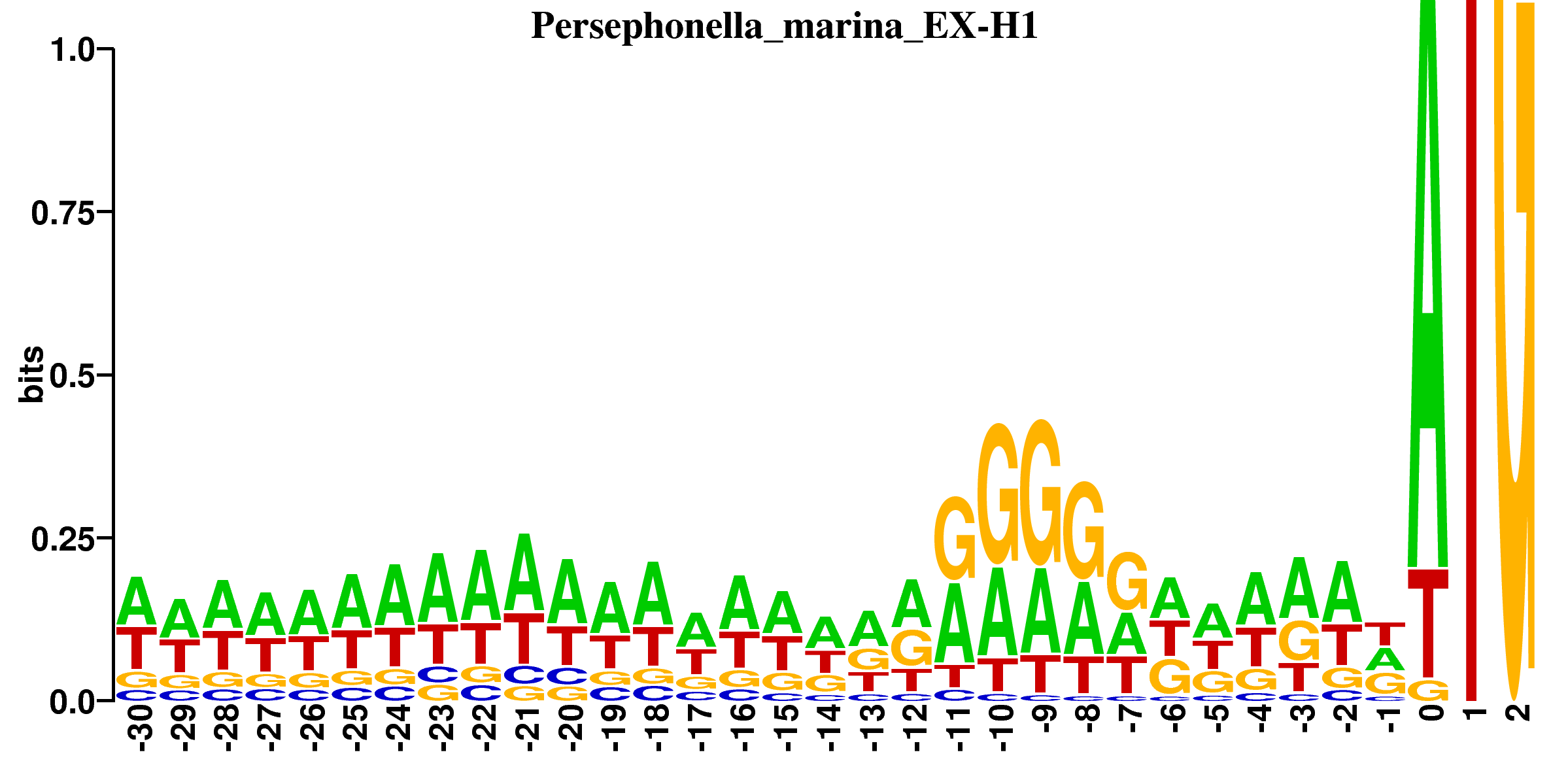


| genome % GC | start codon upstream region % GC | difference %GC | genome size [ Mb] |
| --- | --- | --- | --- |
| **37,2** | **32,1** | **5,1** | **2** |


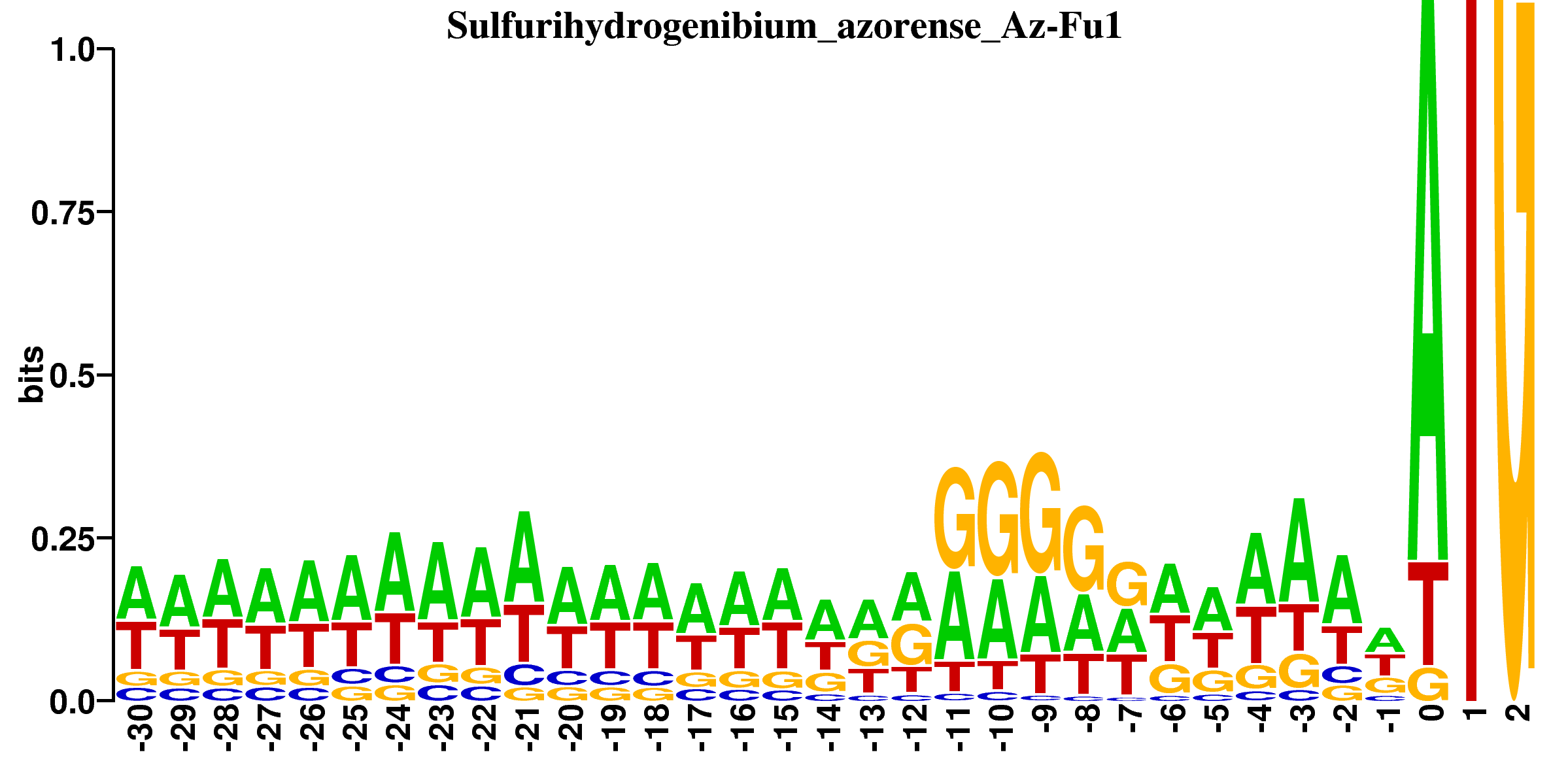


| genome % GC | start codon upstream region % GC | difference %GC | genome size [ Mb] |
| --- | --- | --- | --- |
| **32,8** | **29,4** | **3,4** | **1,6** |
