# Supplementary material for: Inability of Prevotella bryantii to Form a Functional Shine-Dalgarno Interaction Reflects Unique Evolution of Ribosome Binding Sites in Bacteroidetes
Source: PLoS One. 2011 Aug 12;6(8):e22914. doi: 10.1371/journal.pone.0022914 (PMC3155529; doi:10.1371/journal.pone.0022914)
Supplement: Figure S3 — Sequence logos of start codon upstream regions of Chlamydiae . (DOC) [file pone.0022914.s003.doc]

***CHLAMYDIAE***


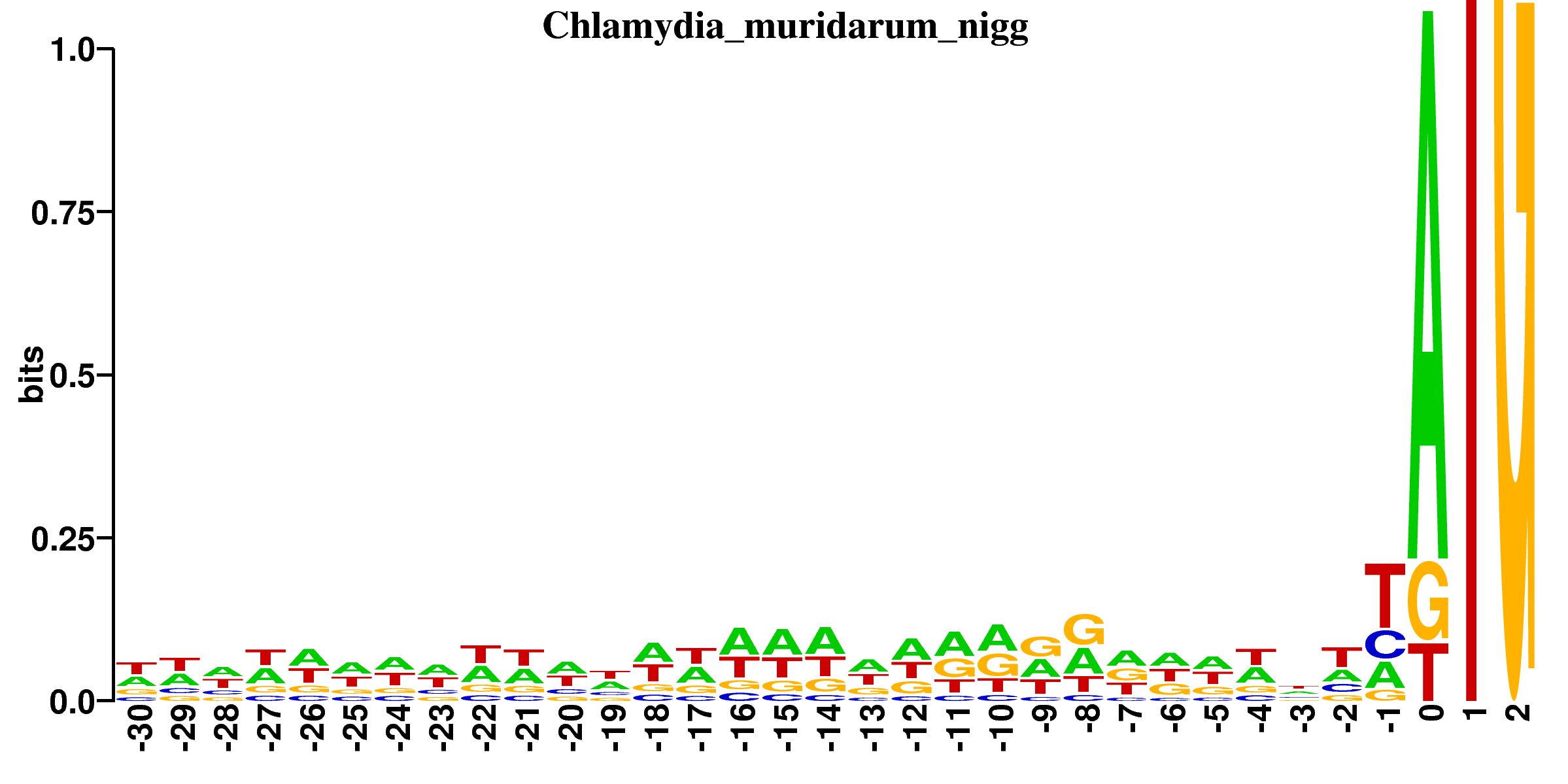


| genome % GC | start codon upstream region % GC | difference %GC | genome size [ Mb] |
| --- | --- | --- | --- |
| **40,3** | **35,8** | **4,5** | **1,1** |


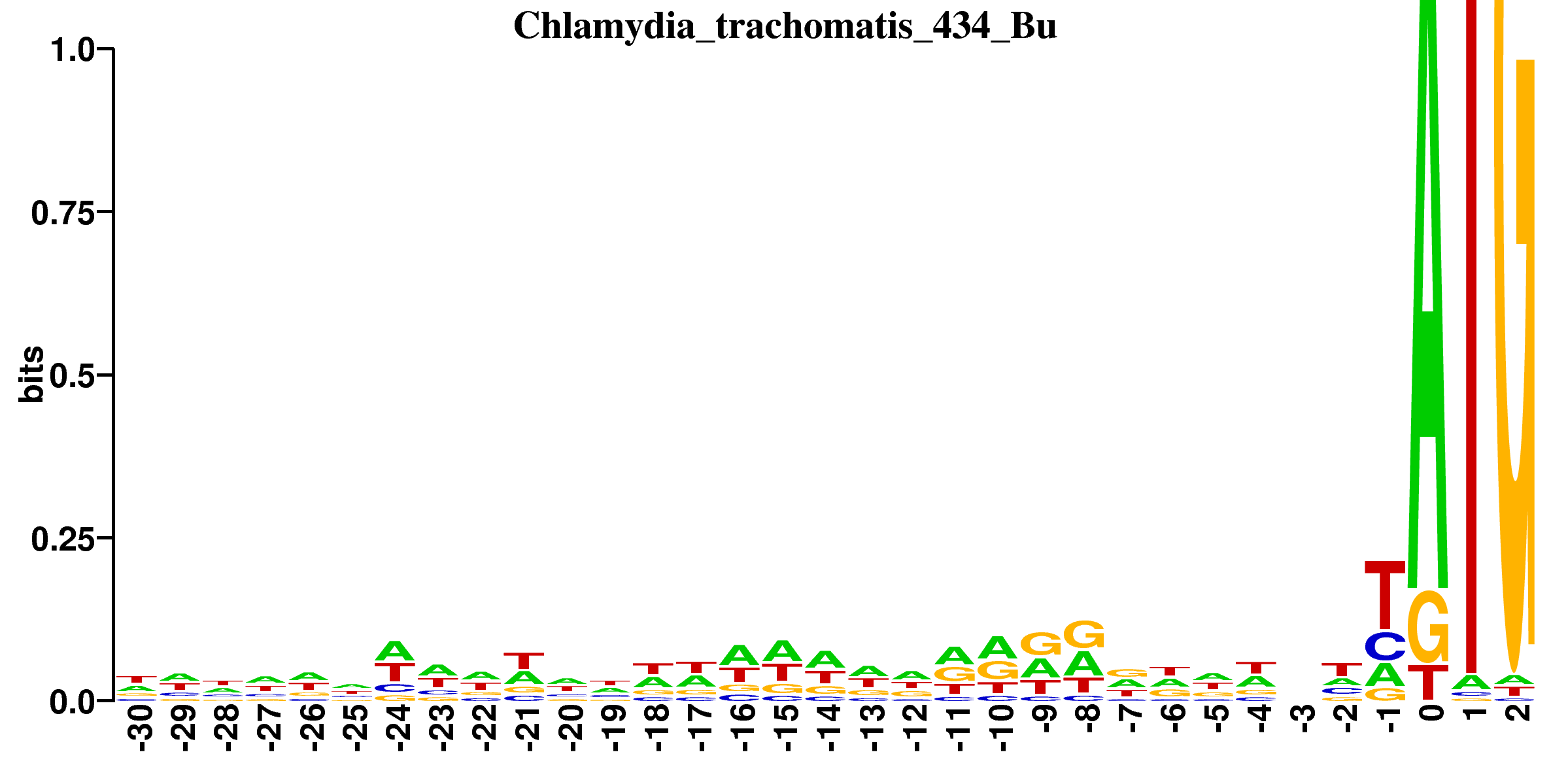


| genome % GC | start codon upstream region % GC | difference %GC | genome size [ Mb] |
| --- | --- | --- | --- |
| **41,3** | **37** | **4,3** | **1** |


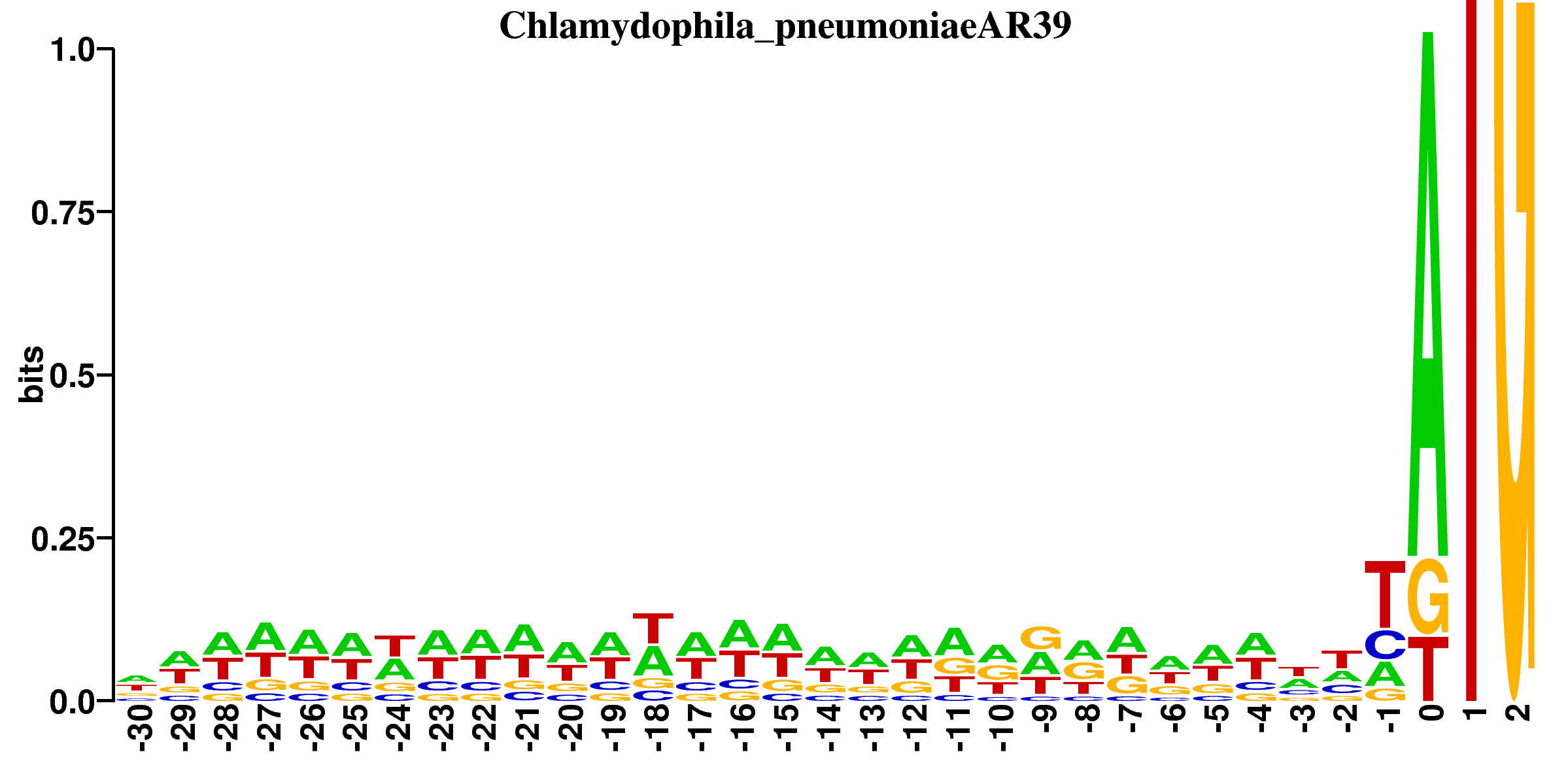


| genome % GC | start codon upstream region % GC | difference %GC | genome size [ Mb] |
| --- | --- | --- | --- |
| **40,6** | **33,4** | **7,2** | **1,23** |


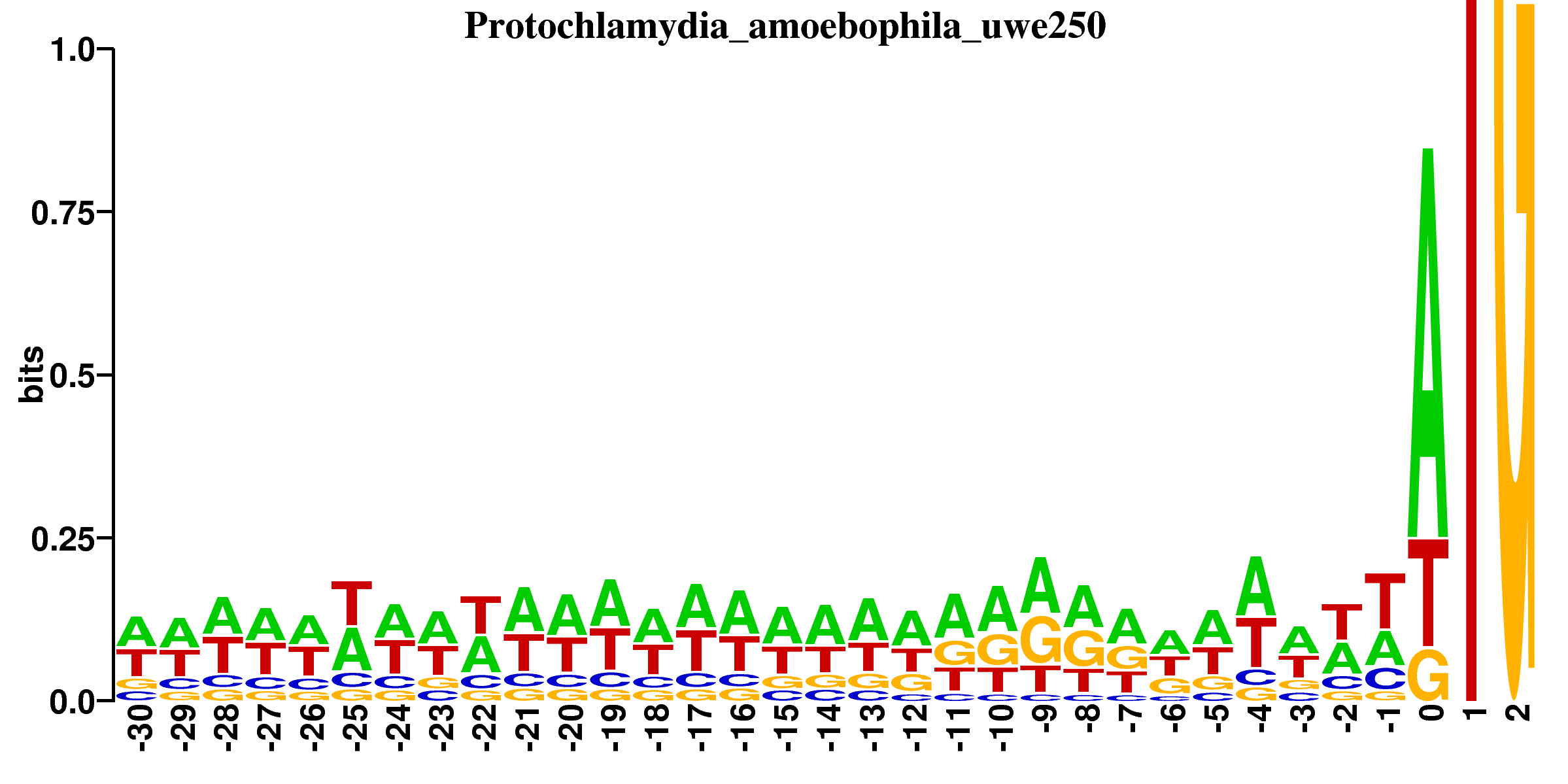


| genome % GC | start codon upstream region % GC | difference %GC | genome size [ Mb] |
| --- | --- | --- | --- |
| **34,7** | **29,4** | **5,3** | **2,41** |
