# Supplementary material for: Inability of Prevotella bryantii to Form a Functional Shine-Dalgarno Interaction Reflects Unique Evolution of Ribosome Binding Sites in Bacteroidetes
Source: PLoS One. 2011 Aug 12;6(8):e22914. doi: 10.1371/journal.pone.0022914 (PMC3155529; doi:10.1371/journal.pone.0022914)
Supplement: Figure S4 — Sequence logos of start codon upstream regions of Chlorobi . (DOC) [file pone.0022914.s004.doc]

***CHLOROBI***


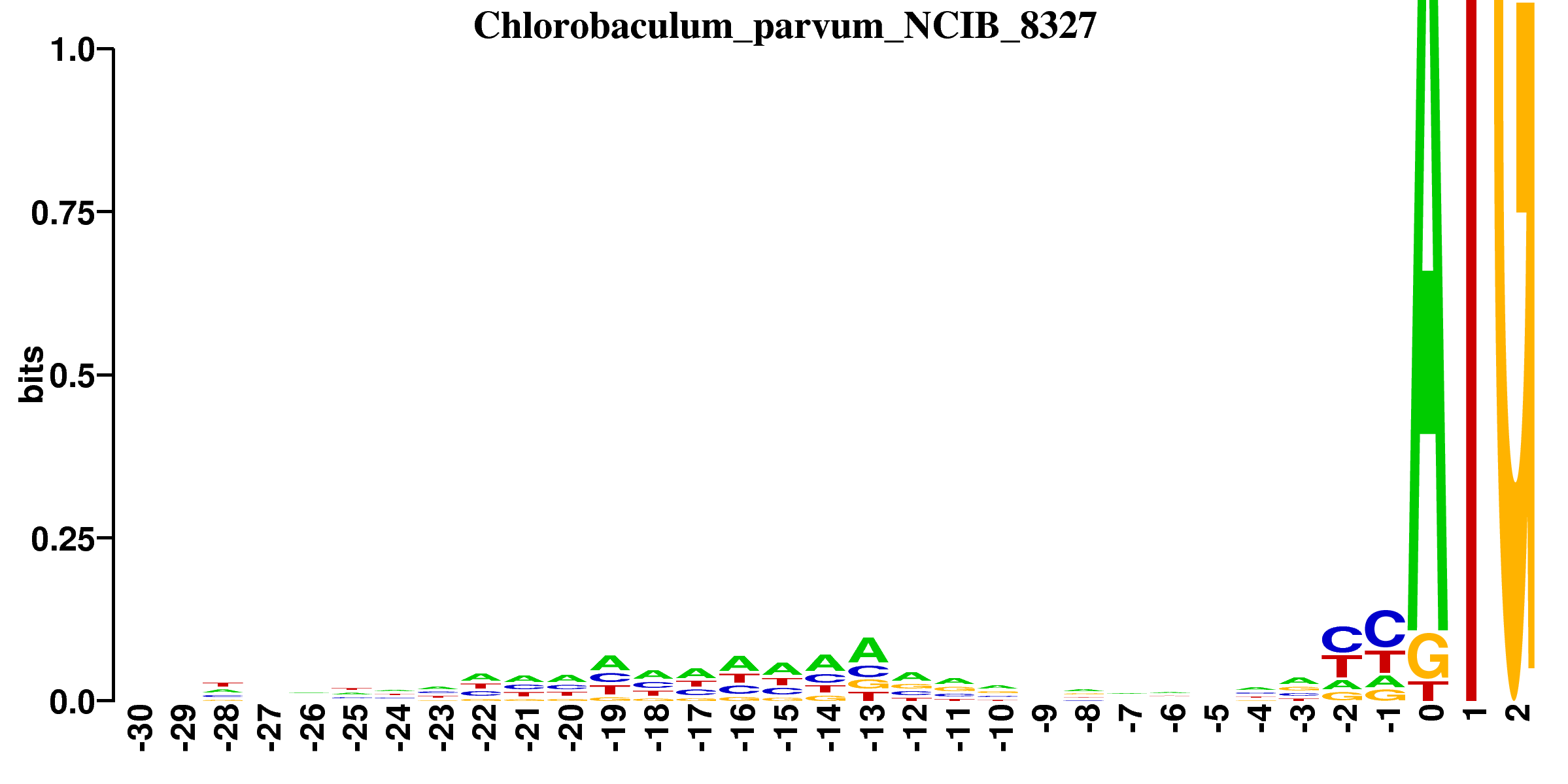


| genome % GC | start codon upstream region % GC | difference %GC | genome size [ Mb] |
| --- | --- | --- | --- |
| **55,8** | **44,6** | **11,2** | **2,3** |


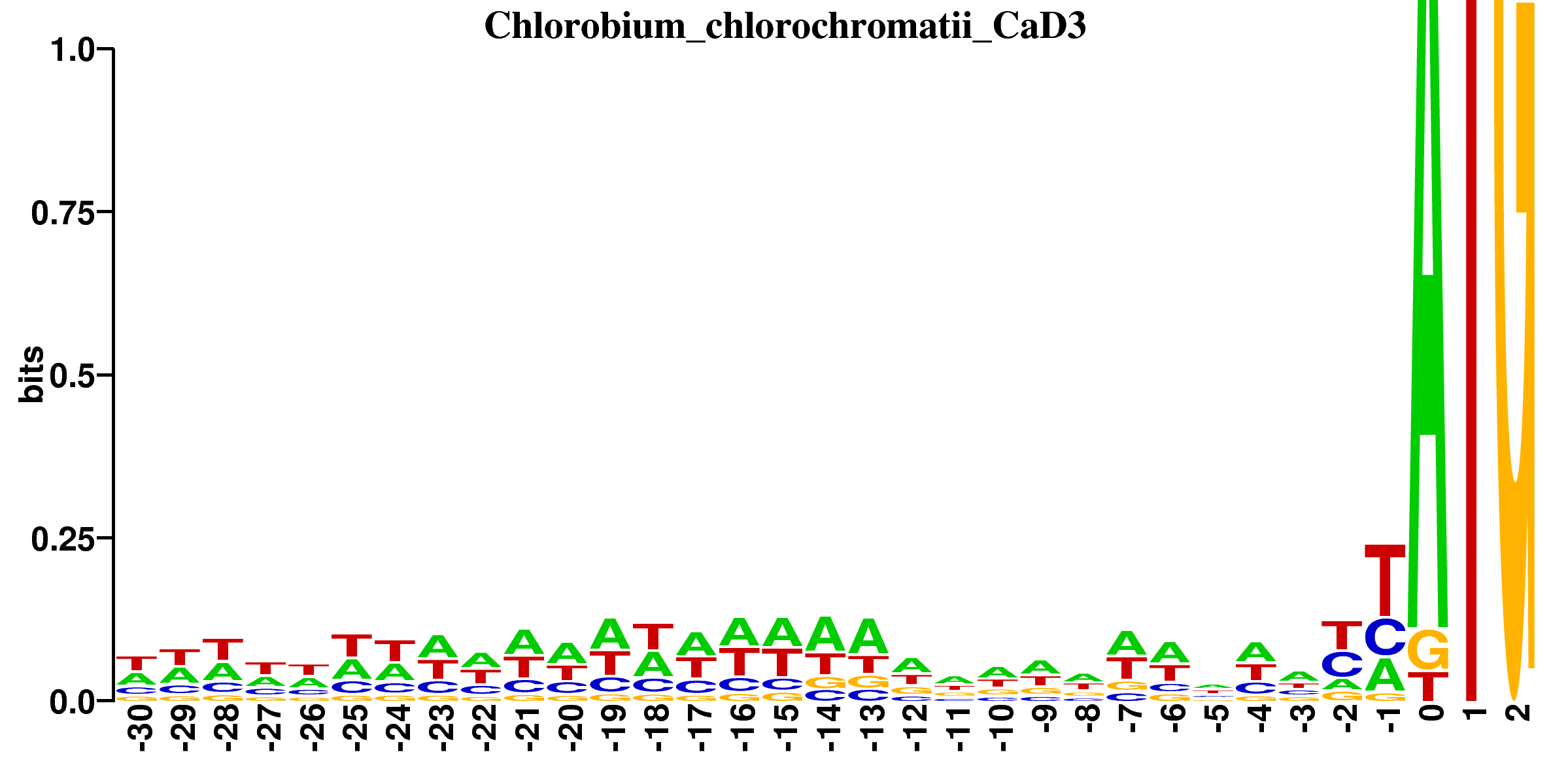


| genome % GC | start codon upstream region % GC | difference %GC | genome size [ Mb] |
| --- | --- | --- | --- |
| 44,3 | 34,7 | 9,6 | 2,6 |


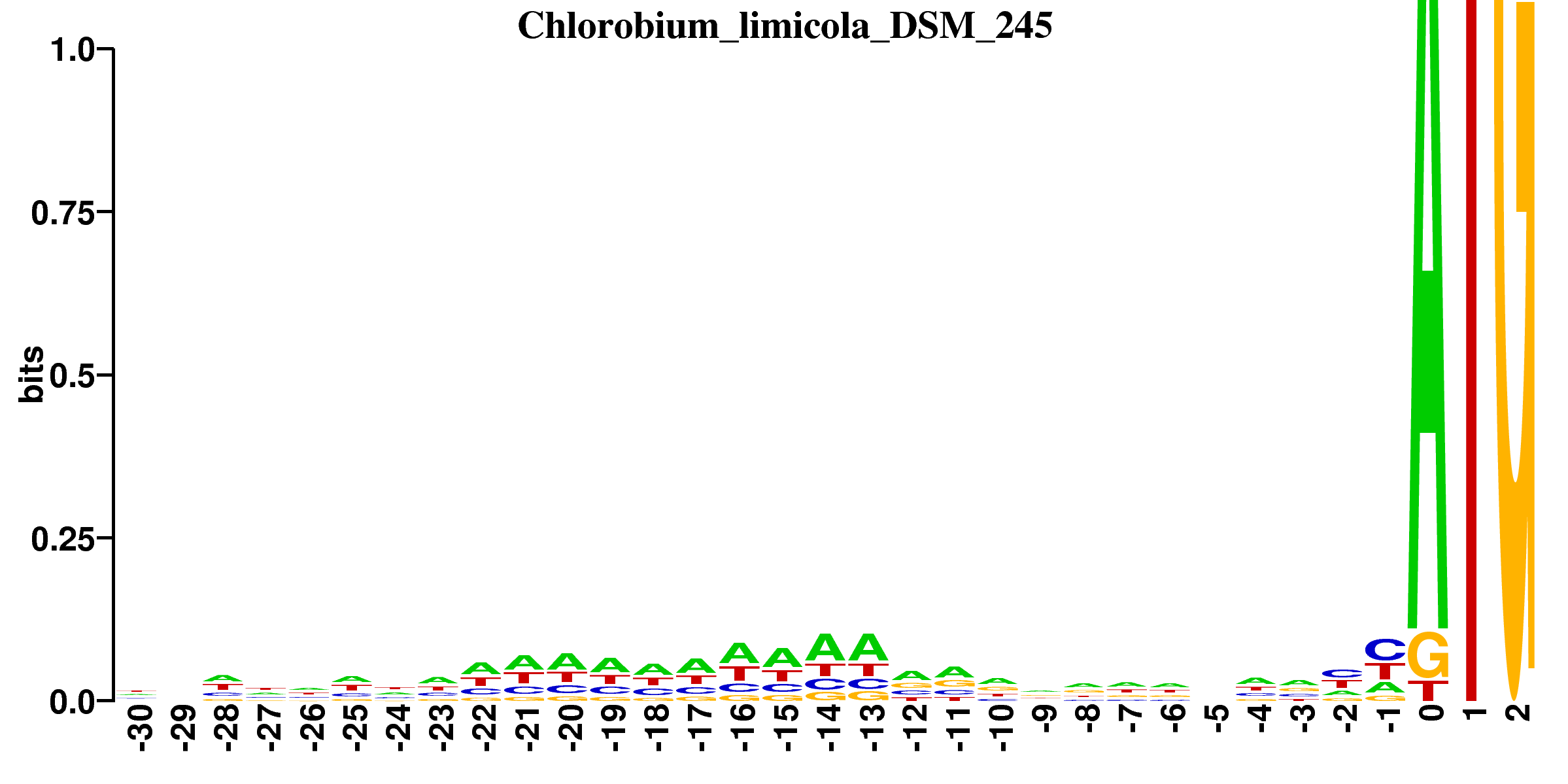


| genome % GC | start codon upstream region % GC | difference %GC | genome size [ Mb] |
| --- | --- | --- | --- |
| 51,3 | 40,9 | 10,4 | 2,8 |


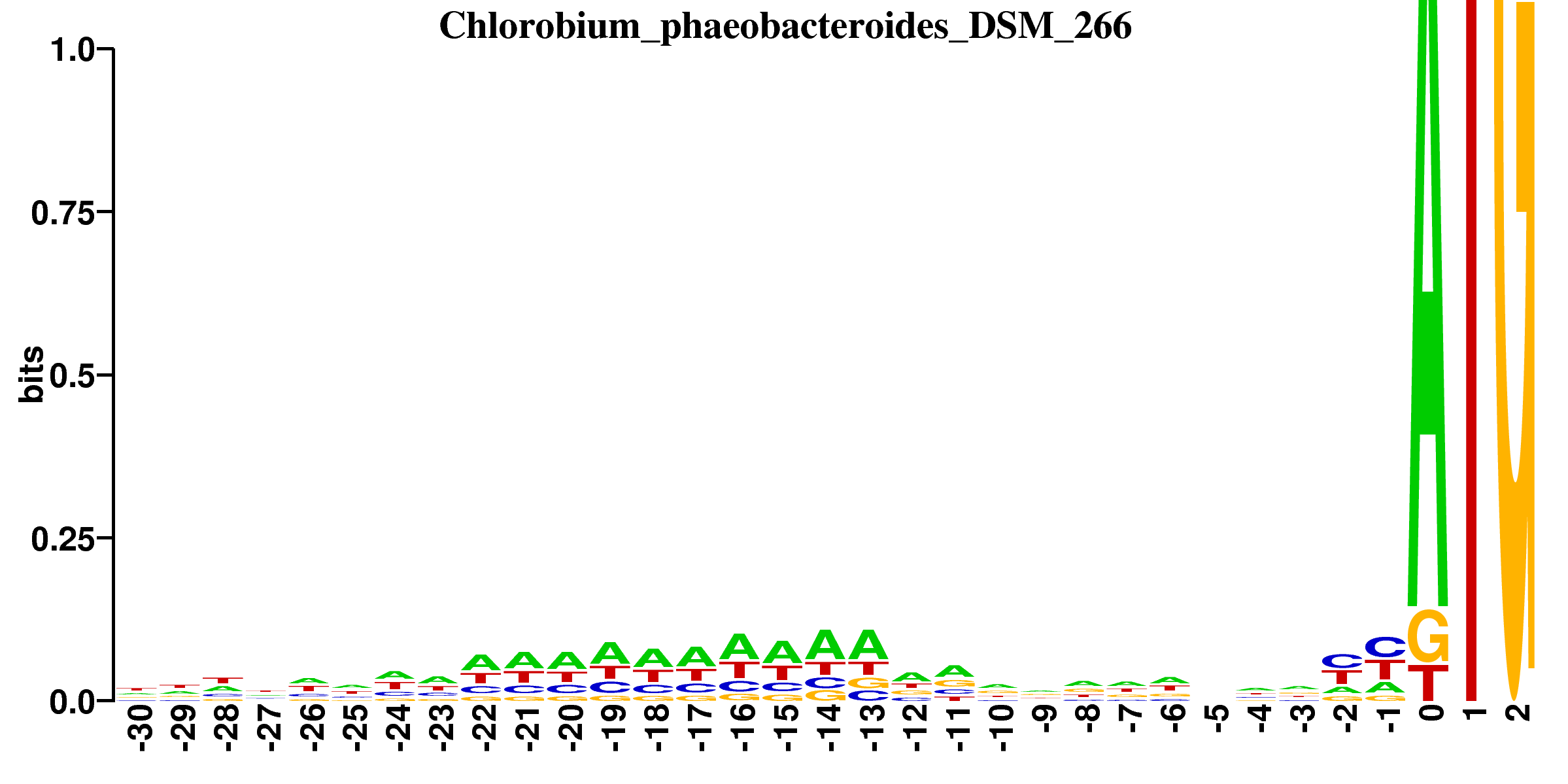


| genome % GC | start codon upstream region % GC | difference %GC | genome size [ Mb] |
| --- | --- | --- | --- |
| 48,9 | 39,6 | 9,3 | 2,7 |


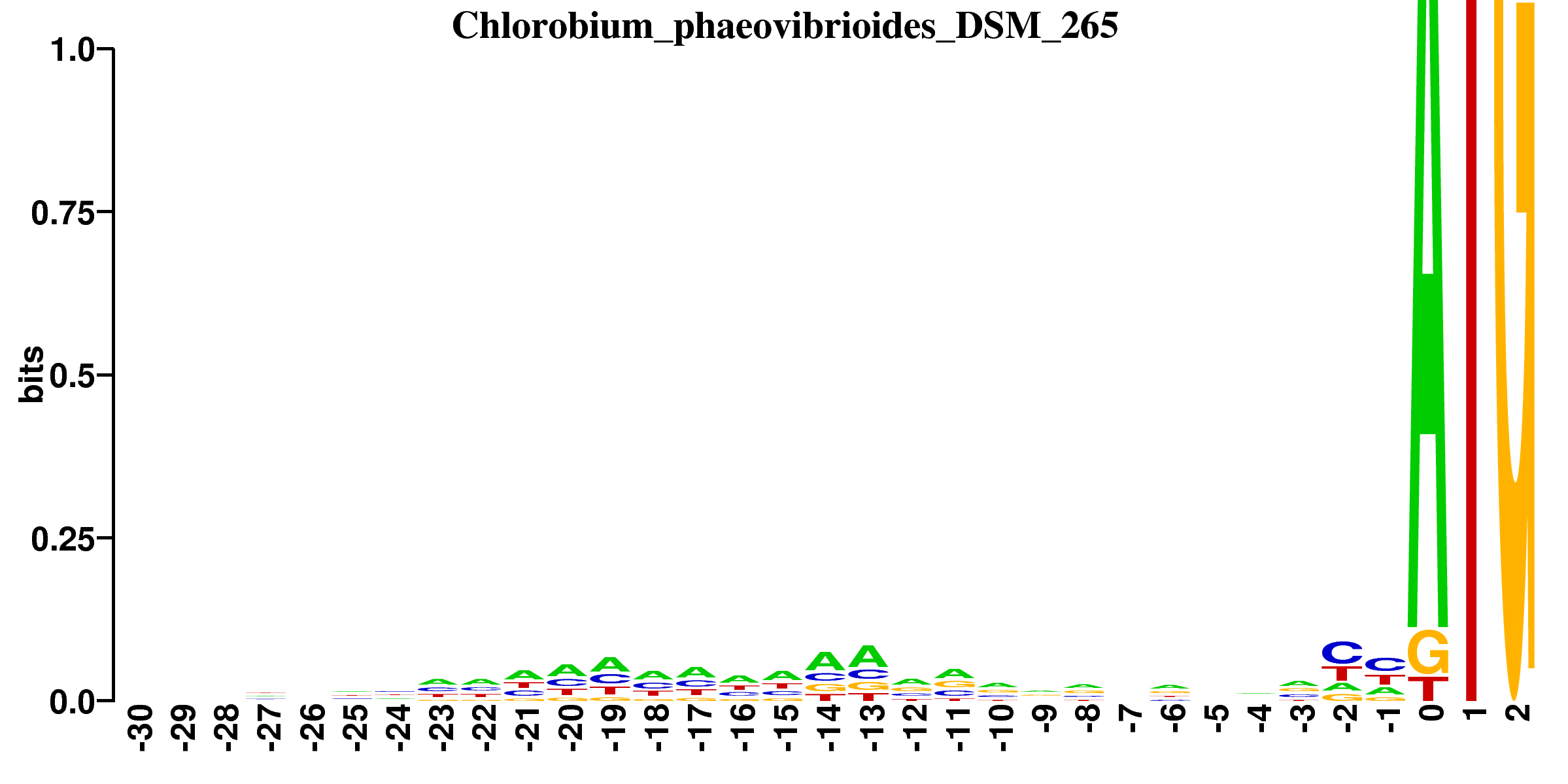


| genome % GC | start codon upstream region % GC | difference %GC | genome size [ Mb] |
| --- | --- | --- | --- |
| 53 | 44,7 | 8,3 | 2 |


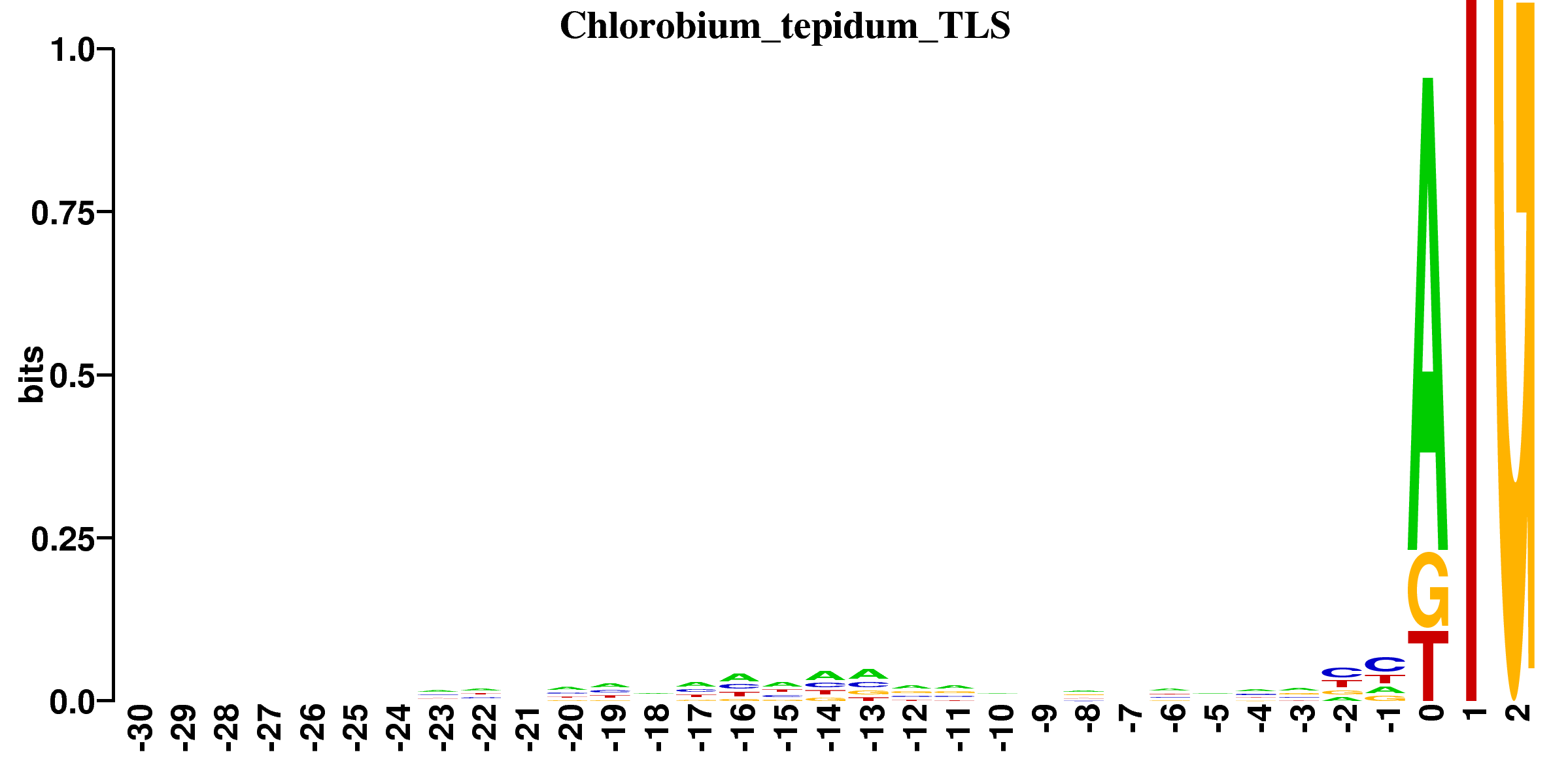


| genome % GC | start codon upstream region % GC | difference %GC | genome size [ Mb] |
| --- | --- | --- | --- |
| 56,5 | 46,1 | 10,4 | 2,2 |


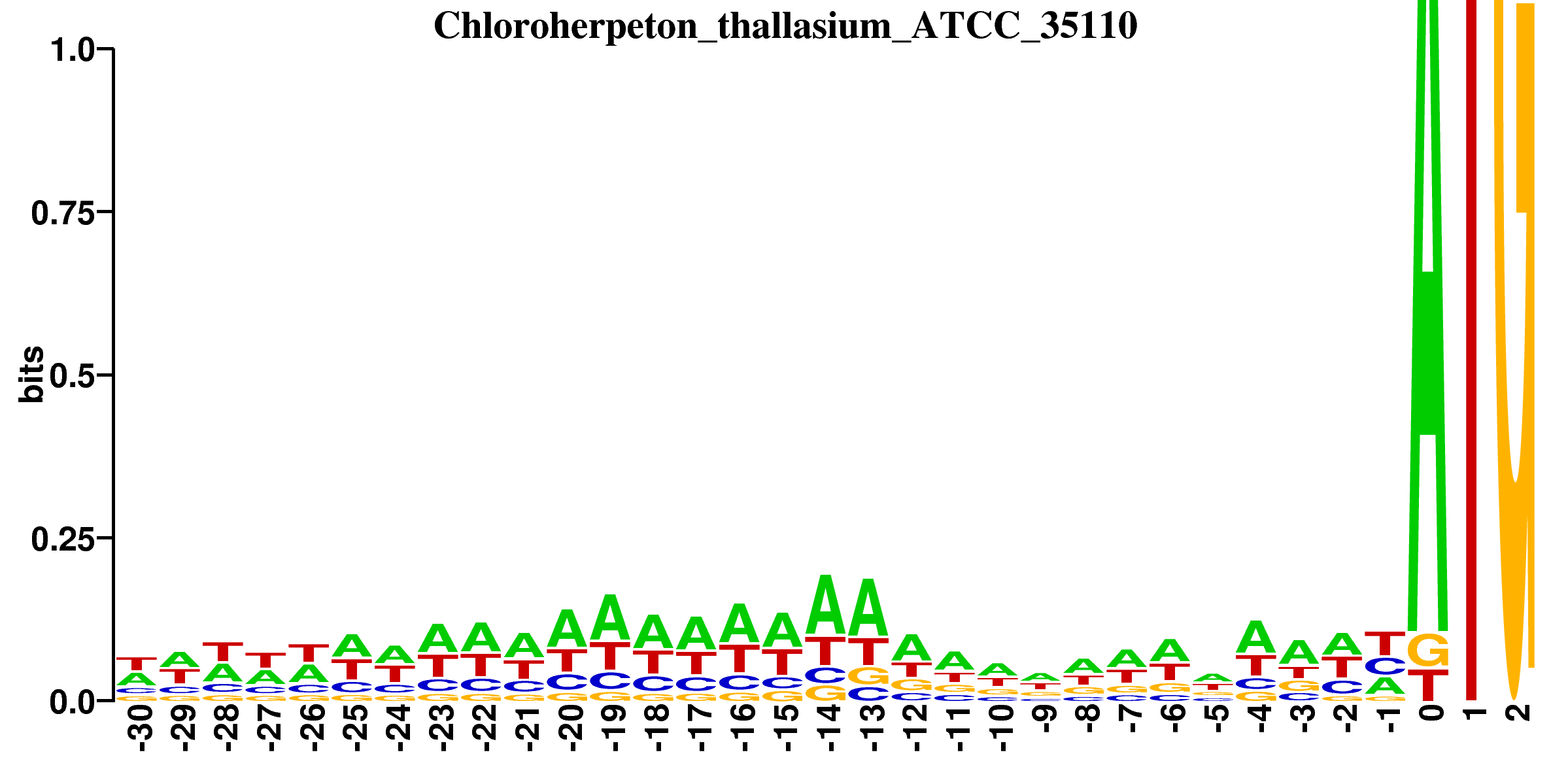


| genome % GC | start codon upstream region % GC | difference %GC | genome size [ Mb] |
| --- | --- | --- | --- |
| 45 | 32,6 | 12,4 | 3,3 |


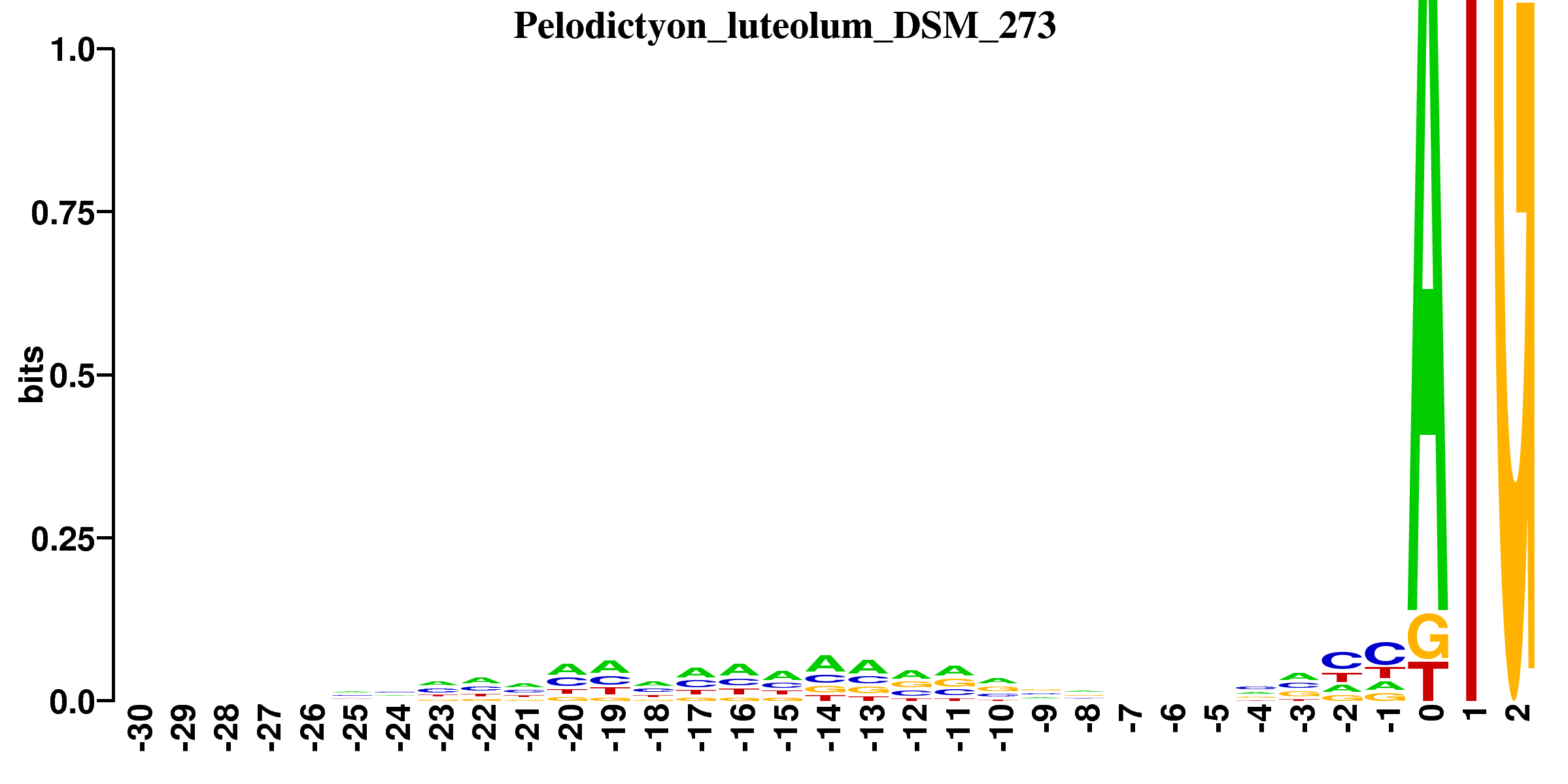


| genome % GC | start codon upstream region % GC | difference %GC | genome size [ Mb] |
| --- | --- | --- | --- |
| 57,3 | 49 | 8,3 | 2,4 |


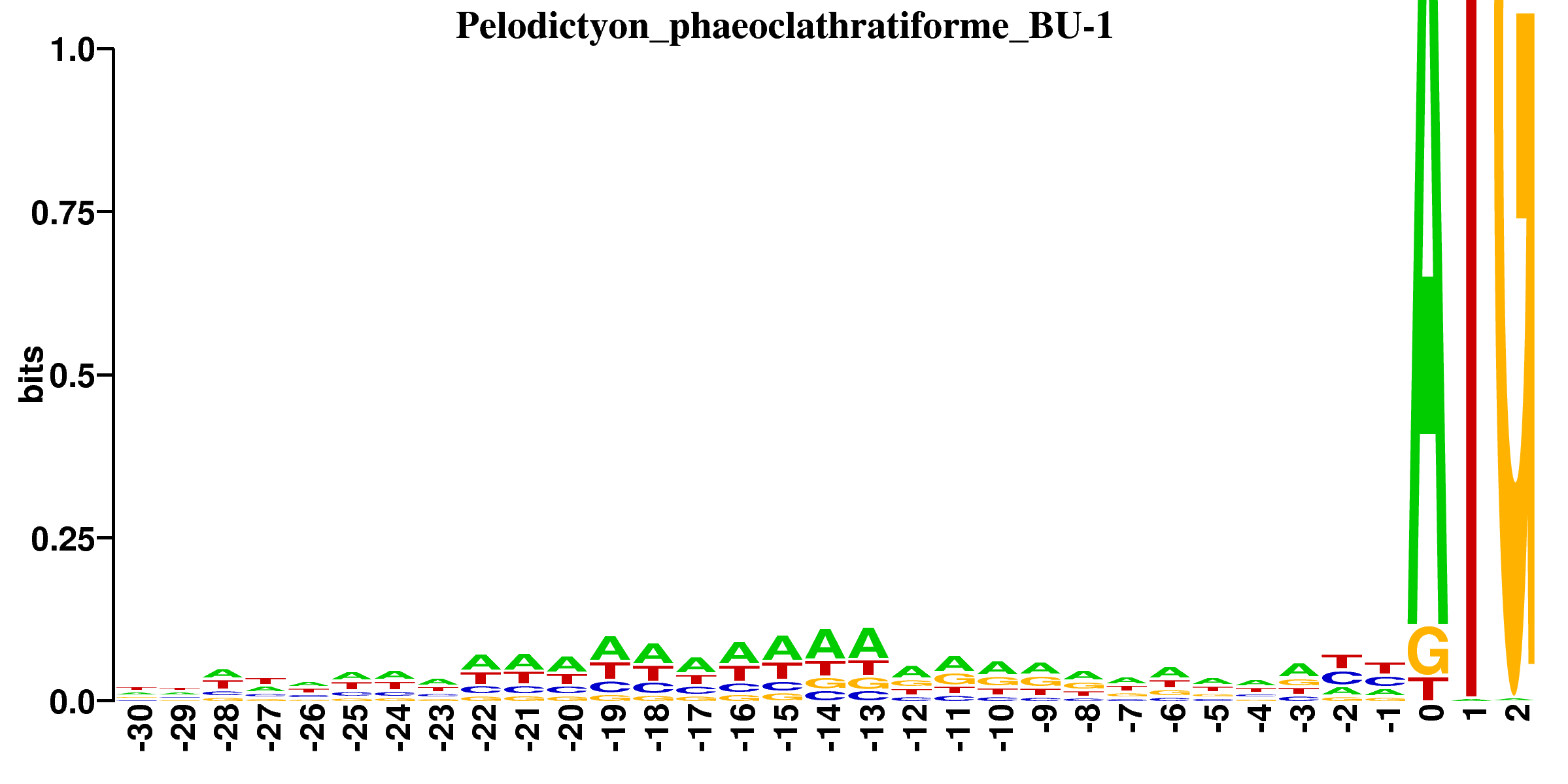


| genome % GC | start codon upstream region % GC | difference %GC | genome size [ Mb] |
| --- | --- | --- | --- |
| 48,1 | 38,4 | 9,7 | 3 |


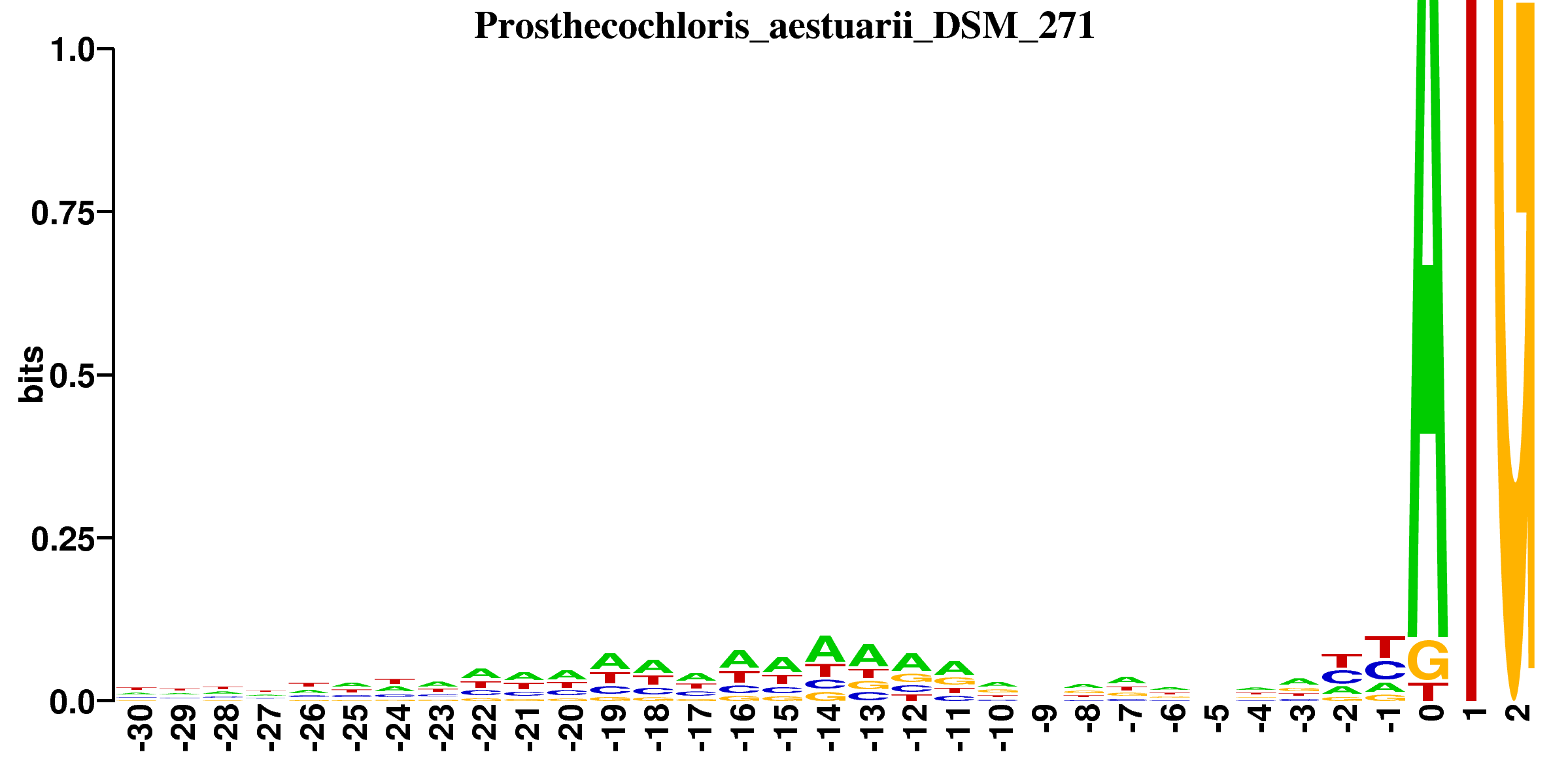


| genome % GC | start codon upstream region % GC | difference %GC | genome size [ Mb] |
| --- | --- | --- | --- |
| 50,1 | 40,5 | 9,6 | 2,6 |
