# Supplementary material for: Inability of Prevotella bryantii to Form a Functional Shine-Dalgarno Interaction Reflects Unique Evolution of Ribosome Binding Sites in Bacteroidetes
Source: PLoS One. 2011 Aug 12;6(8):e22914. doi: 10.1371/journal.pone.0022914 (PMC3155529; doi:10.1371/journal.pone.0022914)
Supplement: Figure S6 — Sequence logos of start codon upstream regions of Chloroflexi . (DOC) [file pone.0022914.s006.doc]

***CHLOROFLEXI***

**
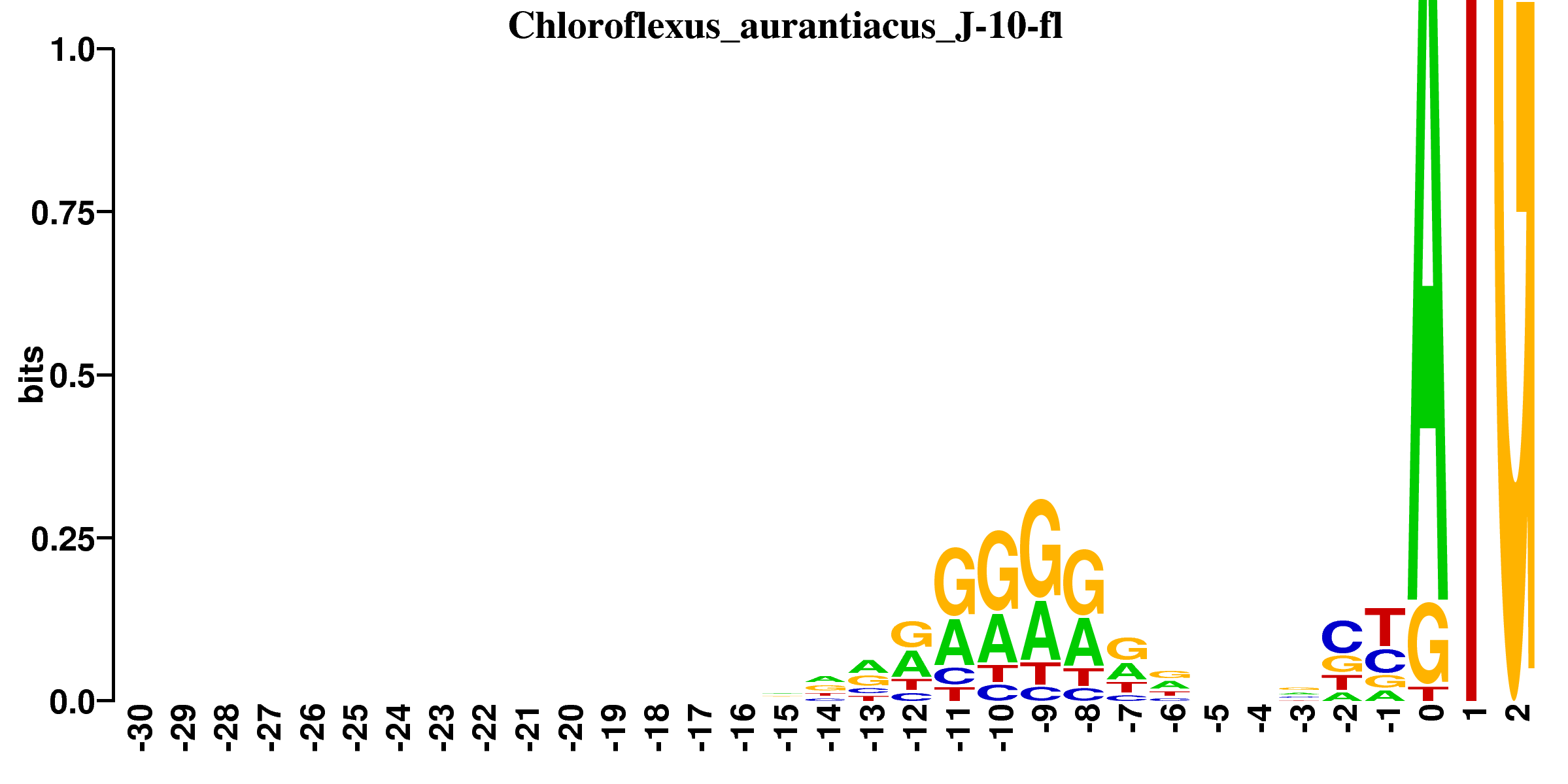
**

| genome % GC | start codon upstream region % GC | difference %GC | genome size [ Mb] |
| --- | --- | --- | --- |
| 56,7 | 50,6 | 6,1 | 5,3 |

**
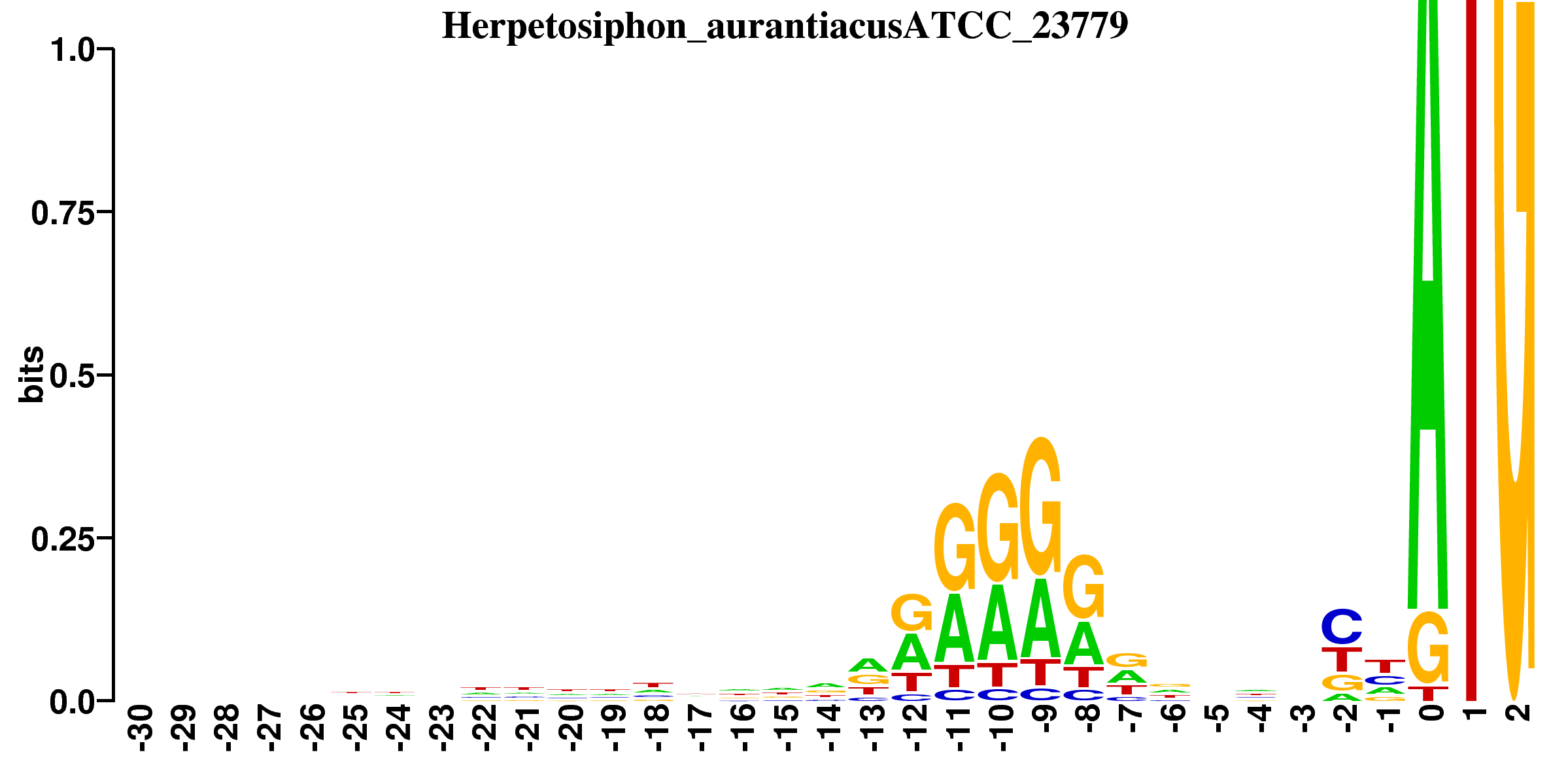
**

| genome % GC | start codon upstream region % GC | difference %GC | genome size [ Mb] |
| --- | --- | --- | --- |
| 50,9 | 46,1 | 4,8 | 6,7 |

**
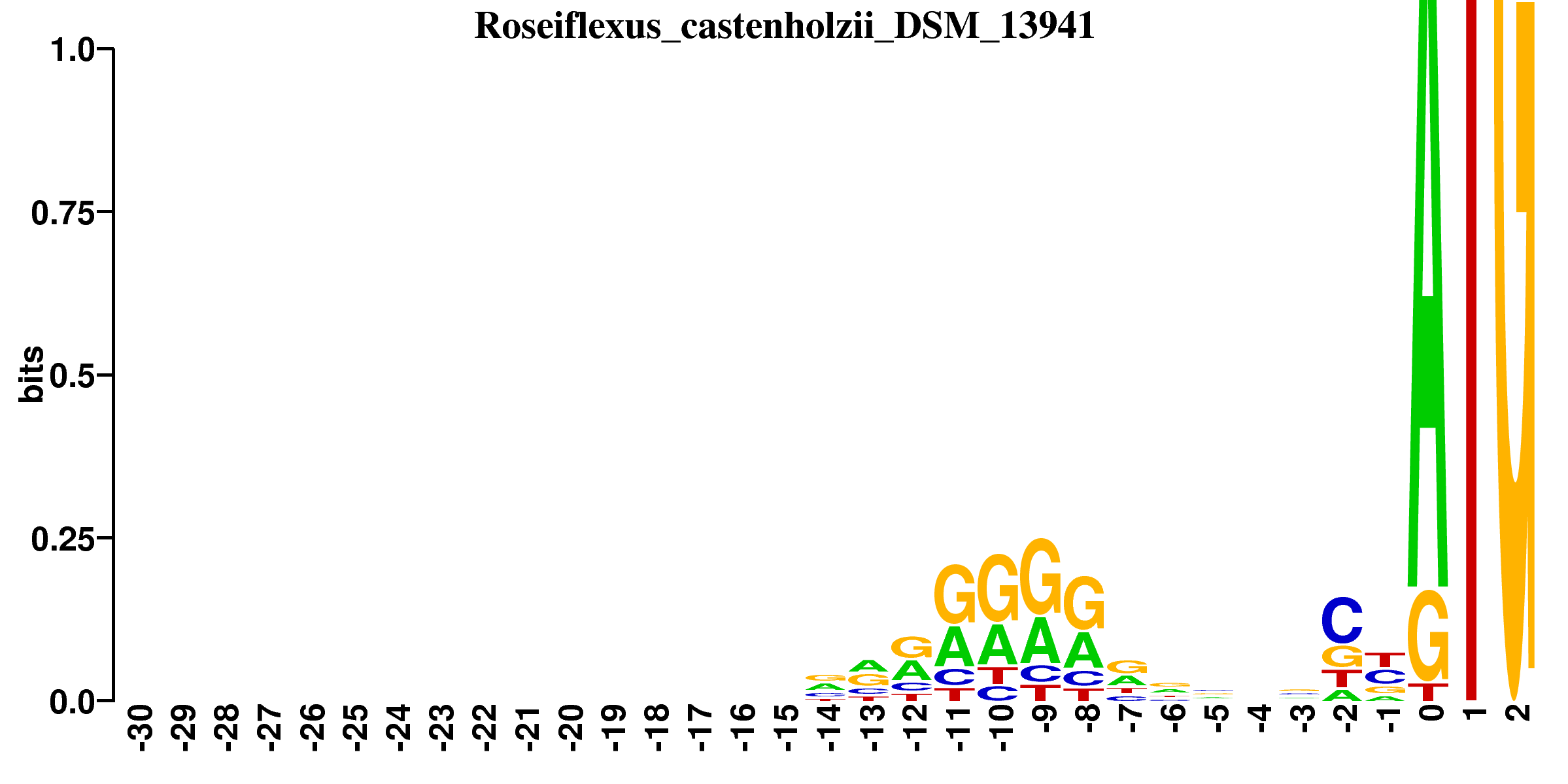
**

| genome % GC | start codon upstream region % GC | difference %GC | genome size [ Mb] |
| --- | --- | --- | --- |
| 60,7 | 54,5 | 6,2 | 5,7 |

**
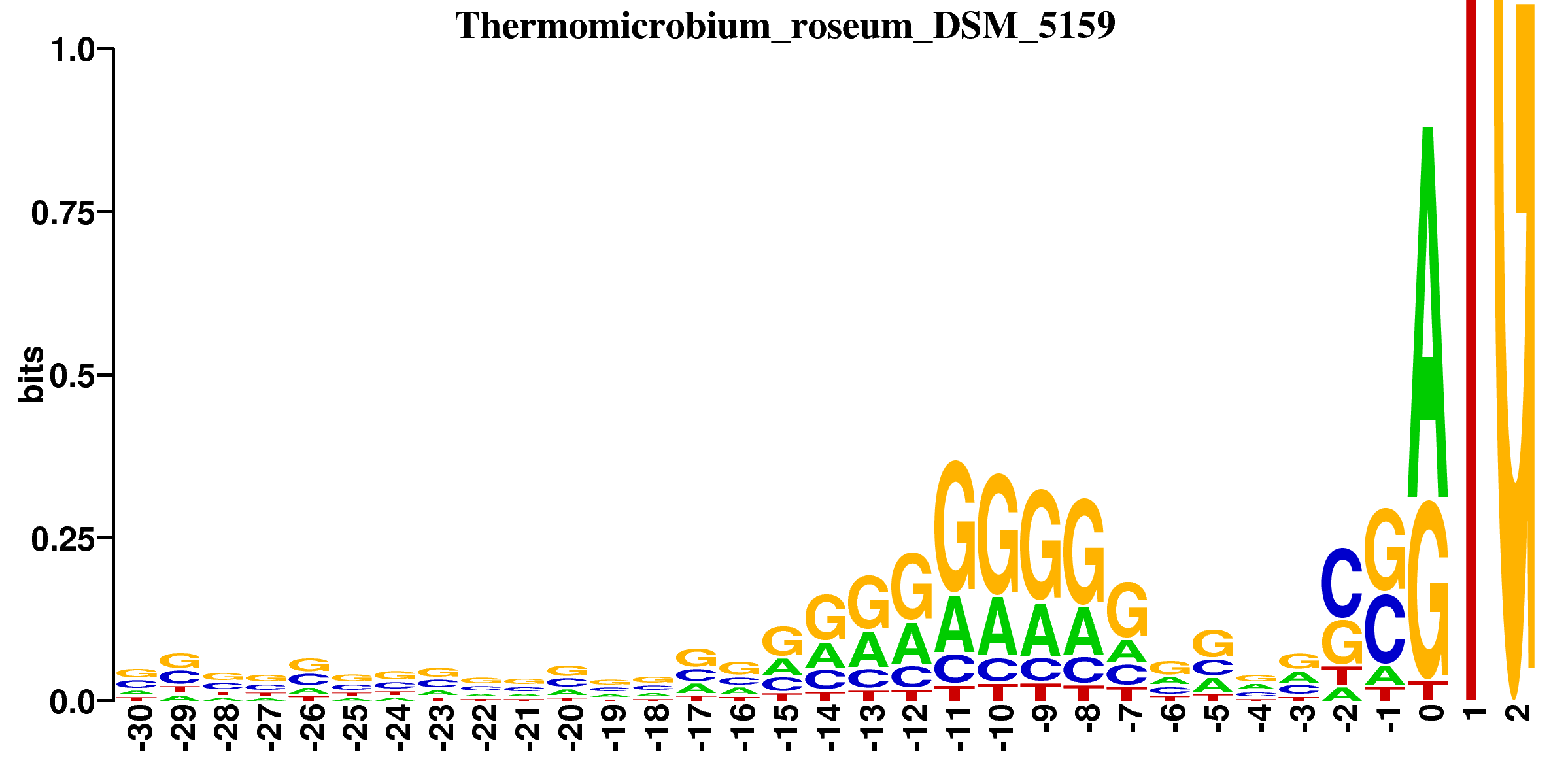
**

| shromosome % GC | start codon upstream region % GC | difference %GC | genome size [ Mb] |
| --- | --- | --- | --- |
| 63,7 | 63,9 | -0,2 | 2,9 |
