# Supplementary material for: Inability of Prevotella bryantii to Form a Functional Shine-Dalgarno Interaction Reflects Unique Evolution of Ribosome Binding Sites in Bacteroidetes
Source: PLoS One. 2011 Aug 12;6(8):e22914. doi: 10.1371/journal.pone.0022914 (PMC3155529; doi:10.1371/journal.pone.0022914)
Supplement: Figure S7 — Sequence logos of start codon upstream regions of Cyanobacteria . (DOC) [file pone.0022914.s007.doc]

***CYANOBACTERIA***

**
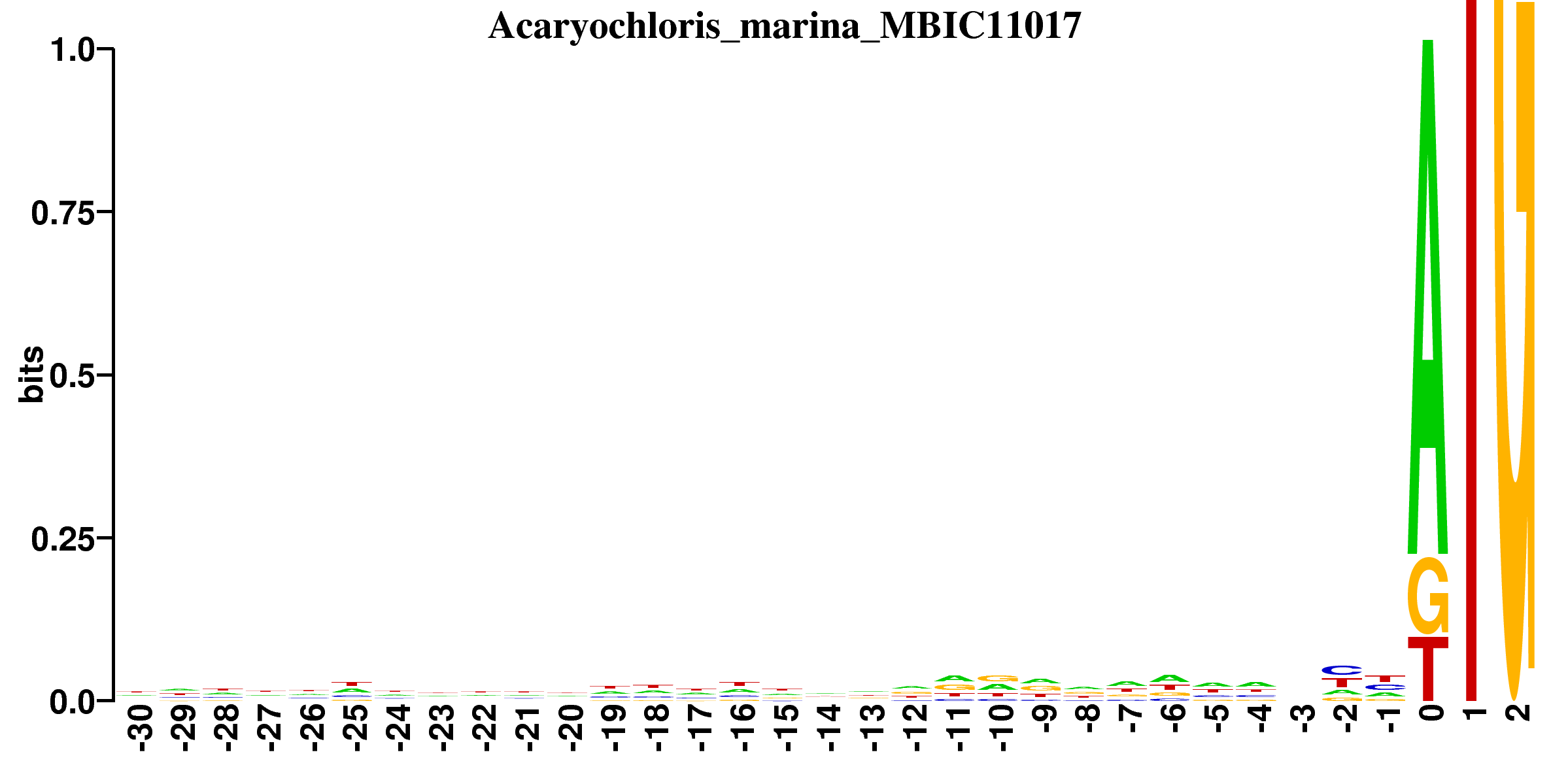
**

| genome % GC | start codon upstream region % GC | difference %GC | genome size [ Mb] |
| --- | --- | --- | --- |
| 47 | 42,7 | 4,3 | 8,4 |

**
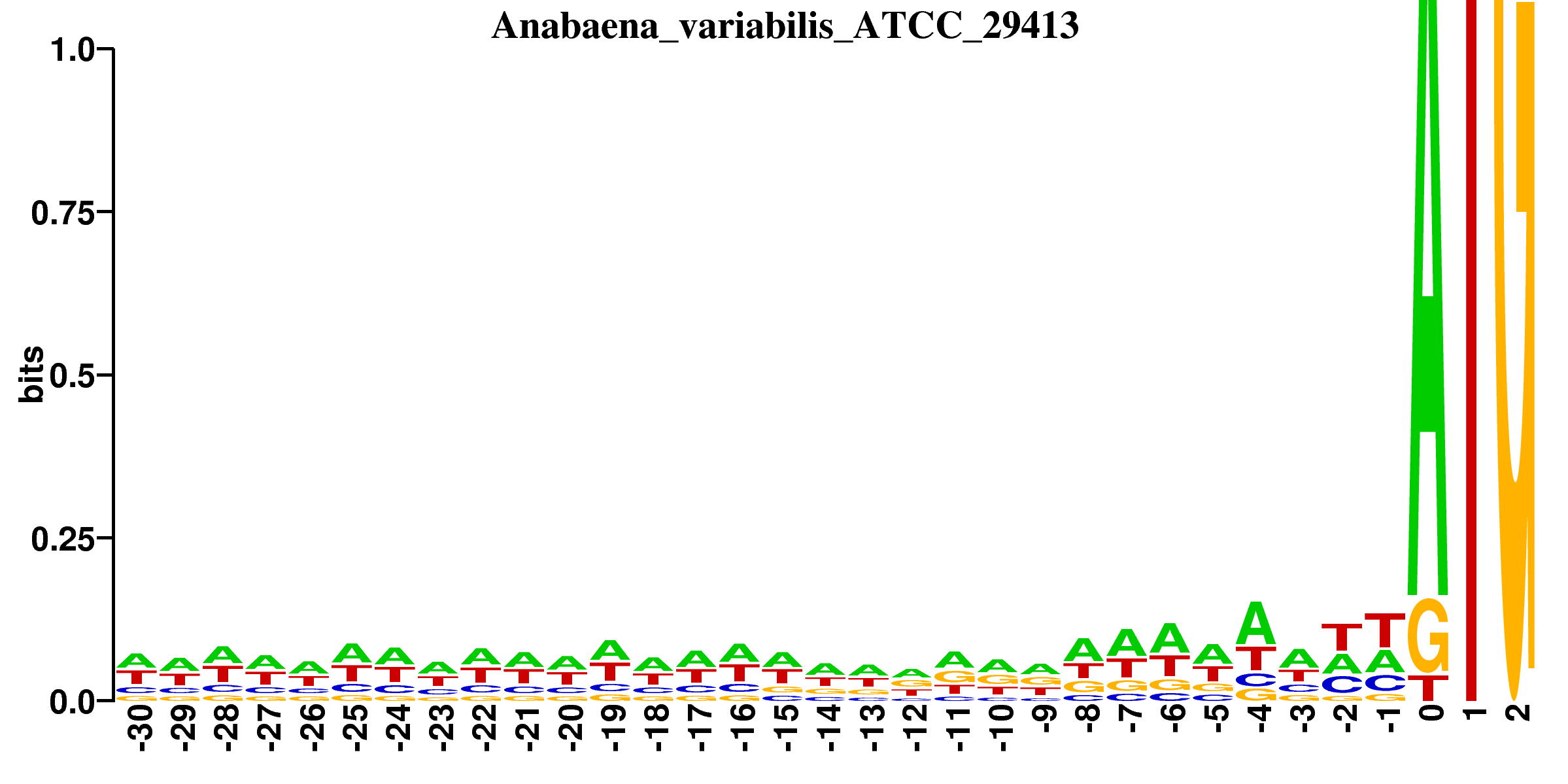
**

| genome % GC | start codon upstream region % GC | difference %GC | genome size [ Mb] |
| --- | --- | --- | --- |
| 41,4 | 34,7 | 6,7 | 7,1 |

**
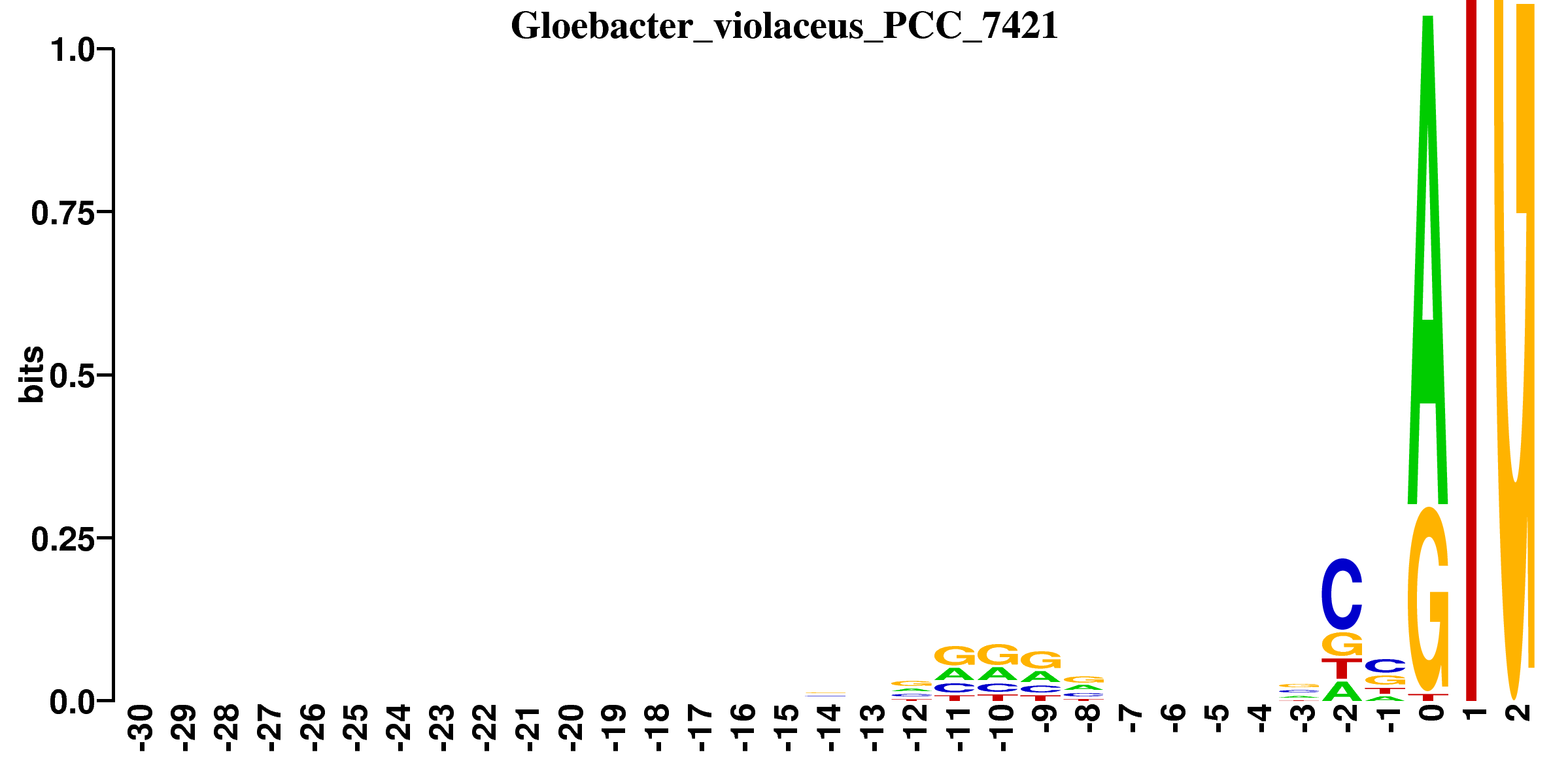
**

| genome % GC | start codon upstream region % GC | difference %GC | genome size [ Mb] |
| --- | --- | --- | --- |
| 62 | 55,8 | 6,2 | 4,7 |

**
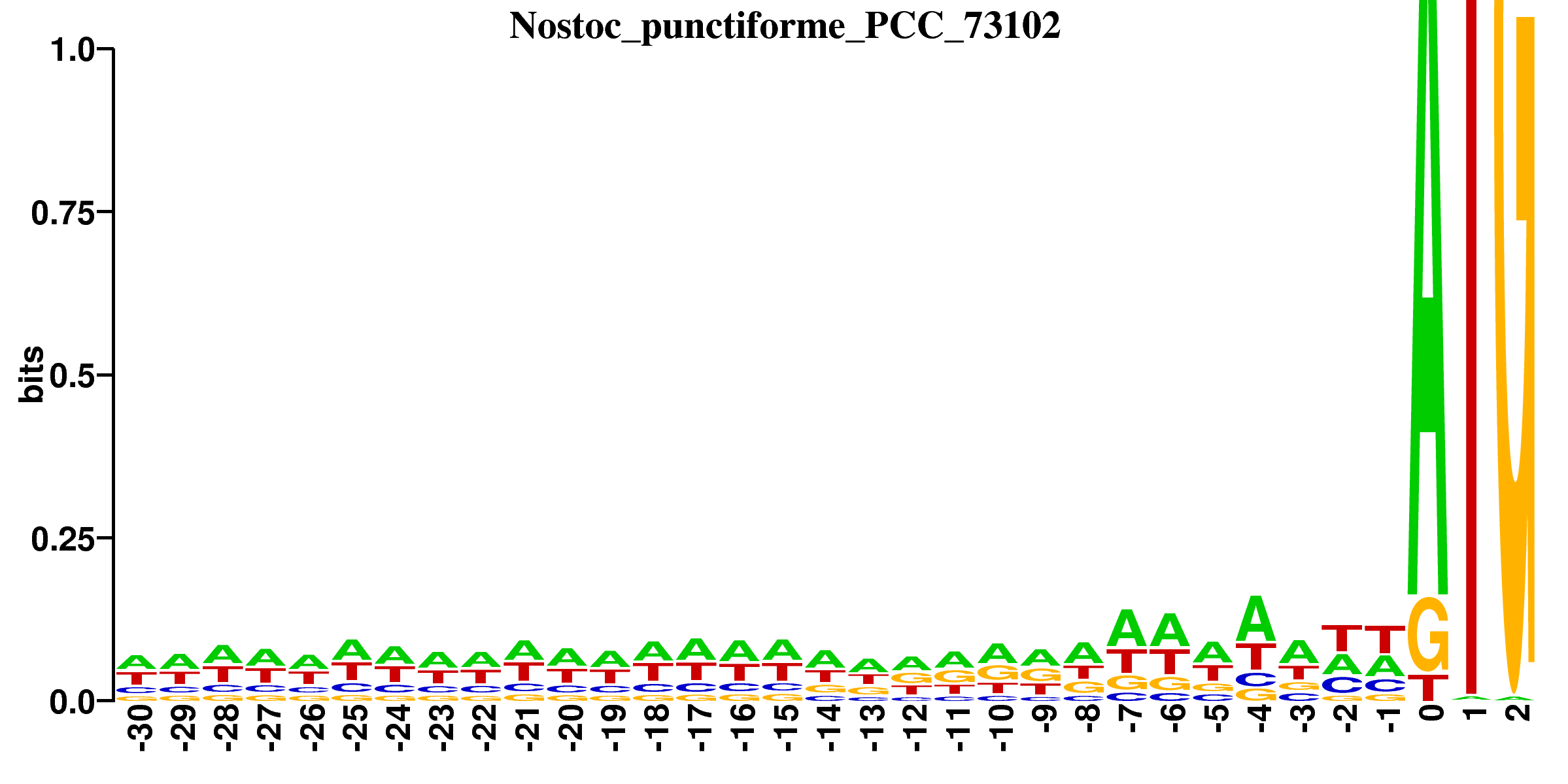
**

| genome % GC | start codon upstream region % GC | difference %GC | genome size [ Mb] |
| --- | --- | --- | --- |
| 41,4 | 34,2 | 7,2 | 9 |

**
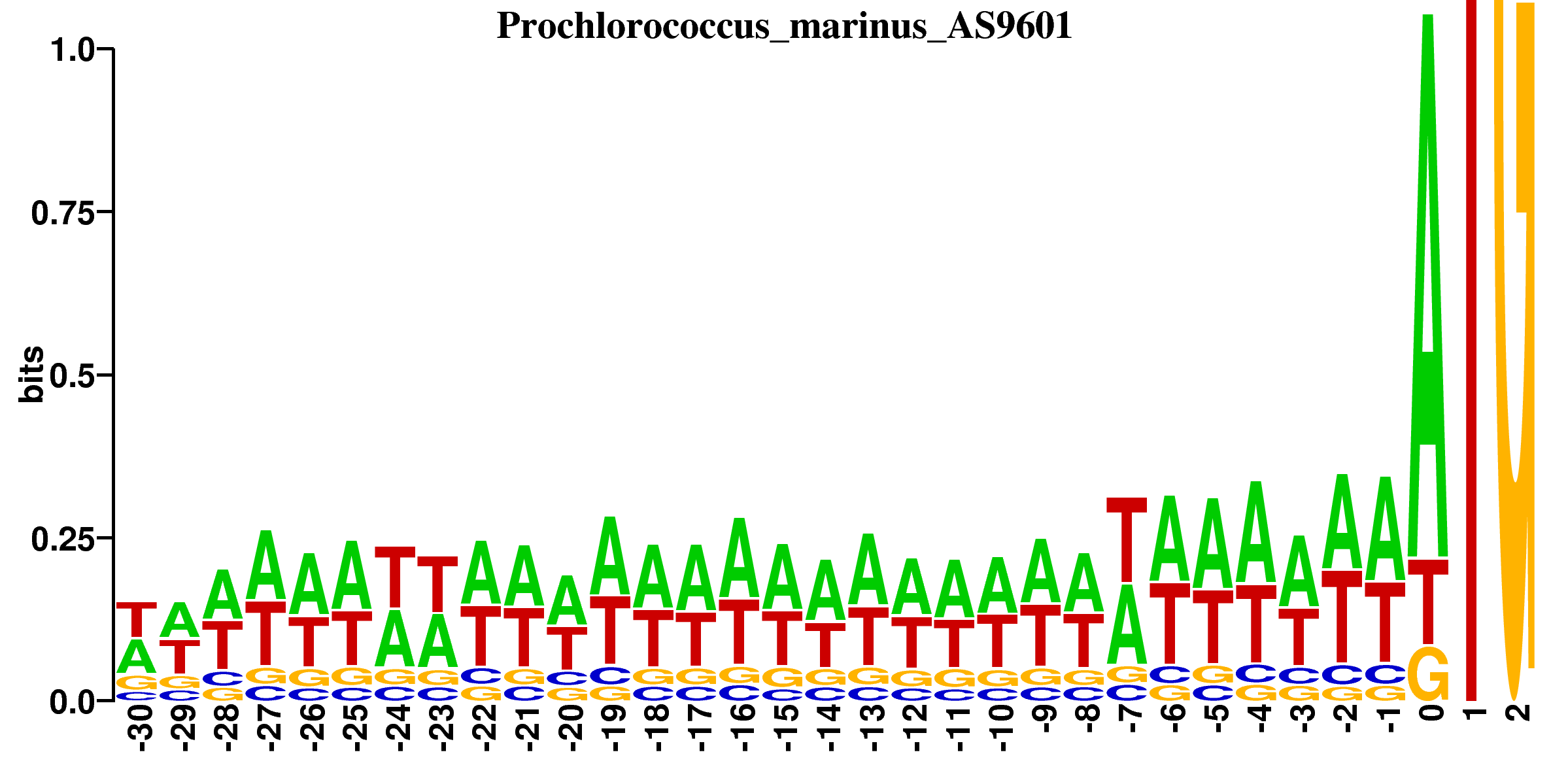
**

| genome % GC | start codon upstream region % GC | difference %GC | genome size [ Mb] |
| --- | --- | --- | --- |
| 31,3 | 21,6 | 9,7 | 1,7 |

**
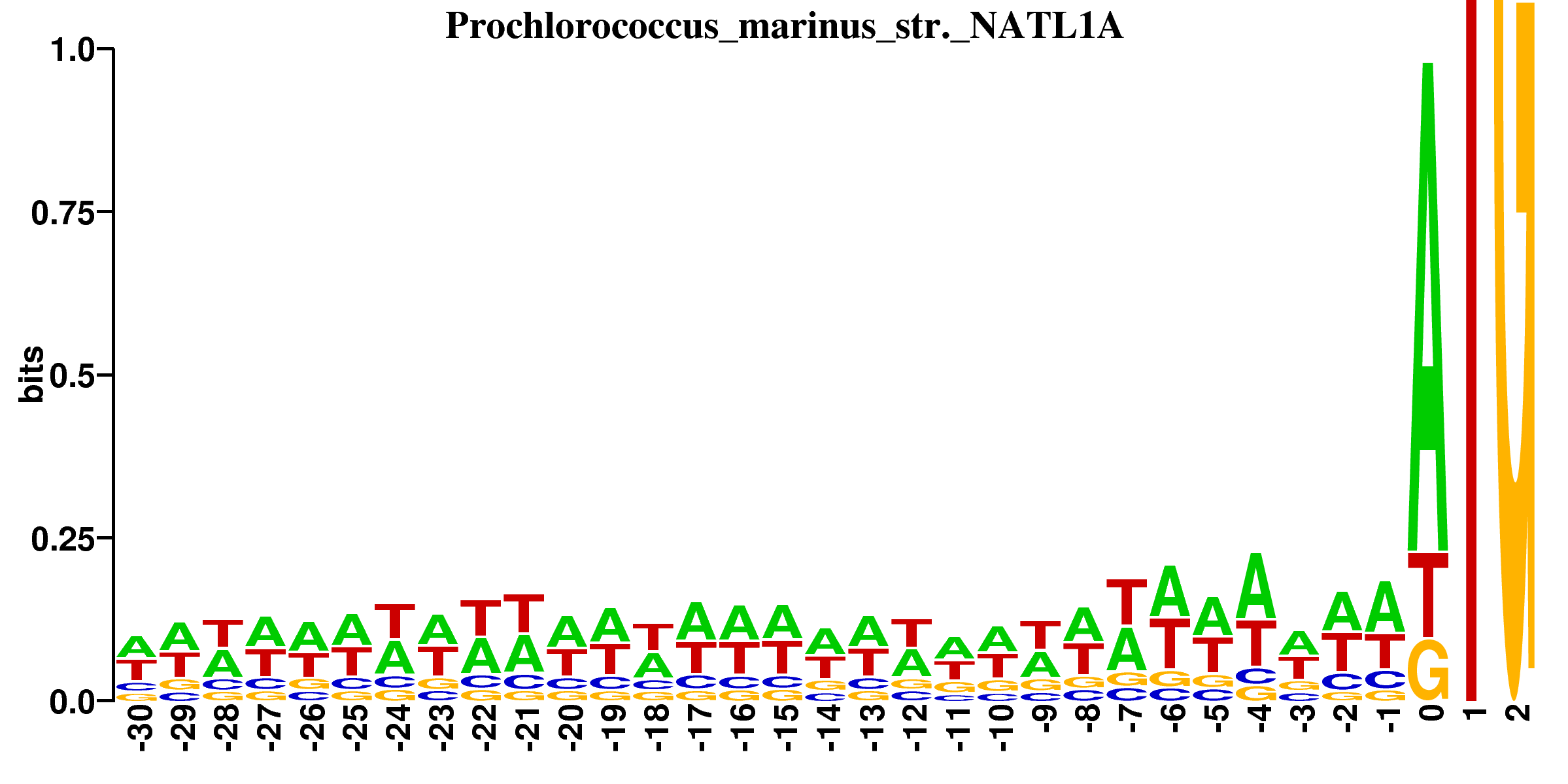
**

| genome % GC | start codon upstream region % GC | difference %GC | genome size [ Mb] |
| --- | --- | --- | --- |
| 35 | 28,2 | 6,8 | 1,9 |

**
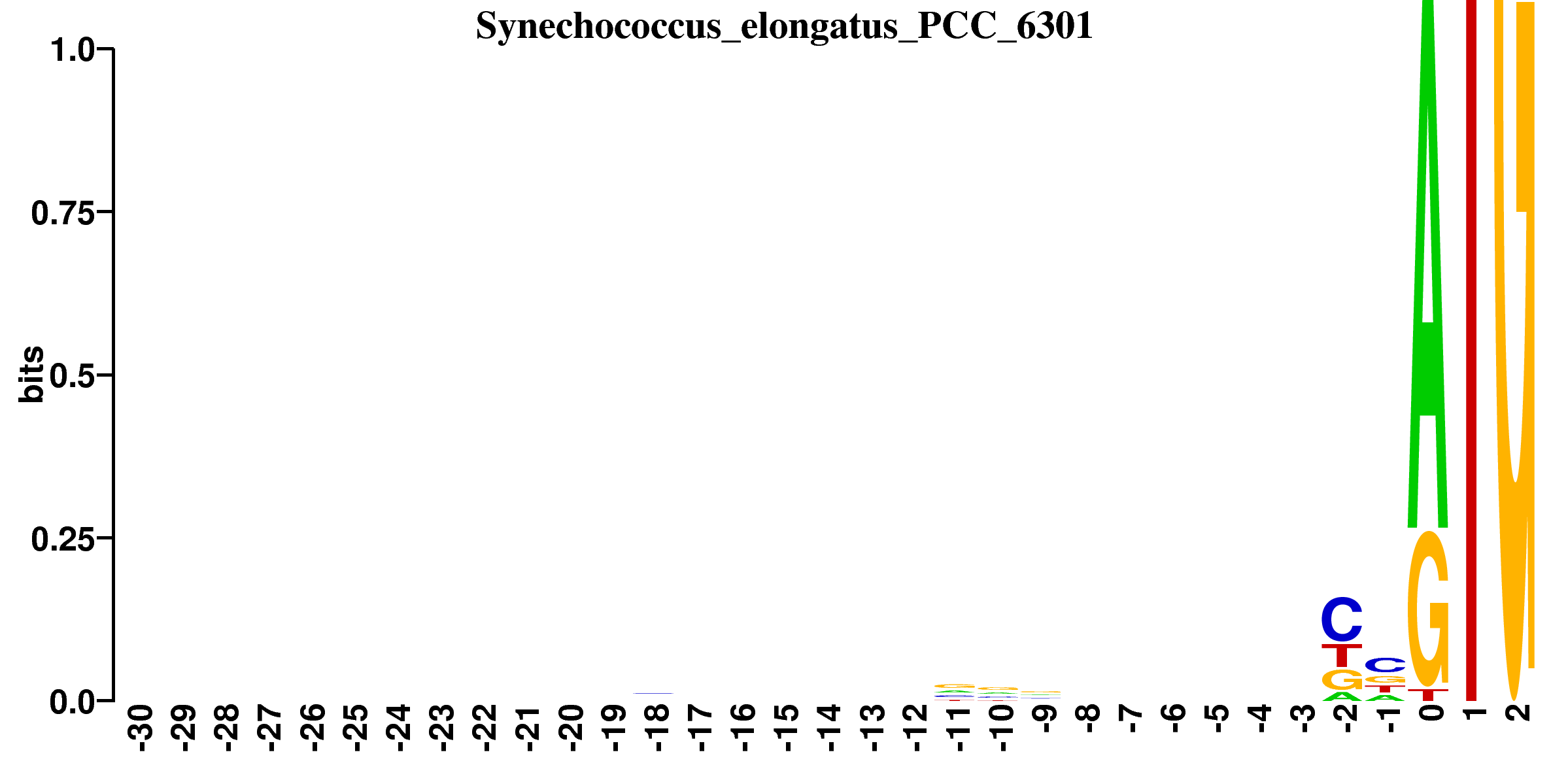
**

| genome % GC | start codon upstream region % GC | difference %GC | genome size [ Mb] |
| --- | --- | --- | --- |
| 55,5 | 53,3 | 2,2 | 2,7 |

**
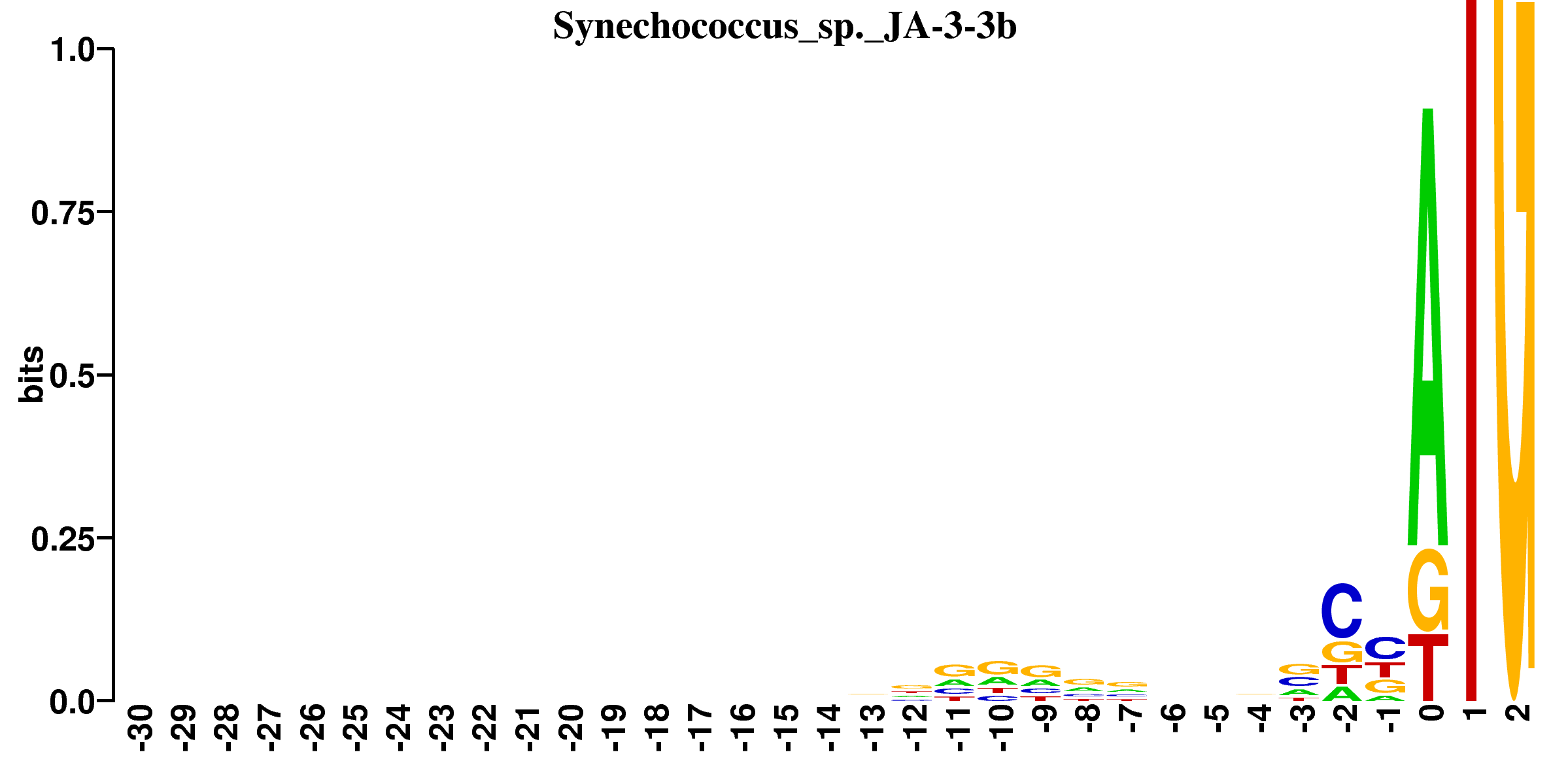
**

| genome % GC | start codon upstream region % GC | difference %GC | genome size [ Mb] |
| --- | --- | --- | --- |
| 60,2 | 55 | 5,2 | 2,9 |
