# Supplementary figures and images for: Inability of Prevotella bryantii to Form a Functional Shine-Dalgarno Interaction Reflects Unique Evolution of Ribosome Binding Sites in Bacteroidetes
Source: PLoS One. 2011 Aug 12;6(8):e22914. doi: 10.1371/journal.pone.0022914 (PMC3155529; doi:10.1371/journal.pone.0022914)

***FIBROBACTERES***

**
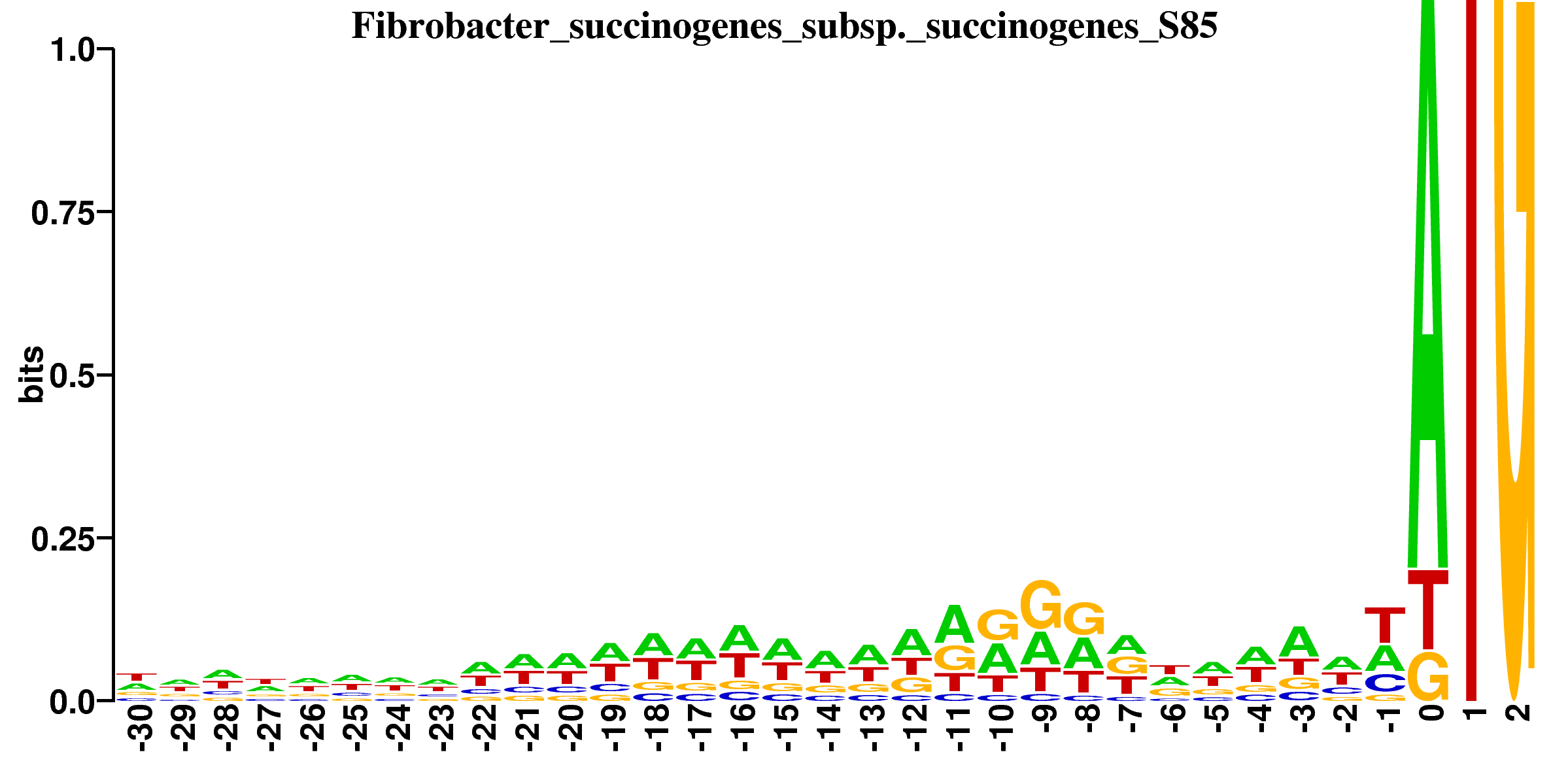
**

| genome % GC | start codon upstream region % GC | difference %GC | genome size [ Mb] |
| --- | --- | --- | --- |
| 48 | 36,5 | 11,5 | 3,8 |

Supplement: Figure S8 — Sequence logos of start codon upstream regions of Fibrobacteres . (DOC) [file pone.0022914.s008.doc]
