# Supplementary material for: Inability of Prevotella bryantii to Form a Functional Shine-Dalgarno Interaction Reflects Unique Evolution of Ribosome Binding Sites in Bacteroidetes
Source: PLoS One. 2011 Aug 12;6(8):e22914. doi: 10.1371/journal.pone.0022914 (PMC3155529; doi:10.1371/journal.pone.0022914)
Supplement: Figure S9 — Sequence logos of start codon upstream regions of Firmicutes . (DOC) [file pone.0022914.s009.doc]

***FIRMICUTES***

**
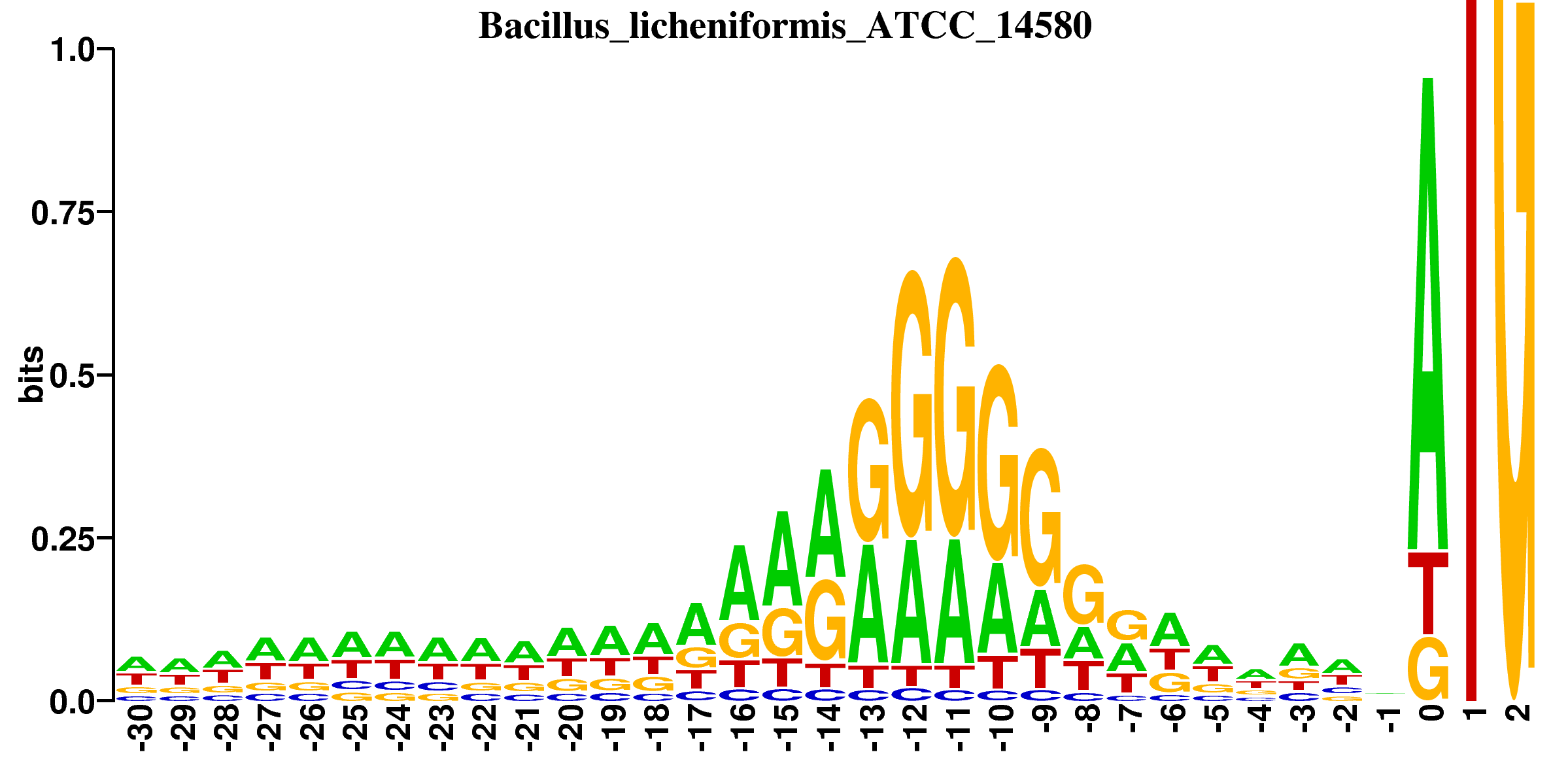
**

| genome % GC | start codon upstream region % GC | difference %GC | genome size [ Mb] |
| --- | --- | --- | --- |
| 46,2 | 40,1 | 6,1 | 4,2 |

**
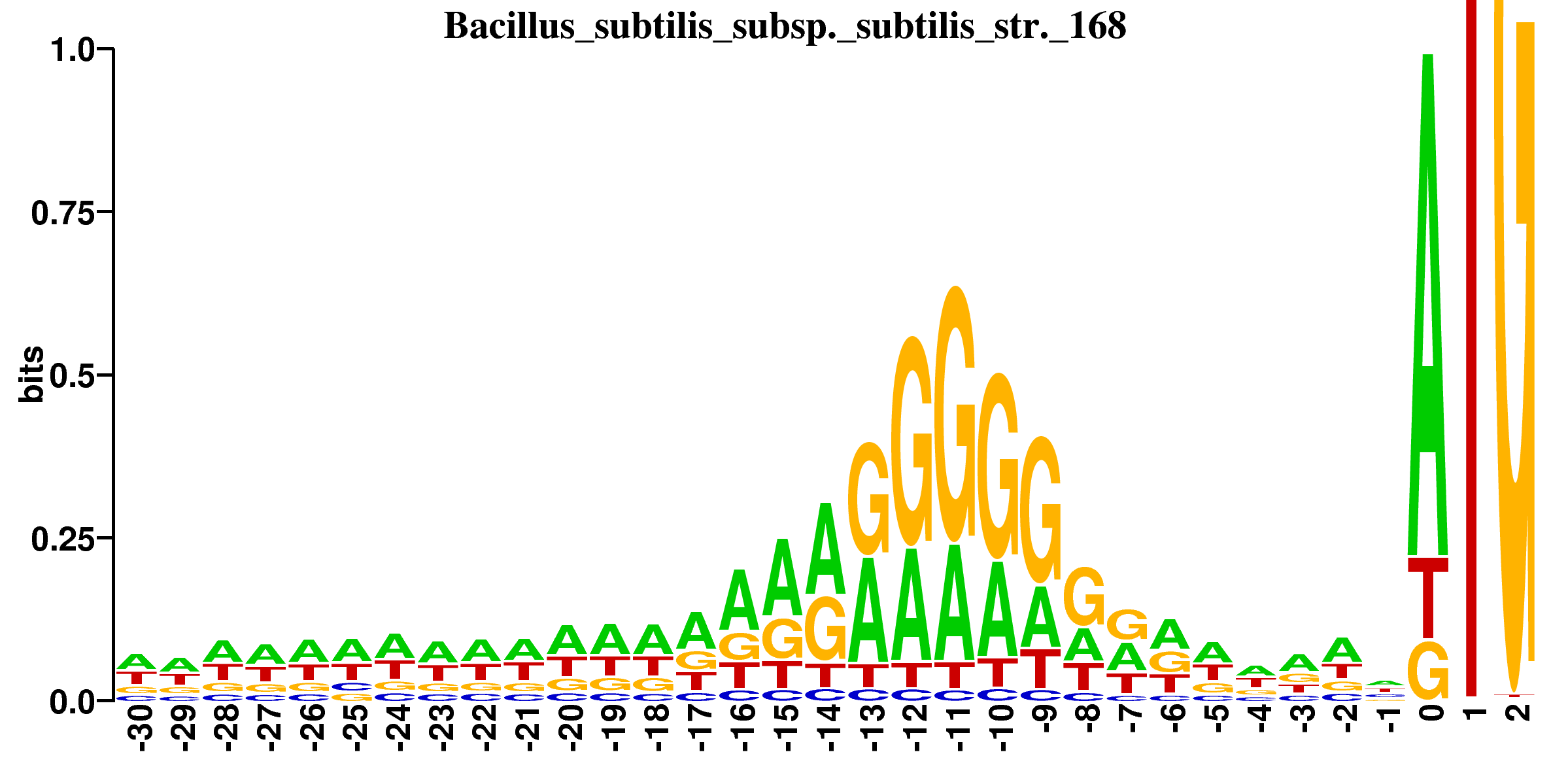
**

| genome % GC | start codon upstream region % GC | difference %GC | genome size [ Mb] |
| --- | --- | --- | --- |
| 43,5 | 39,4 | 4,1 | 4,2 |

**
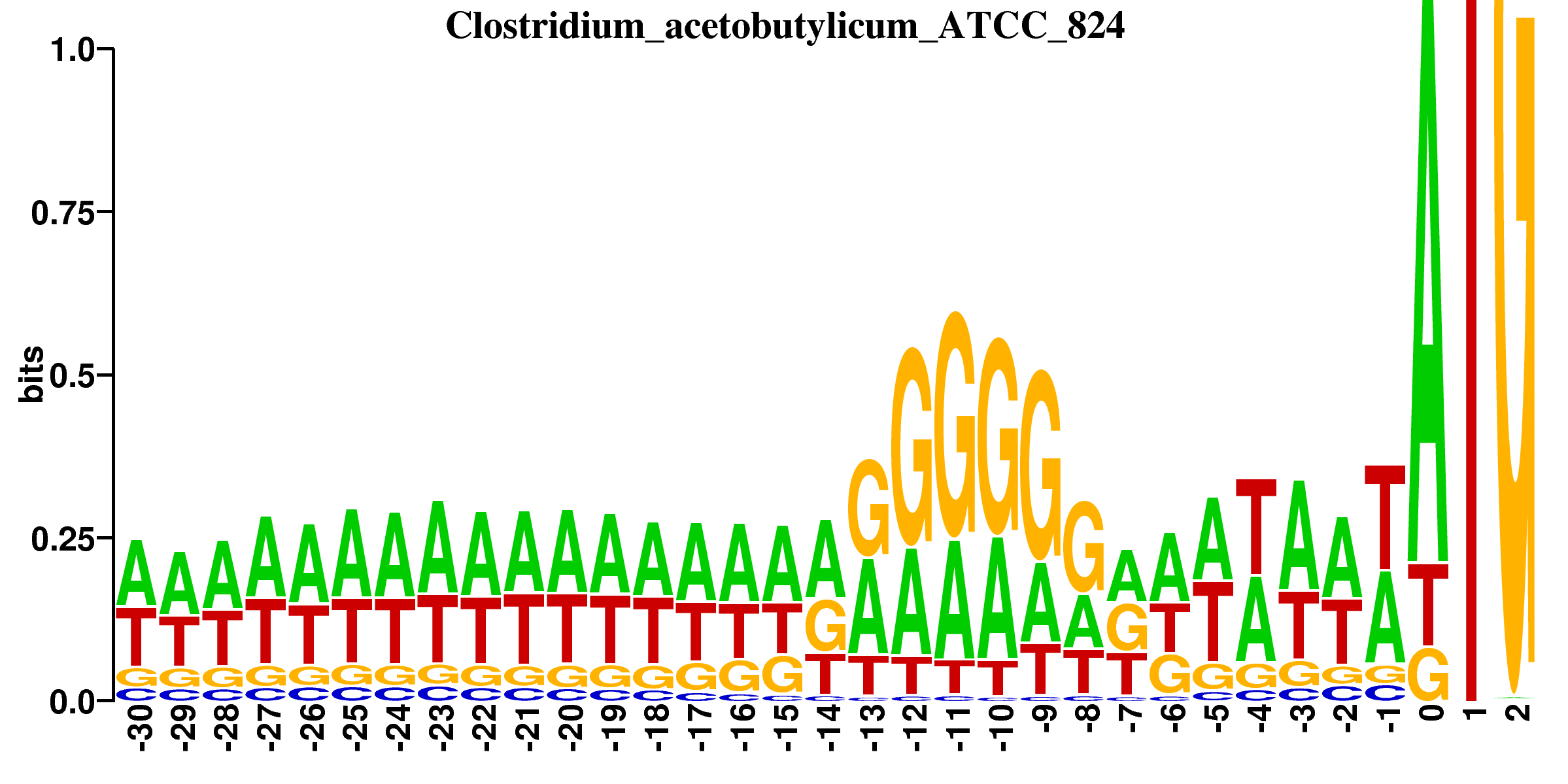
**

| genome % GC | start codon upstream region % GC | difference %GC | genome size [ Mb] |
| --- | --- | --- | --- |
| 30,9 | 28,7 | 2,2 | 4,1 |

**
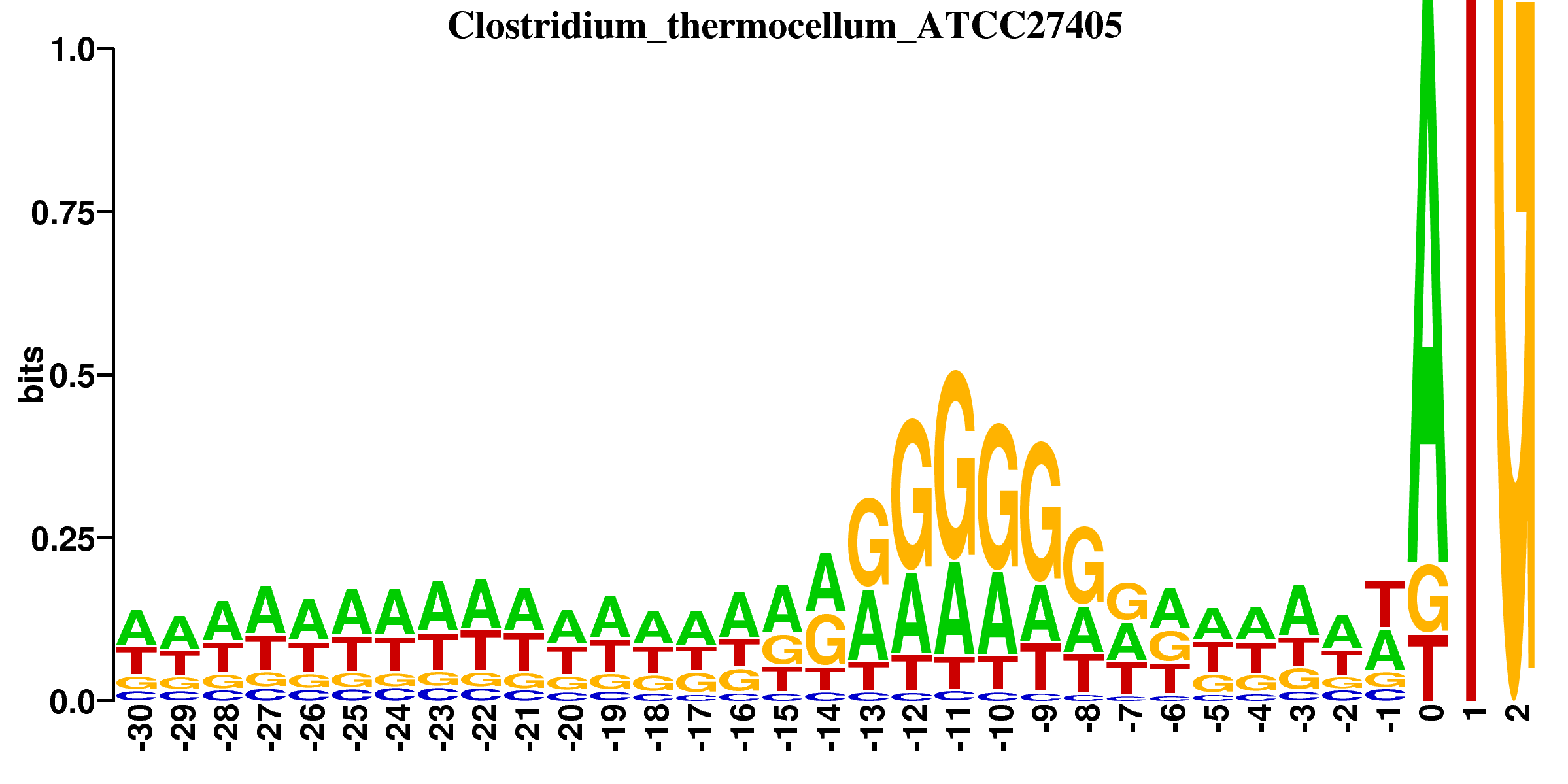
**

| genome % GC | start codon upstream region % GC | difference %GC | genome size [ Mb] |
| --- | --- | --- | --- |
| 39 | 35,12 | 3,88 | 3,8 |

**
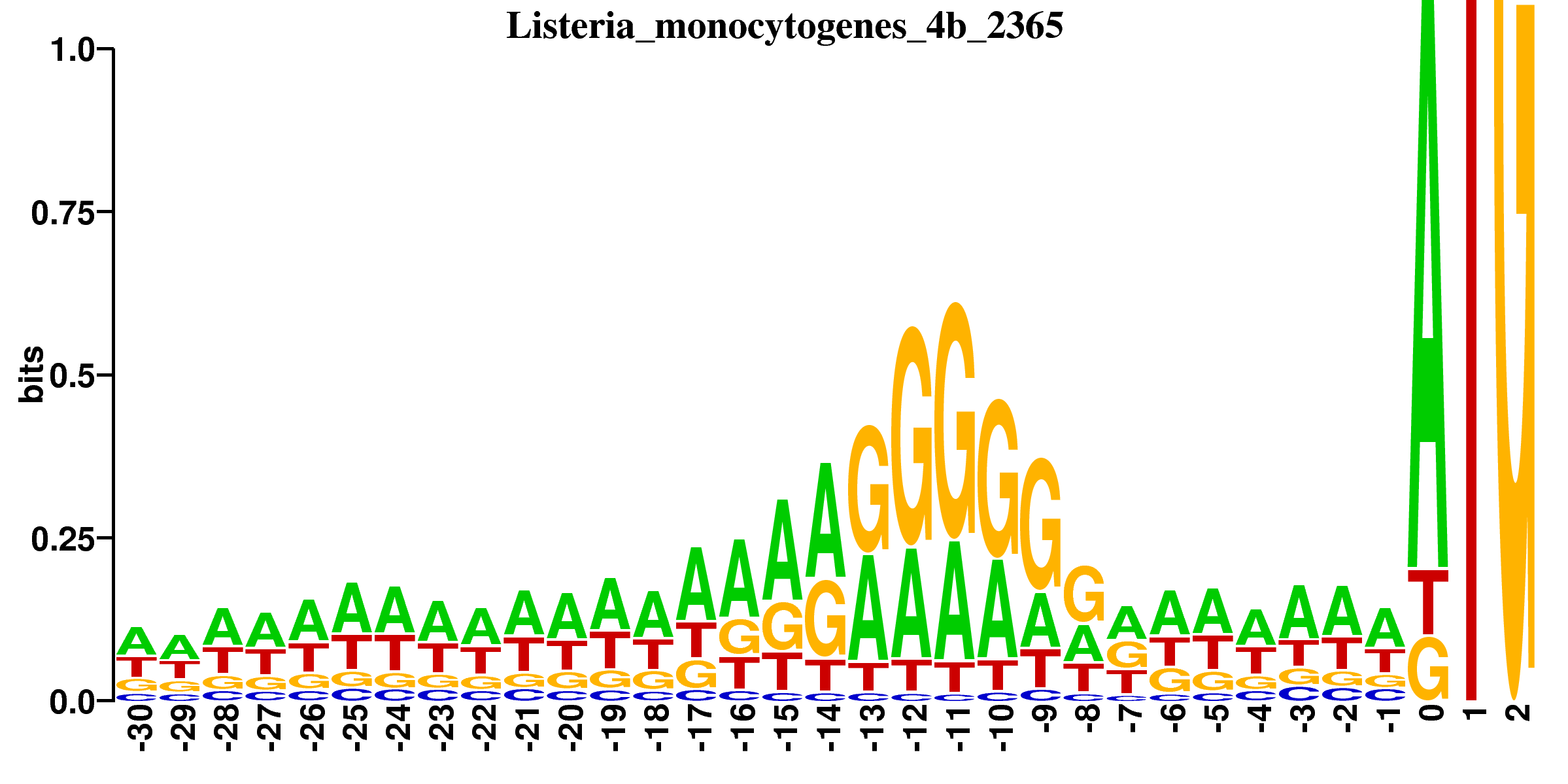
**

| genome % GC | start codon upstream region % GC | difference %GC | genome size [ Mb] |
| --- | --- | --- | --- |
| 38 | 35 | 3 | 2,9 |

**
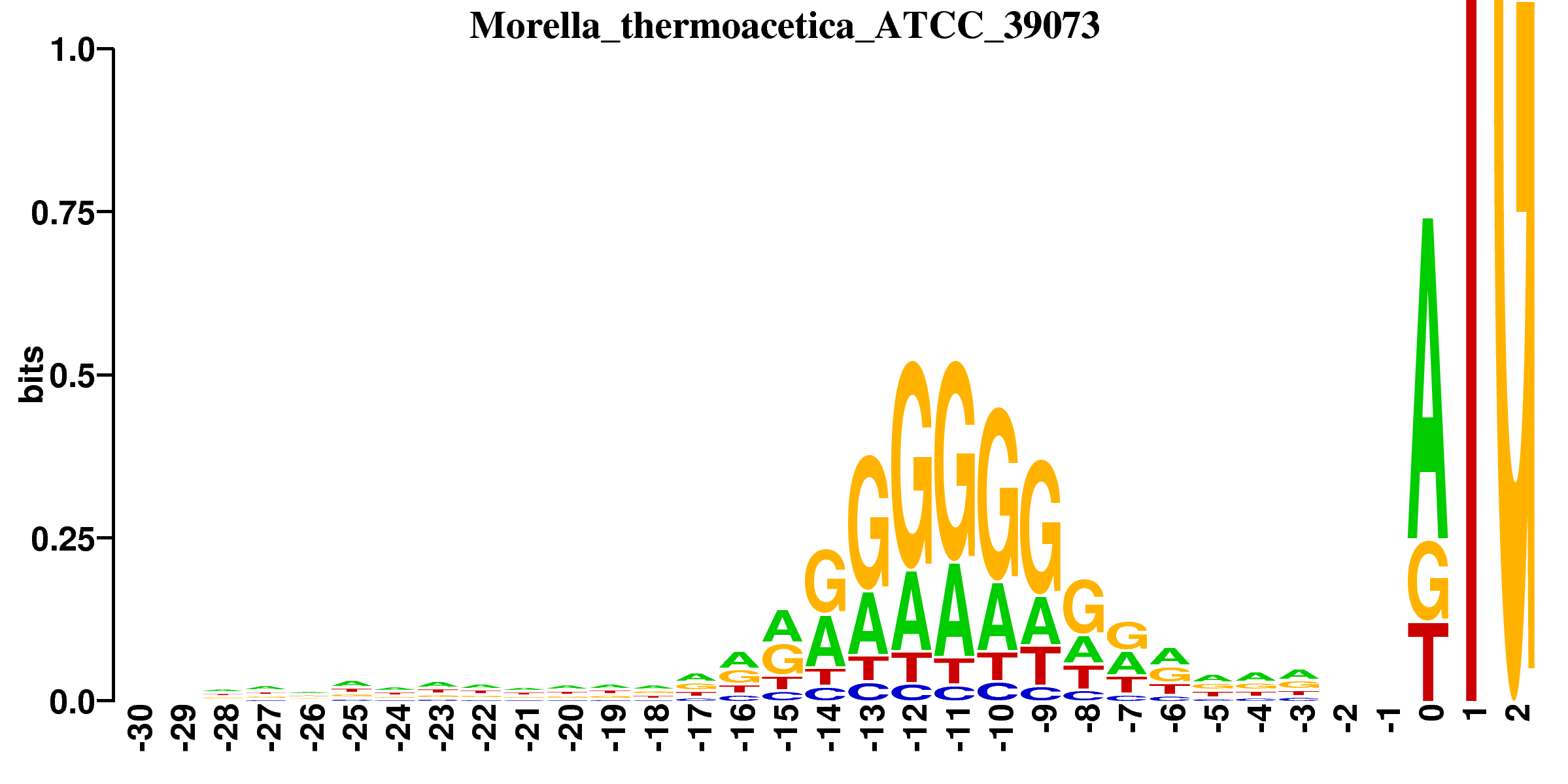
**

| genome % GC | start codon upstream region % GC | difference %GC | genome size [ Mb] |
| --- | --- | --- | --- |
| 55,8 | 47,8 | 8 | 2,6 |

**
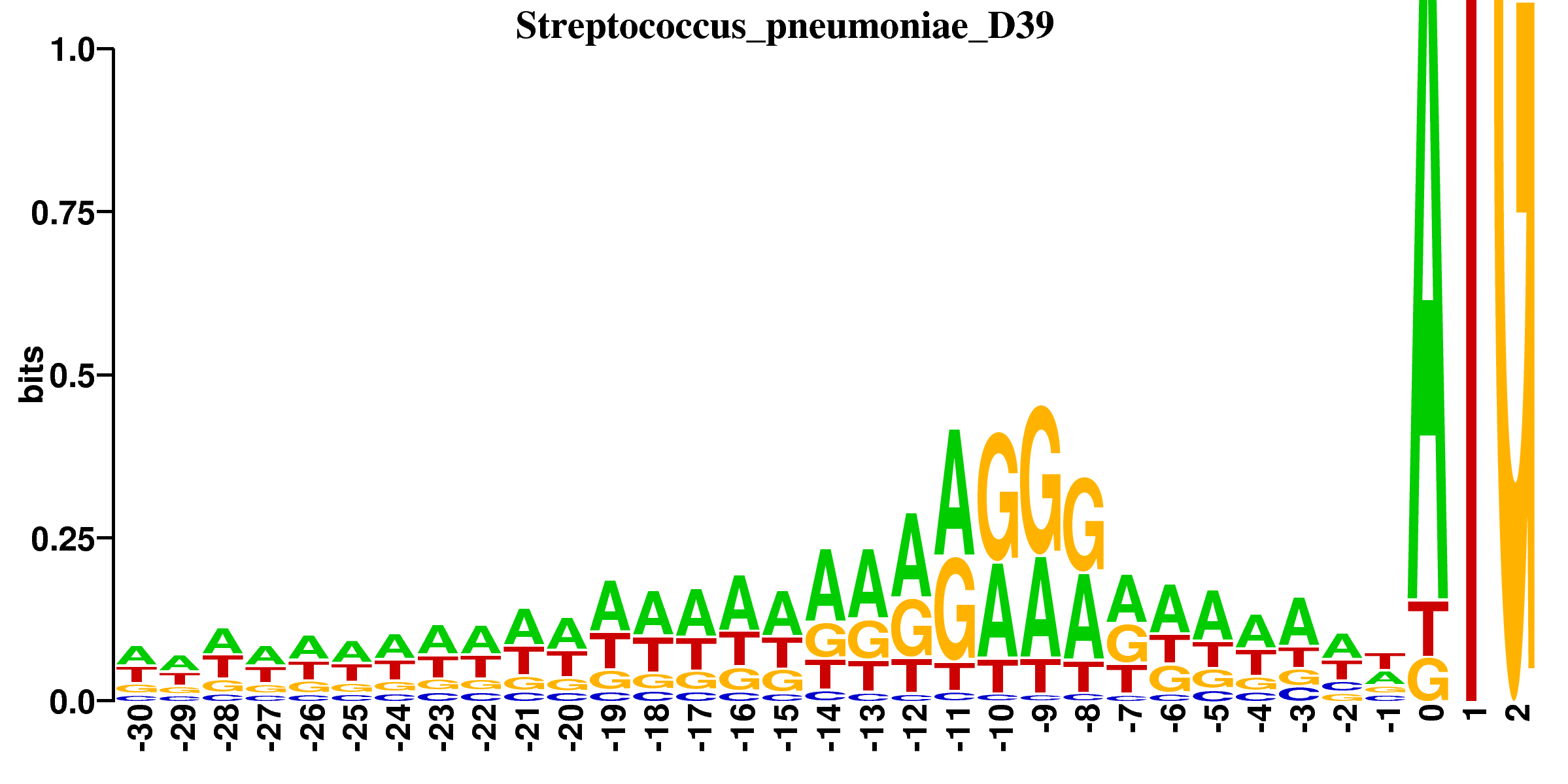
**

| genome % GC | start codon upstream region % GC | difference %GC | genome size [ Mb] |
| --- | --- | --- | --- |
| 39,7 | 33,4 | 6,3 | 2 |
