# Supplementary material for: Inability of Prevotella bryantii to Form a Functional Shine-Dalgarno Interaction Reflects Unique Evolution of Ribosome Binding Sites in Bacteroidetes
Source: PLoS One. 2011 Aug 12;6(8):e22914. doi: 10.1371/journal.pone.0022914 (PMC3155529; doi:10.1371/journal.pone.0022914)
Supplement: Figure S13 — Sequence logos of start codon upstream regions of Deinococcus-Thermus . (DOC) [file pone.0022914.s013.doc]

***DEINOCOCCUS THERMUS***

***
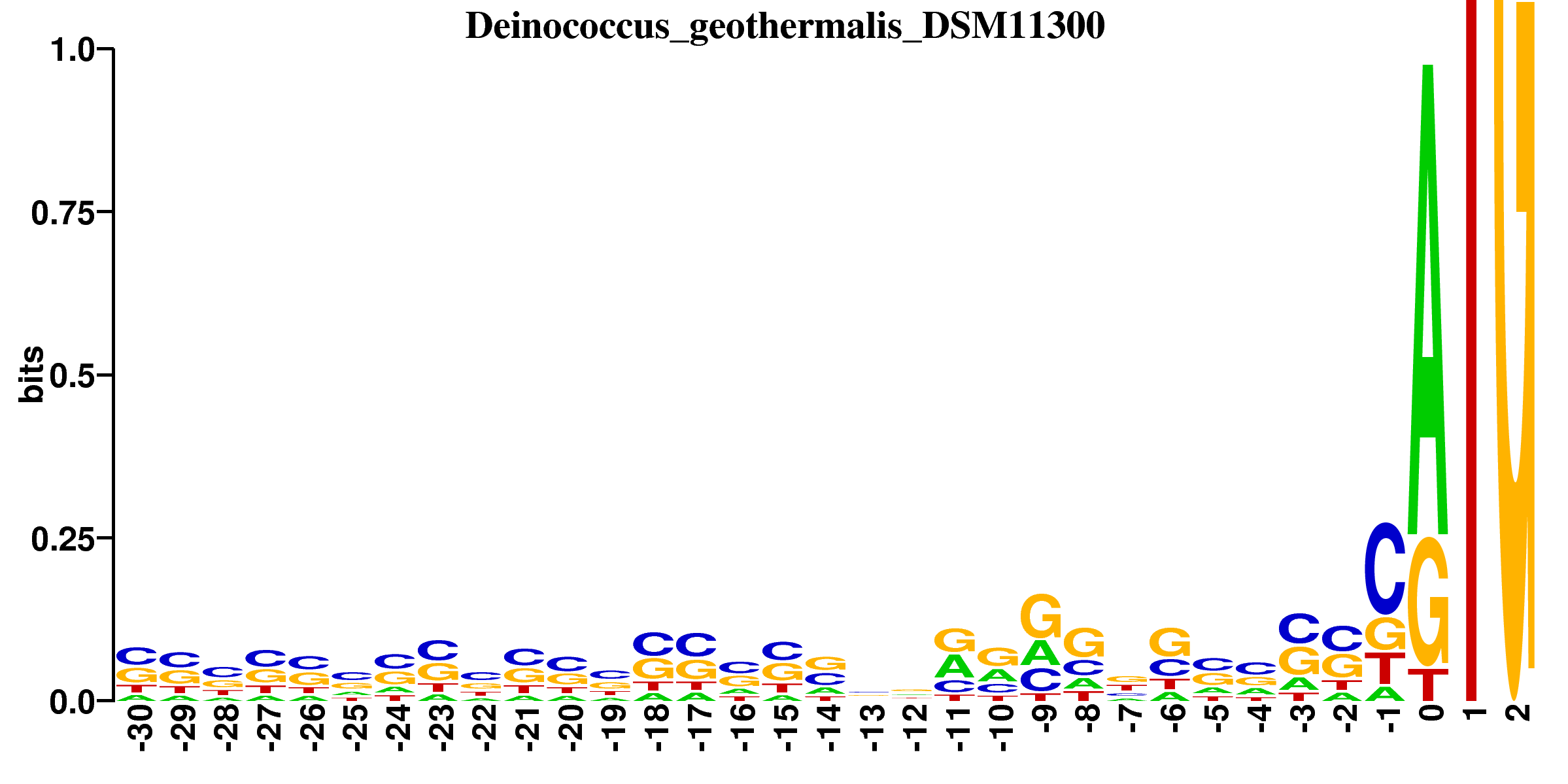
***

| genome % GC | start codon upstream region % GC | difference %GC | genome size [ Mb] |
| --- | --- | --- | --- |
| 66,5 | 64,2 | 2,3 | 3,3 |

***
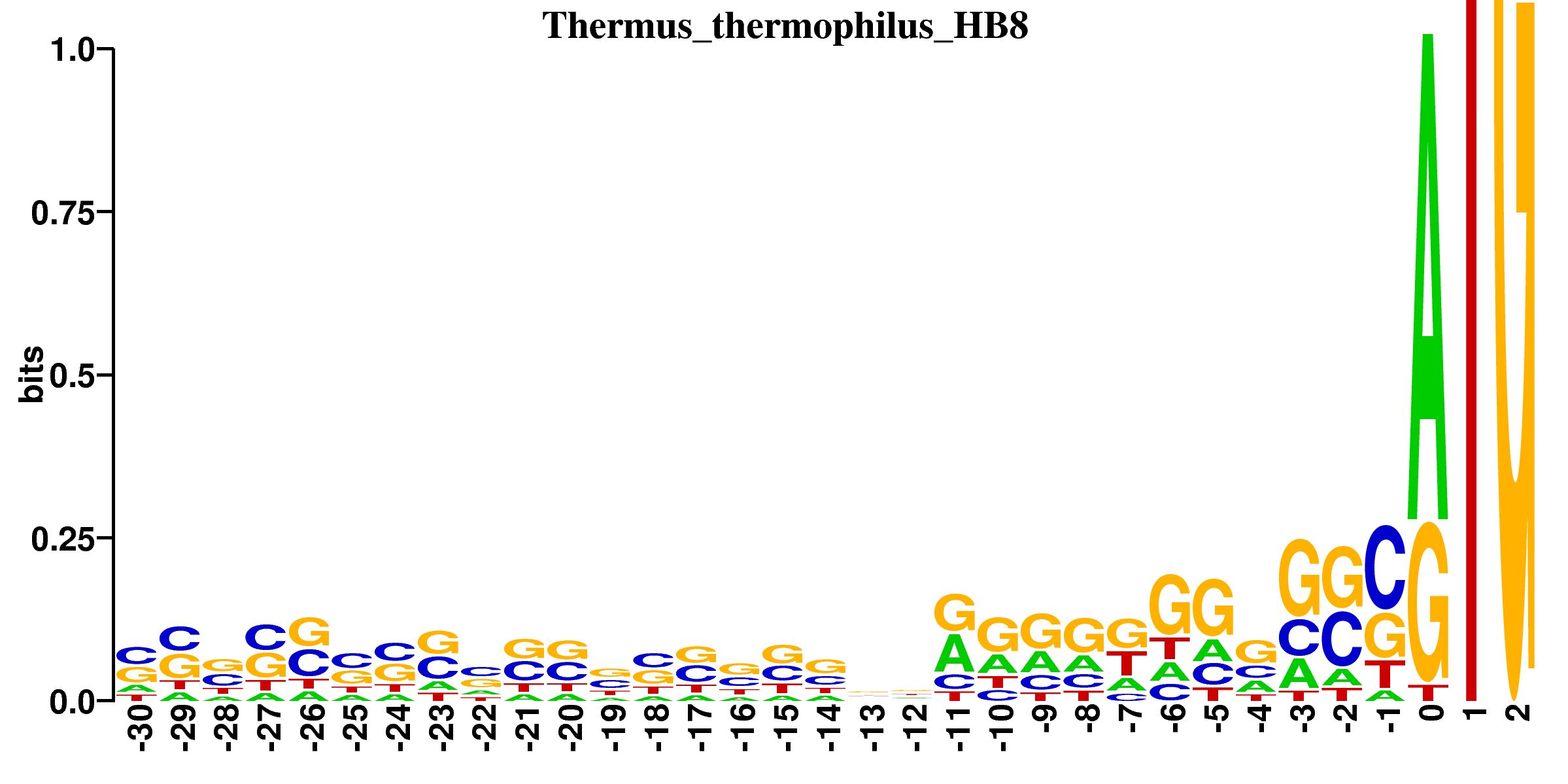
***

| genome % GC | start codon upstream region % GC | difference %GC | genome size [ Mb] |
| --- | --- | --- | --- |
| 69,5 | 64,9 | 4,6 | 2,1 |
