# Supplementary material for: Inability of Prevotella bryantii to Form a Functional Shine-Dalgarno Interaction Reflects Unique Evolution of Ribosome Binding Sites in Bacteroidetes
Source: PLoS One. 2011 Aug 12;6(8):e22914. doi: 10.1371/journal.pone.0022914 (PMC3155529; doi:10.1371/journal.pone.0022914)
Supplement: Figure S14 — Sequence logos of start codon upstream regions of Actinobacteria . (DOC) [file pone.0022914.s014.doc]

***ACTINOBACTERIA***

***
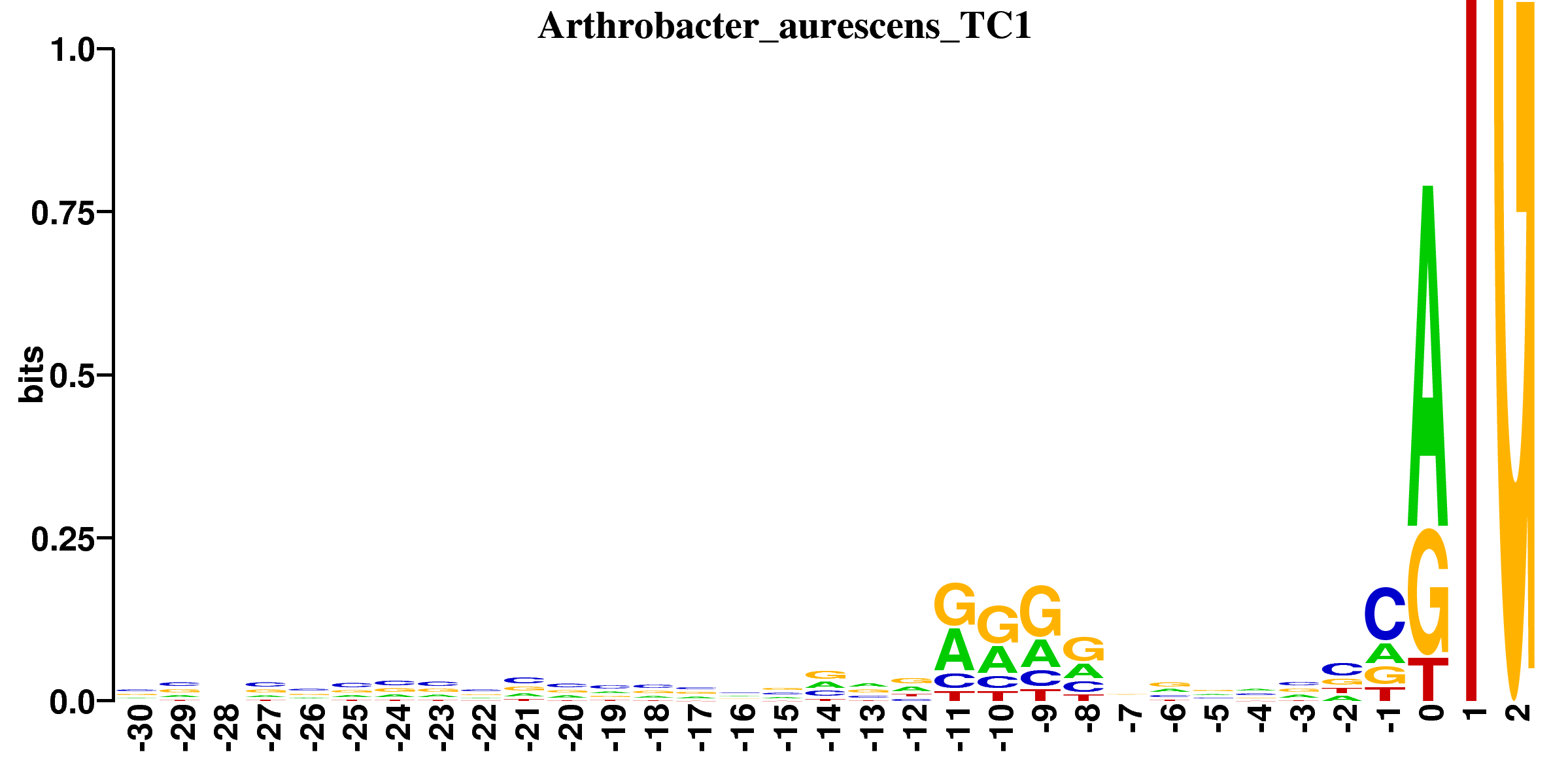
***

| genome % GC | start codon upstream region % GC | difference %GC | genome size [ Mb] |
| --- | --- | --- | --- |
| 62,3 | 57,6 | 4,7 | 4,6 |

***
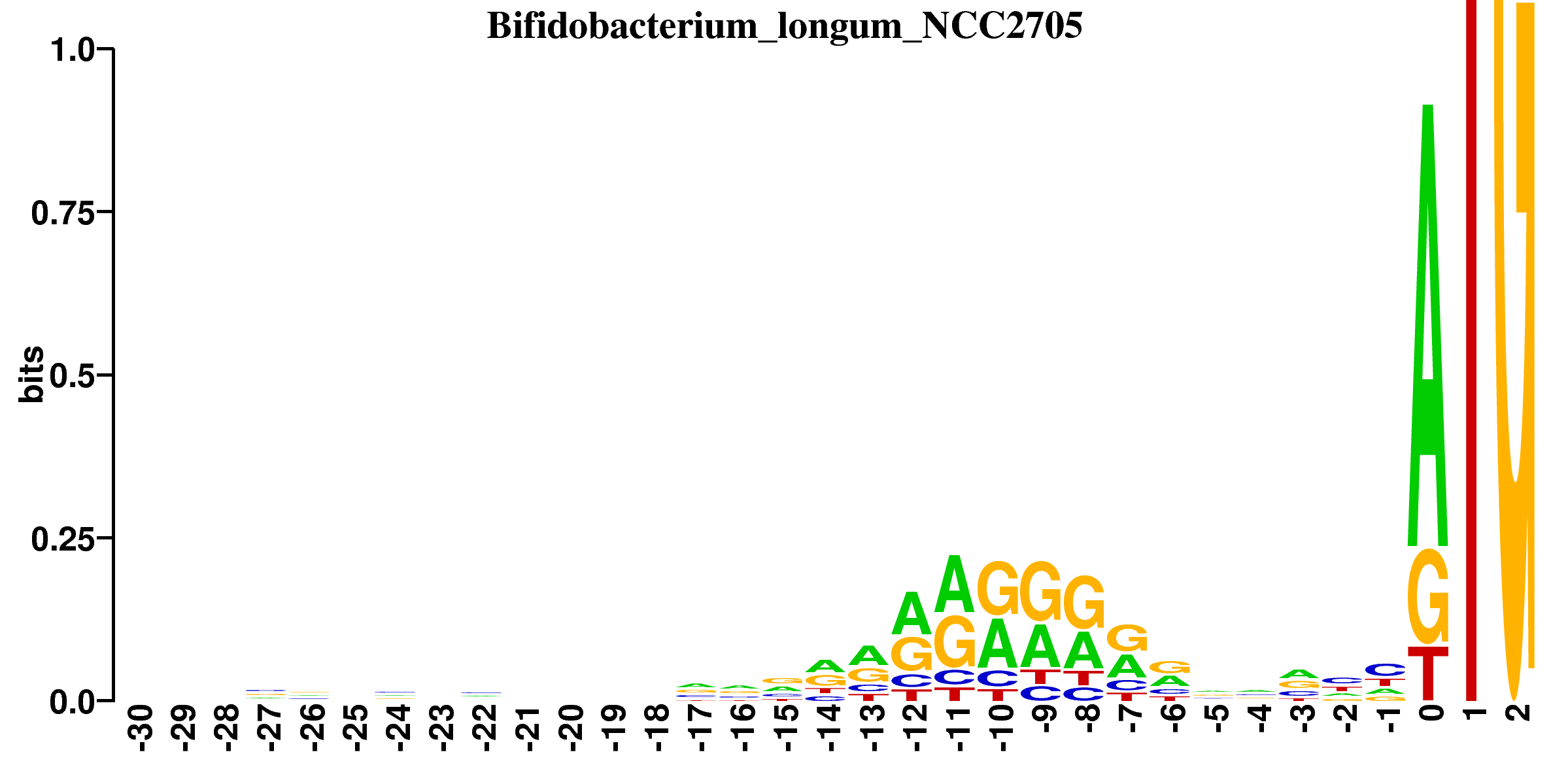
***

| genome % GC | start codon upstream region % GC | difference %GC | genome size [ Mb] |
| --- | --- | --- | --- |
| 60,2 | 52 | 8,2 | 2,4 |


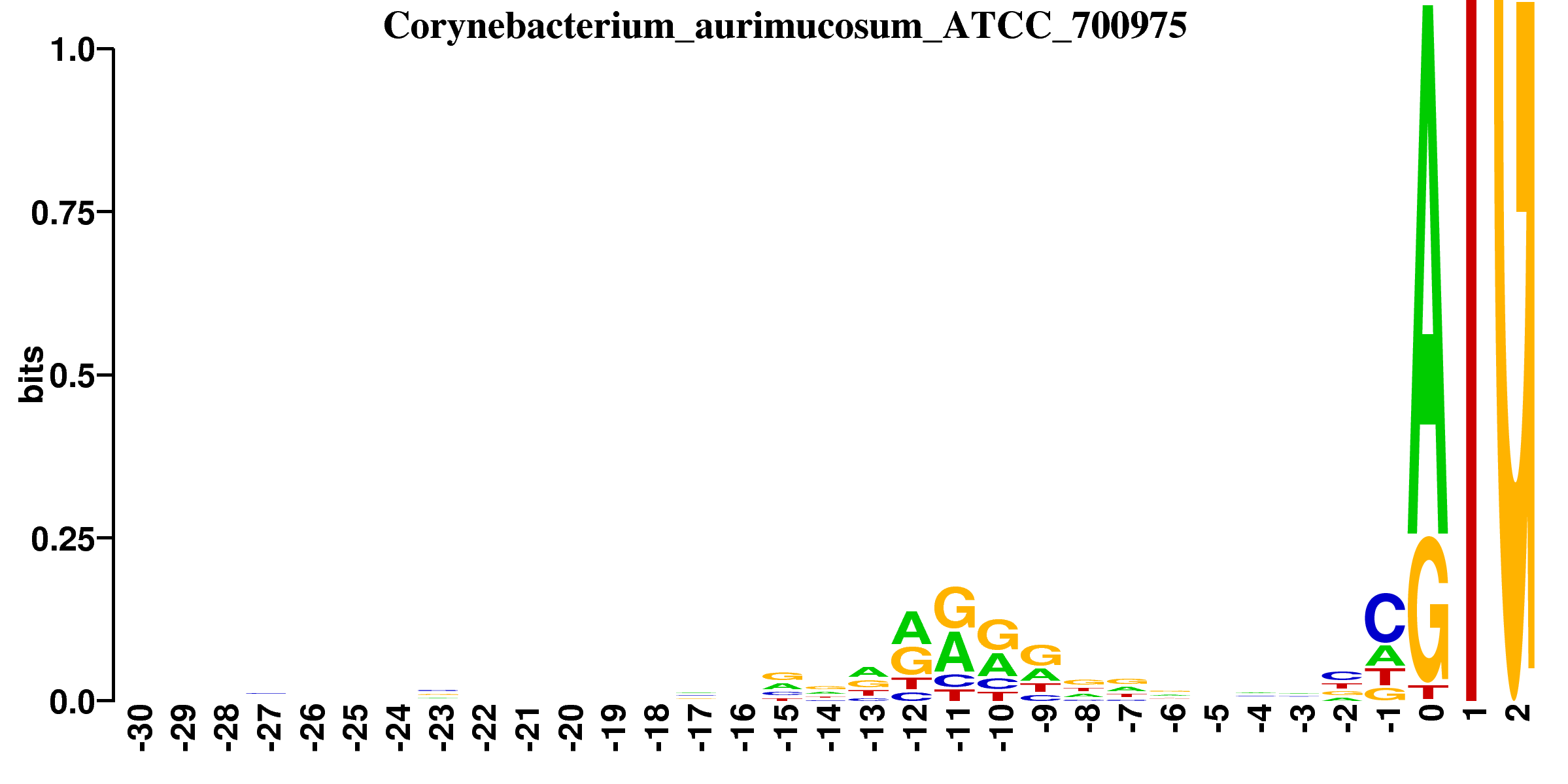


| genome % GC | start codon upstream region % GC | difference %GC | genome size [ Mb] |
| --- | --- | --- | --- |
| 60,6 | 52,8 | 7,8 | 2,8 |


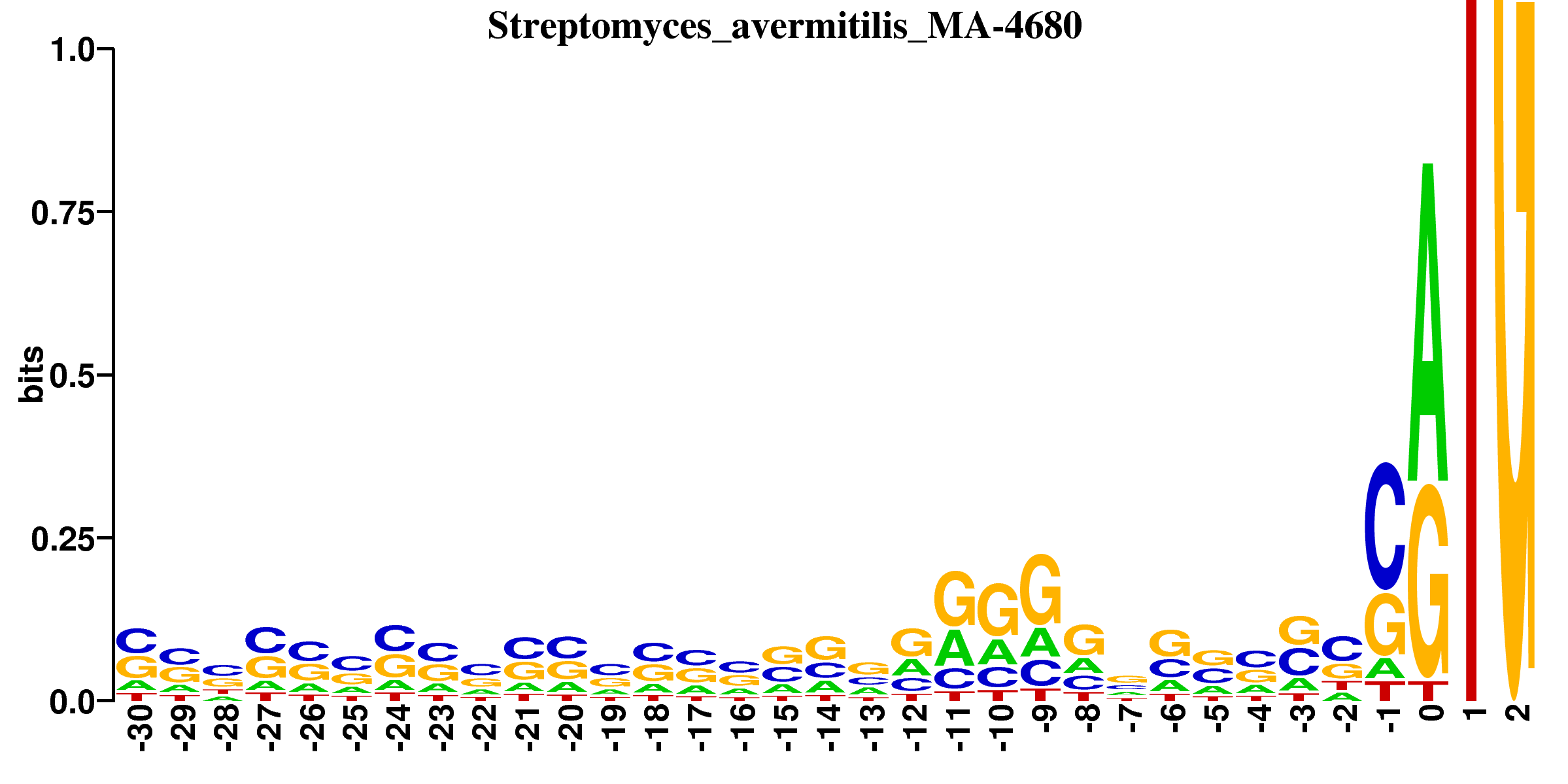


| genome % GC | start codon upstream region % GC | difference %GC | genome size [ Mb] |
| --- | --- | --- | --- |
| 70,7 | 66,4 | 4,3 | 9,1 |


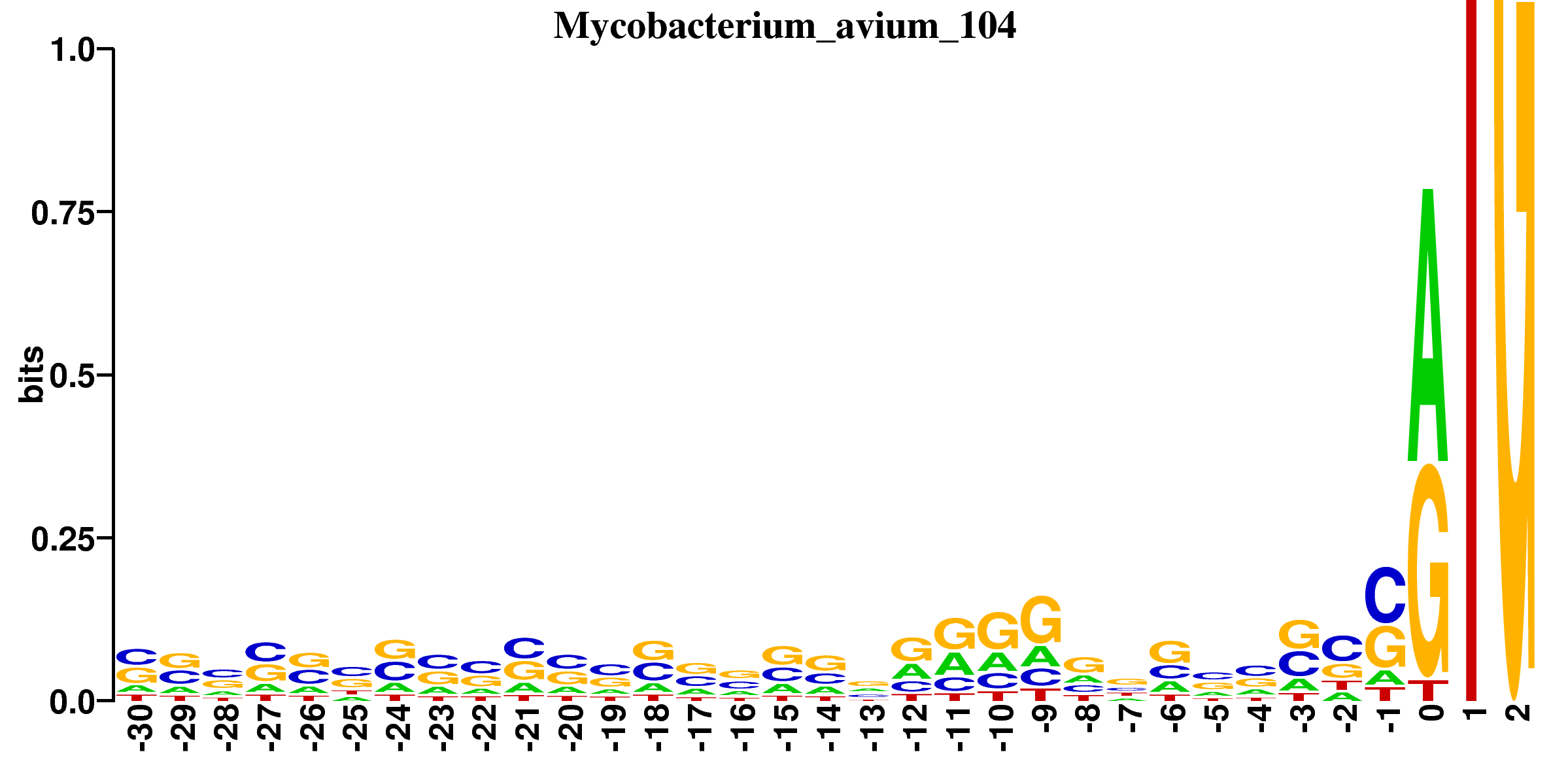


| genome % GC | start codon upstream region % GC | difference %GC | genome size [ Mb] |
| --- | --- | --- | --- |
| 69 | 64,6 | 4,4 | 5,5 |
