# Supplementary material for: Inability of Prevotella bryantii to Form a Functional Shine-Dalgarno Interaction Reflects Unique Evolution of Ribosome Binding Sites in Bacteroidetes
Source: PLoS One. 2011 Aug 12;6(8):e22914. doi: 10.1371/journal.pone.0022914 (PMC3155529; doi:10.1371/journal.pone.0022914)
Supplement: Figure S15 — Sequence logos of start codon upstream regions of Tenericutes . (DOC) [file pone.0022914.s015.doc]

***TENERICUTES***

**
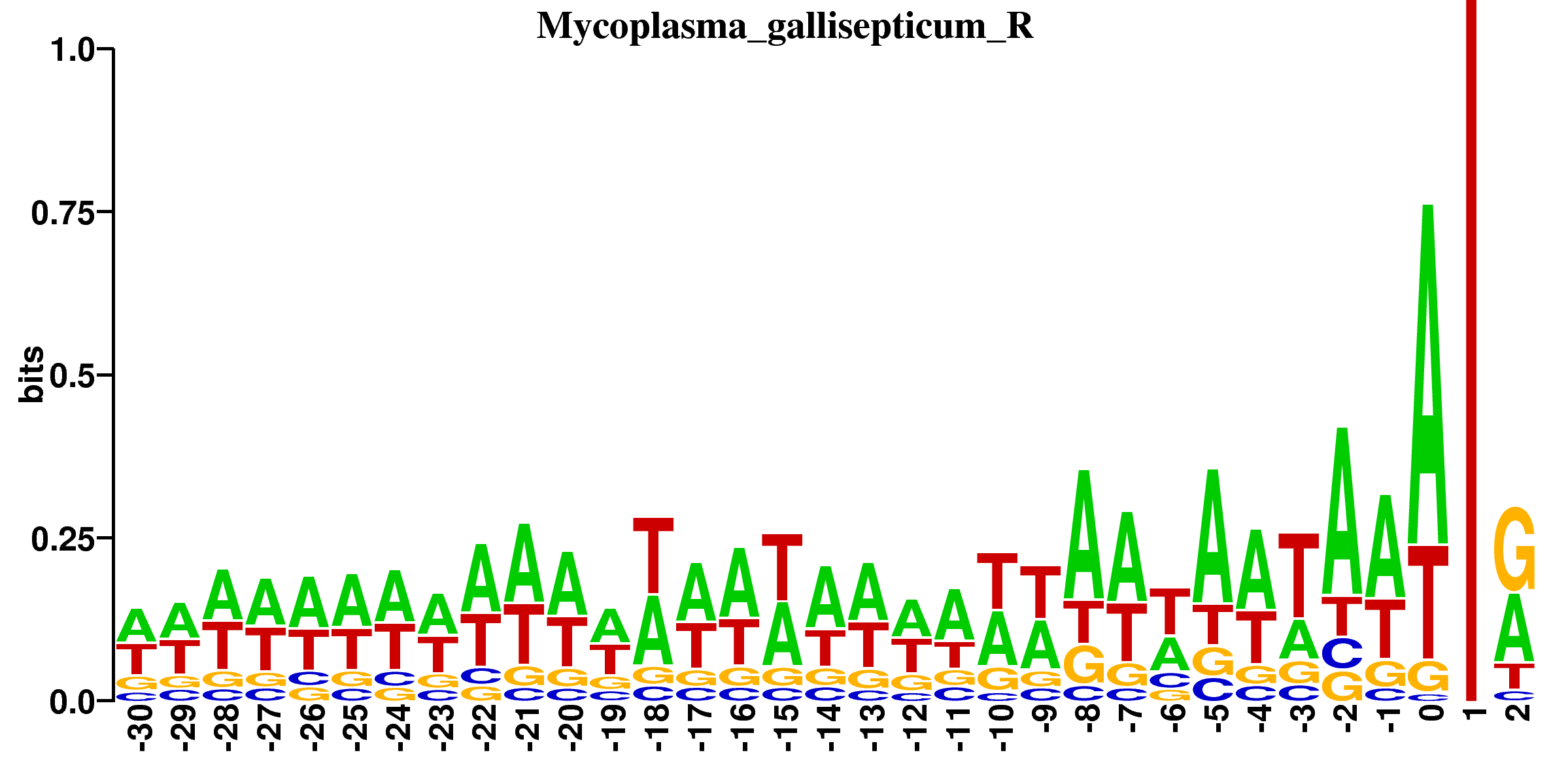
**

| genome % GC | start codon upstream region % GC | difference %GC | genome size [ Mb] |
| --- | --- | --- | --- |
| 31,5 | 24,1 | 7,4 | 1 |

**
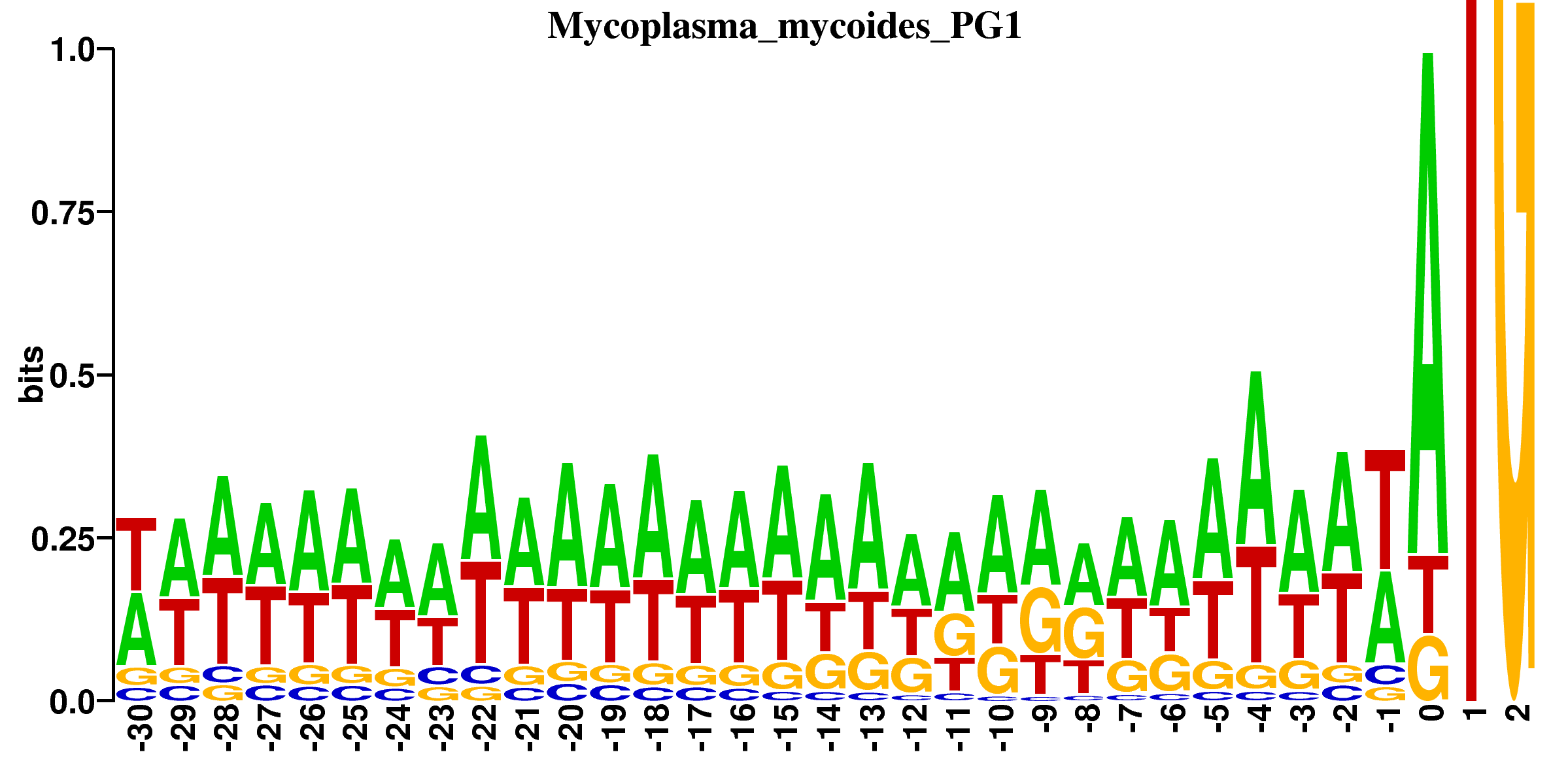
**

| genome % GC | start codon upstream region % GC | difference %GC | genome size [ Mb] |
| --- | --- | --- | --- |
| 24 | 20,7 | 3,3 | 1,2 |
