# Supplementary material for: Inability of Prevotella bryantii to Form a Functional Shine-Dalgarno Interaction Reflects Unique Evolution of Ribosome Binding Sites in Bacteroidetes
Source: PLoS One. 2011 Aug 12;6(8):e22914. doi: 10.1371/journal.pone.0022914 (PMC3155529; doi:10.1371/journal.pone.0022914)

Figure S16

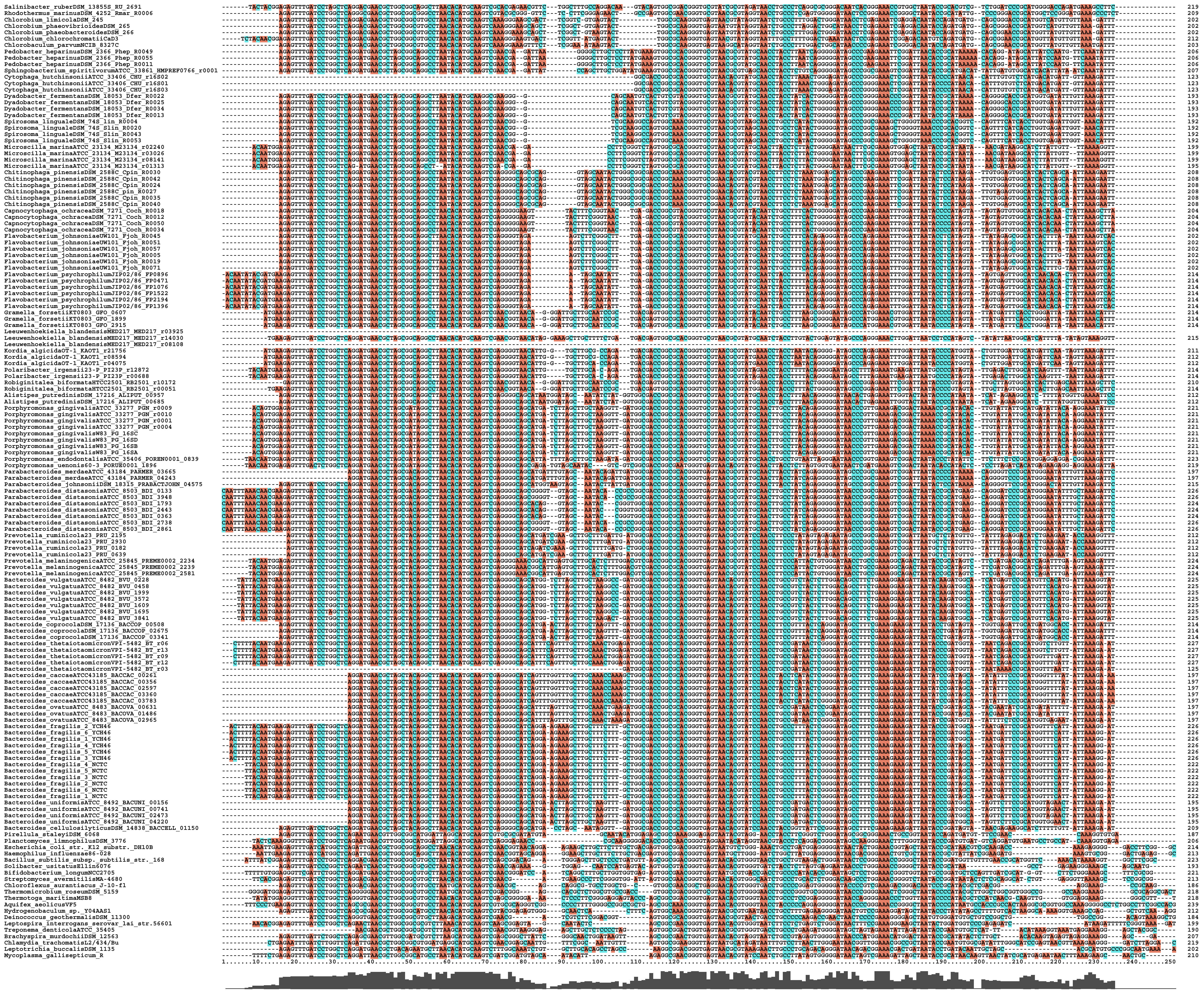

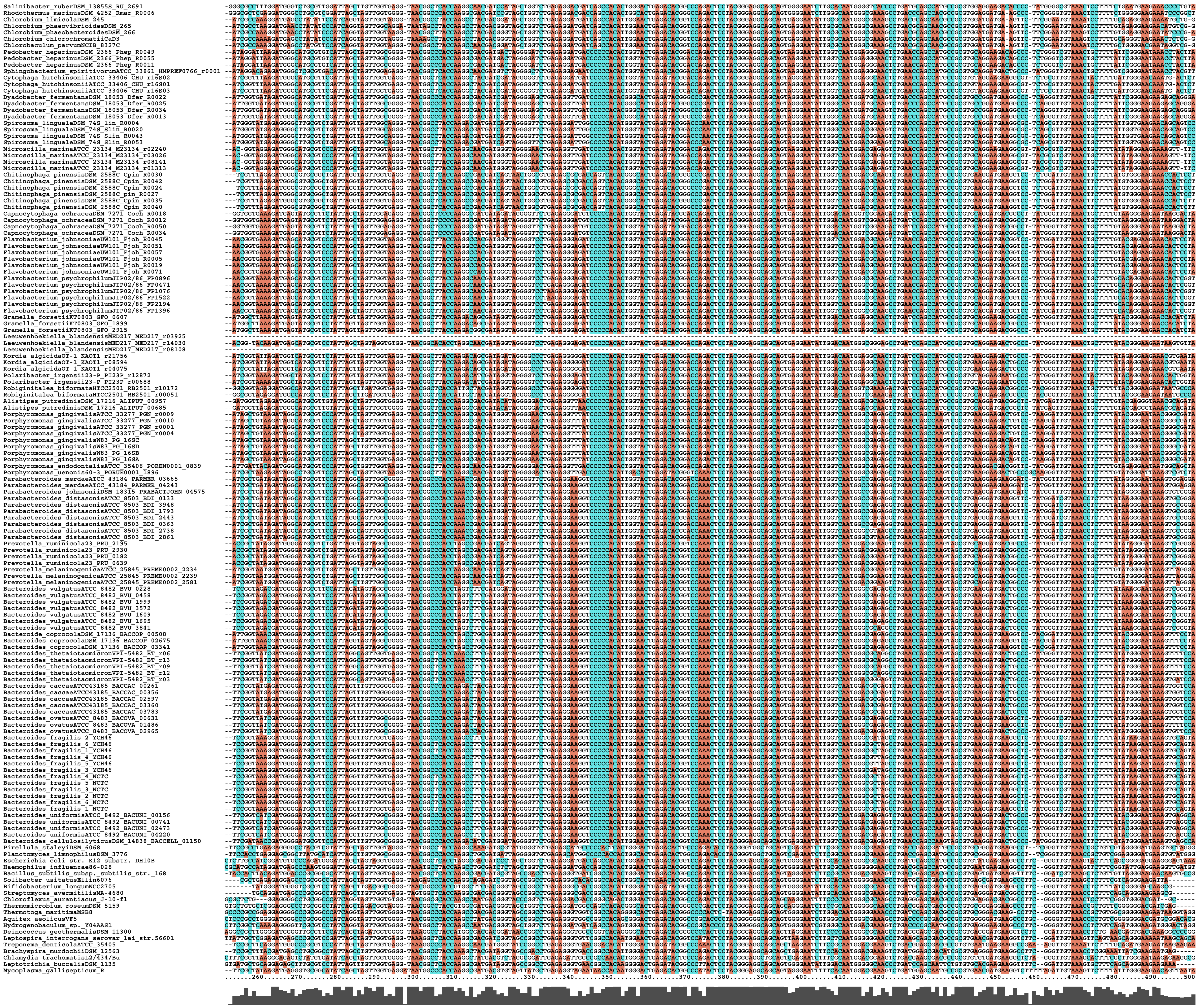

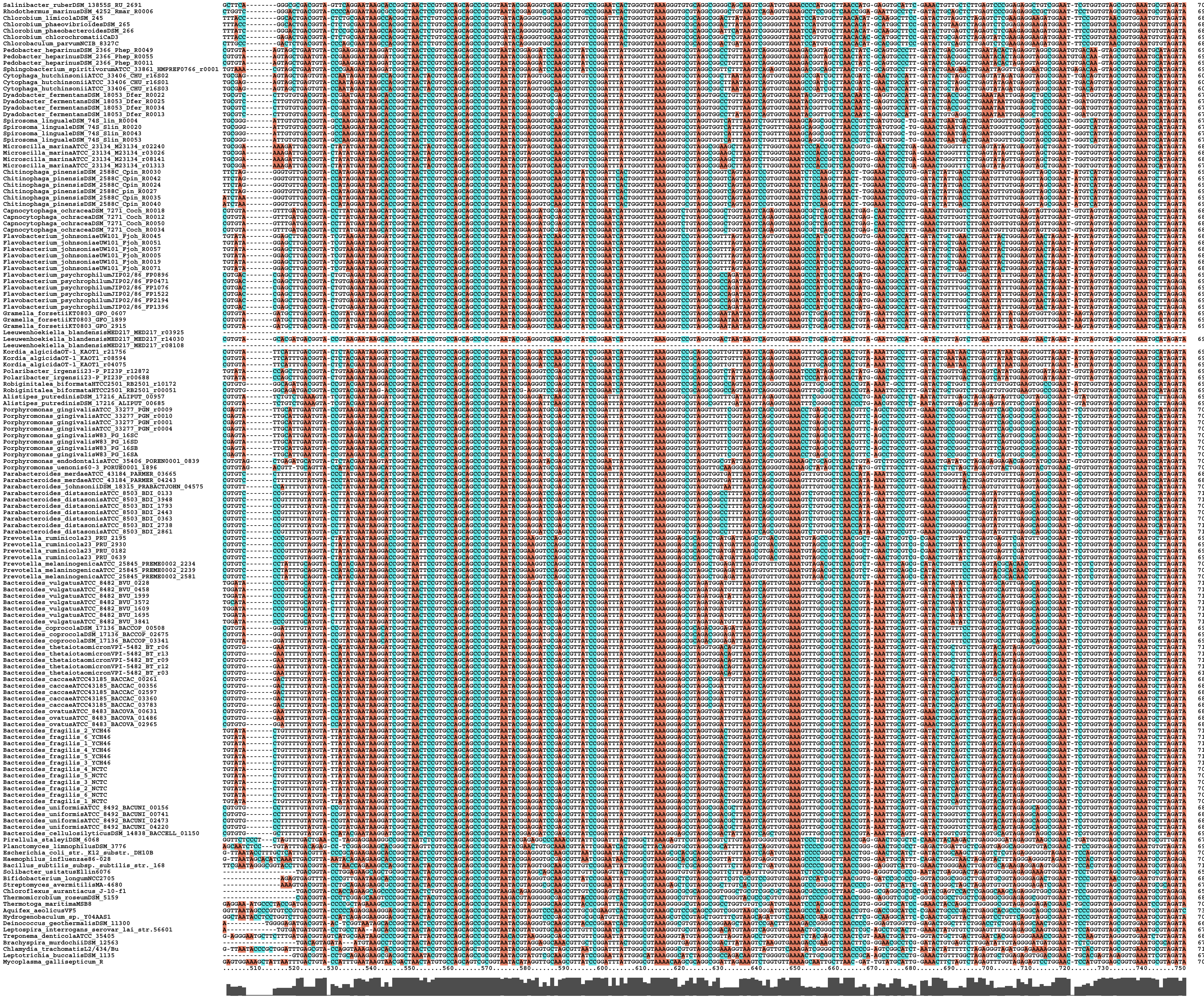

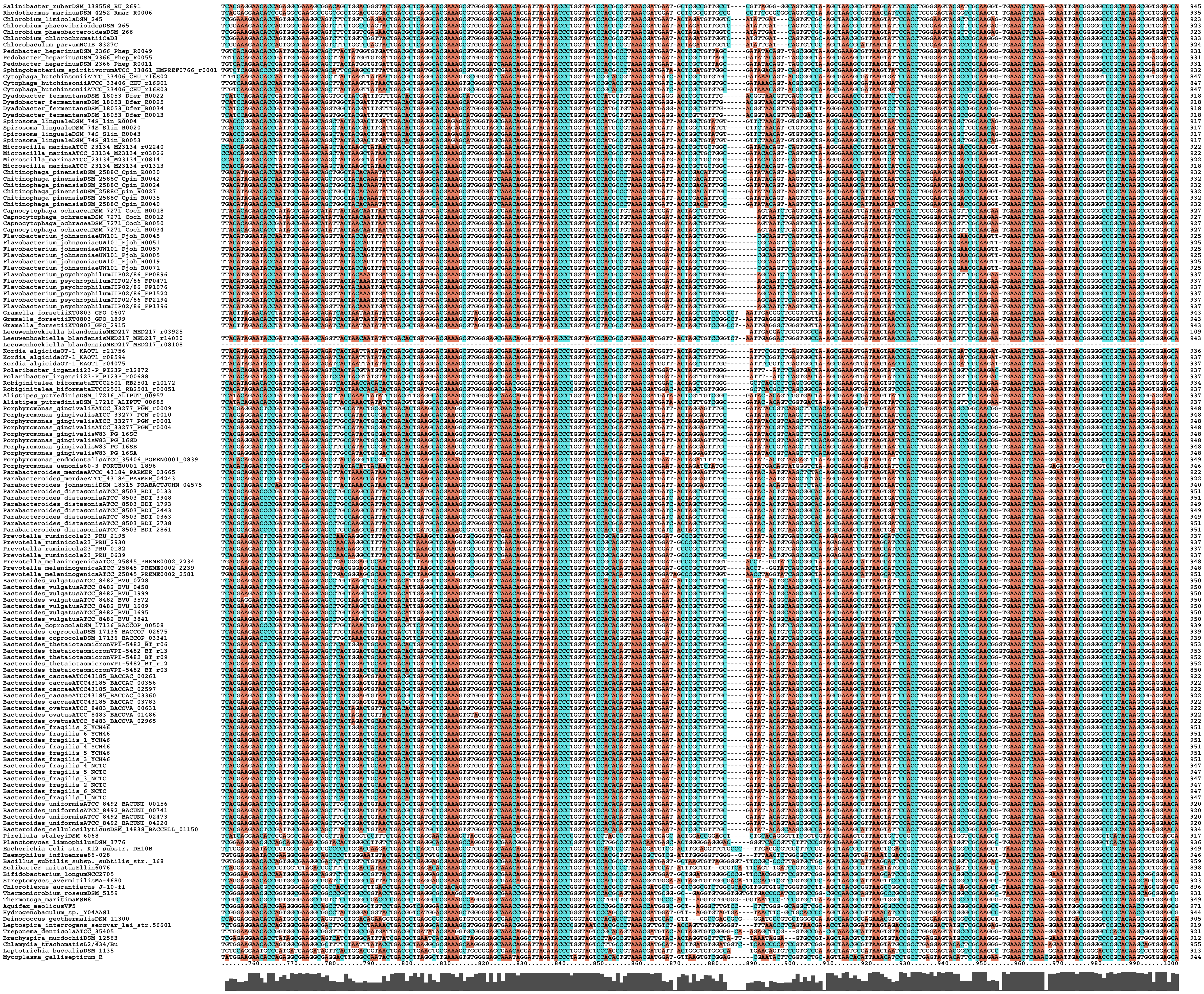

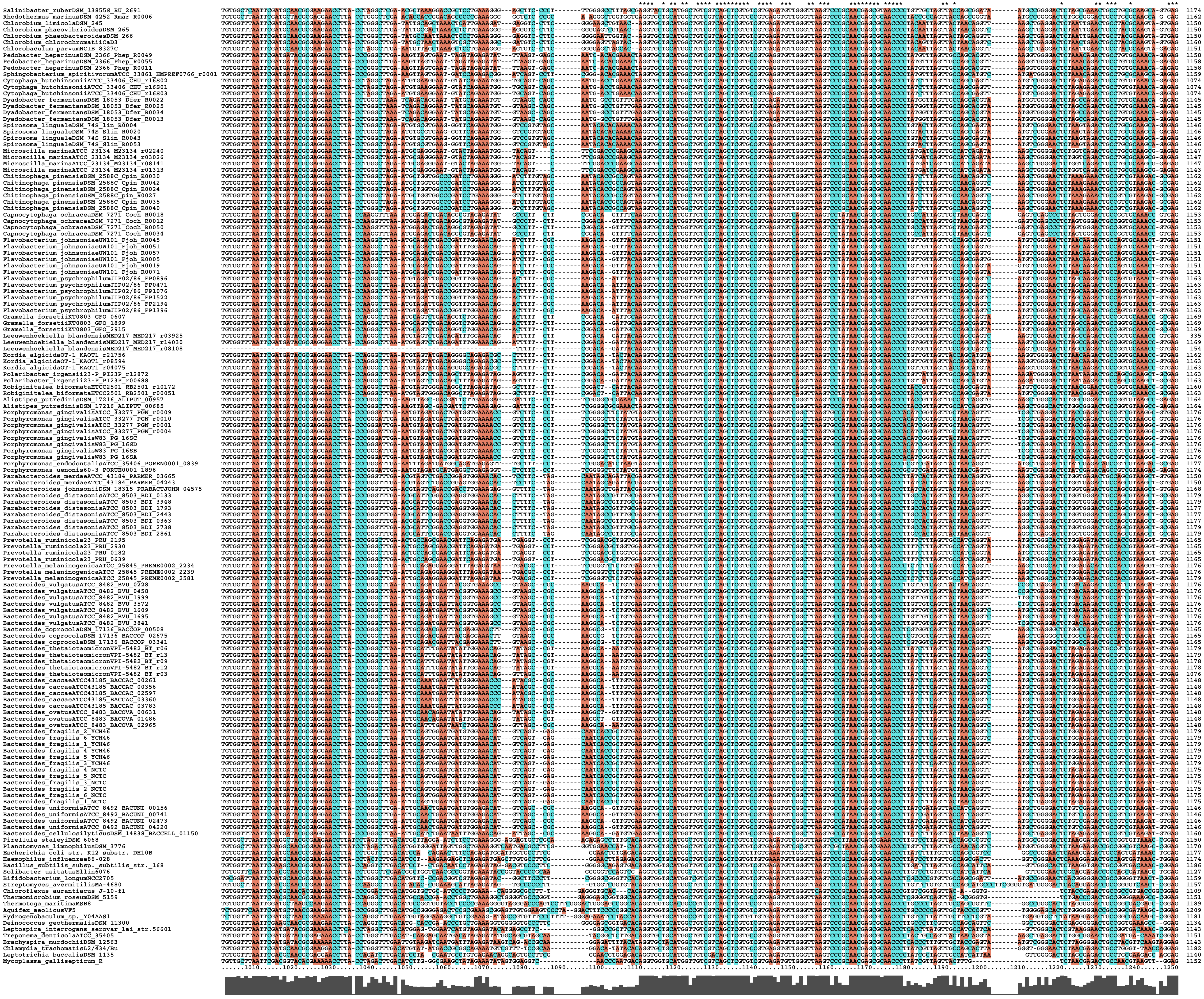

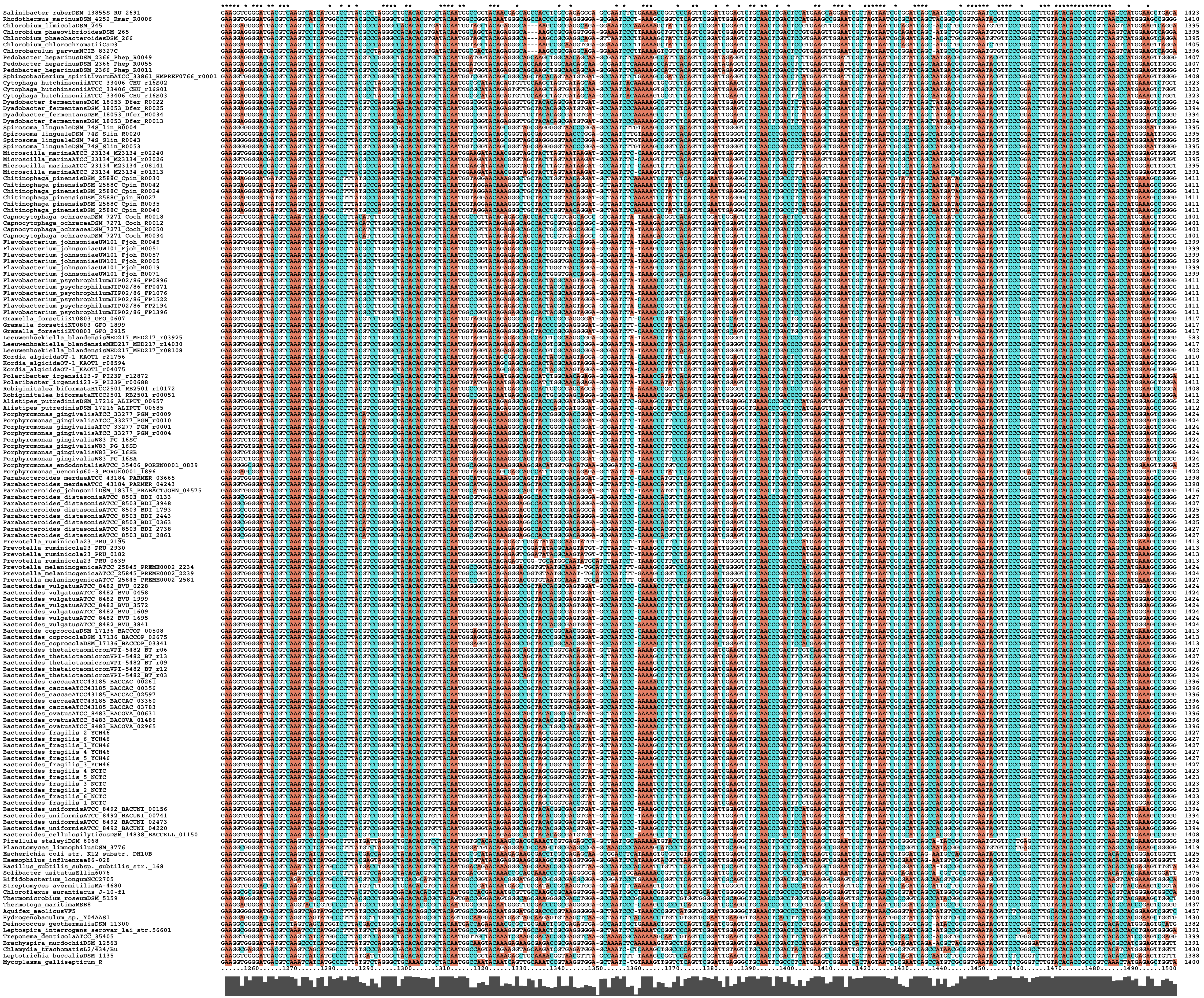

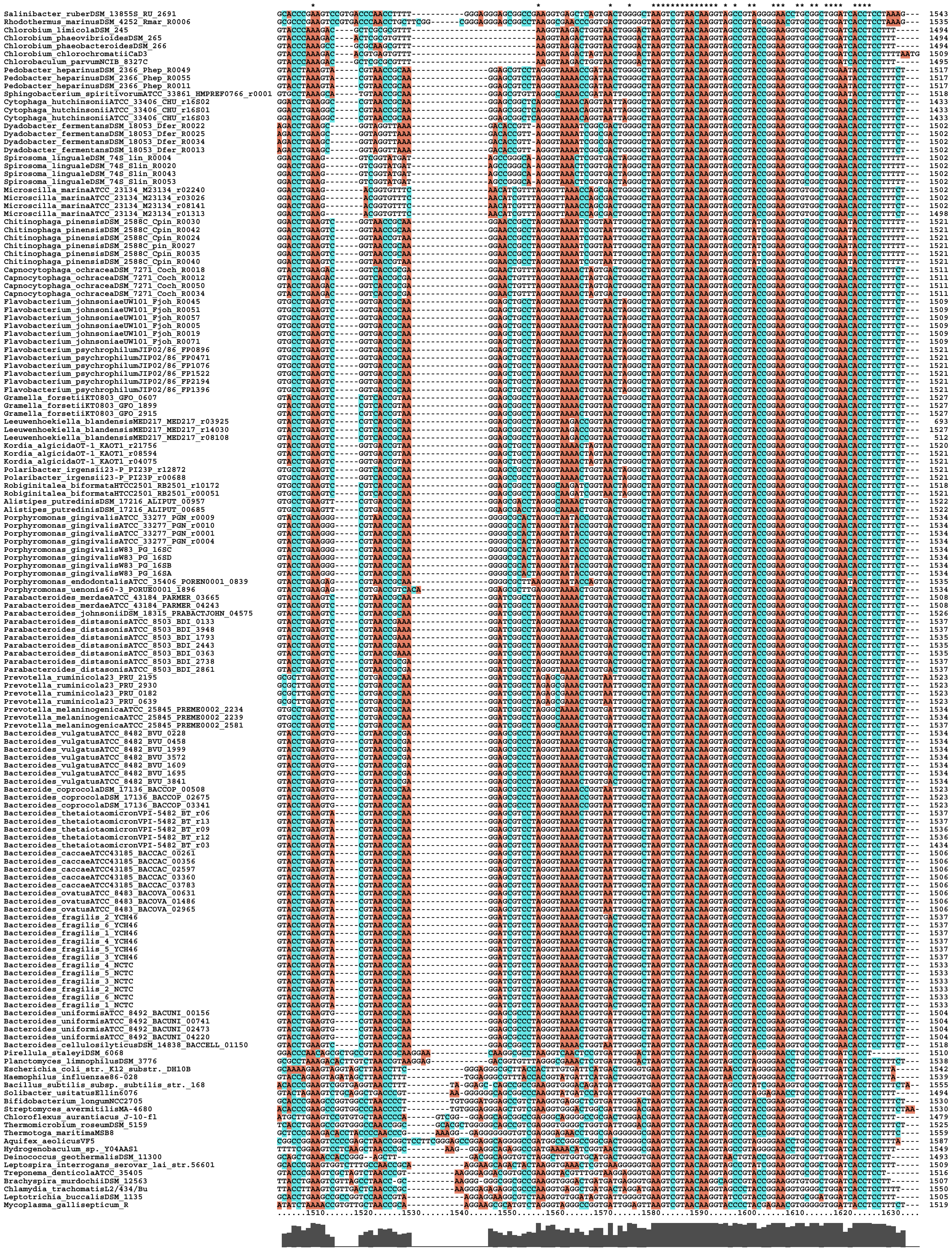

Supplement: Figure S16 — Multiple sequence alignment of 16S rRNA genes from Bacteroidetes and representatives of other bacterial phyla. (PDF) [file pone.0022914.s016.pdf]
