# Supplementary material for: Inability of Prevotella bryantii to Form a Functional Shine-Dalgarno Interaction Reflects Unique Evolution of Ribosome Binding Sites in Bacteroidetes
Source: PLoS One. 2011 Aug 12;6(8):e22914. doi: 10.1371/journal.pone.0022914 (PMC3155529; doi:10.1371/journal.pone.0022914)

>upstream sequence 0

TAAACAAAGAAAGTTAAAAATACAACGTTATAAATTAATTTAGGAGGAATATT**ATGCACAC**AATTAAATCTTTATTTTTAGCC

>upstream sequence 11,1

TAAACAAAGAAAGTTAAAAATACAACGTTATCCTCCTATTTAGGAGGAATATT**ATGCACAC**AATTAAATCTTTATTTTTAGCC


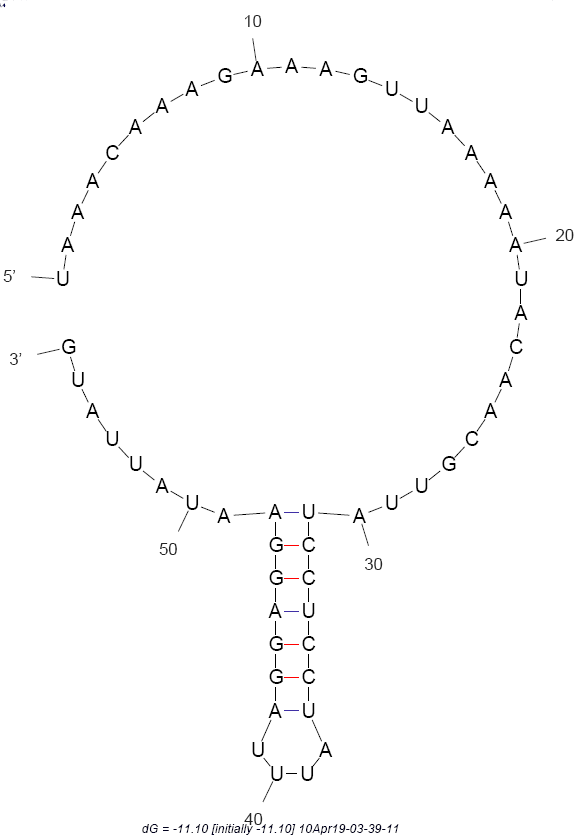


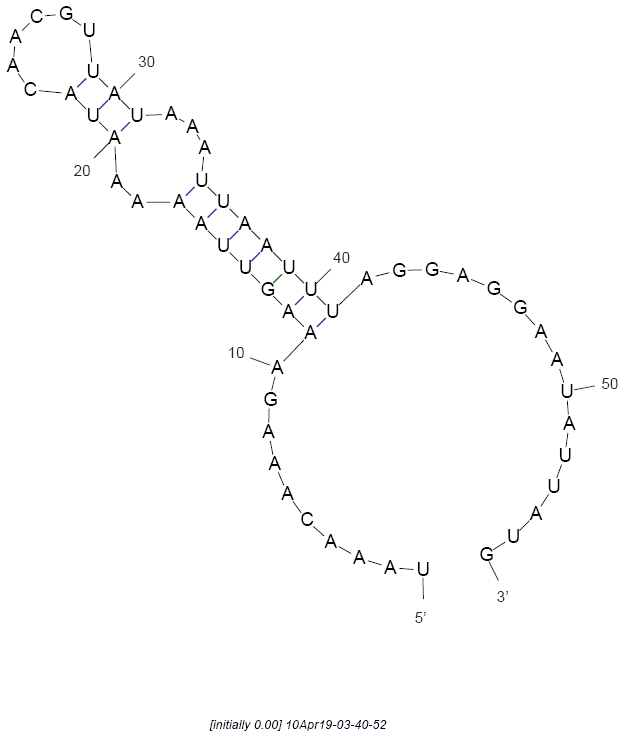


>upstream sequence 5,2

TAAACAAAGAAAGTTAAAAATACAACGTTAACCTCAAATTTAGGAGGAATATTATG**CACAC**AATTAAATCTTTATTTTTAGCC

>upstream sequence 2,1

TAAACAAAGAAAGTTAAAAATACAACGTTAAACTCAAATTTAGGAGGAATATTATG**CACAC**AATTAAATCTTTATTTTTAGCC


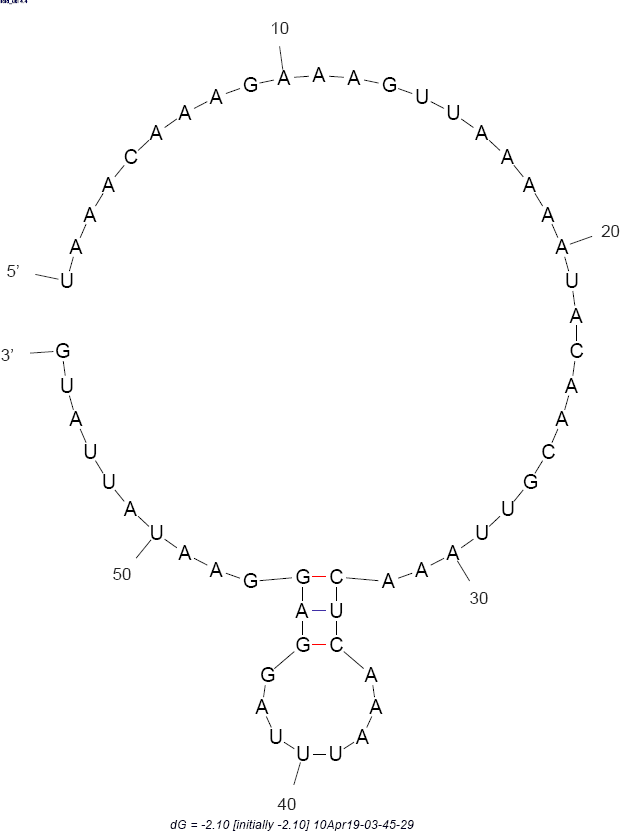

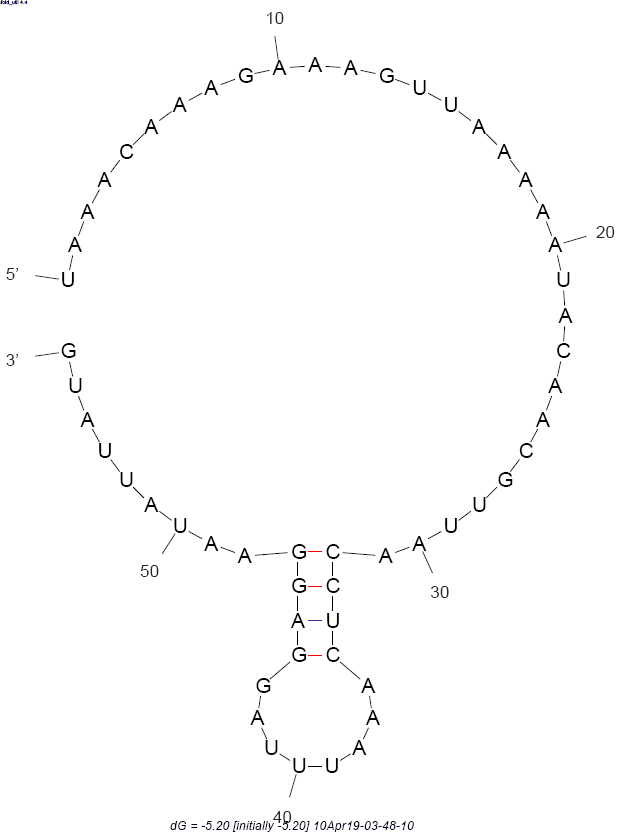


mFold results for mRNA deriving from pRH3 plasmid constructs containing the above upstream sequences. 39 bp of pRH3 preceding the *Pst*I site were included in calculation.


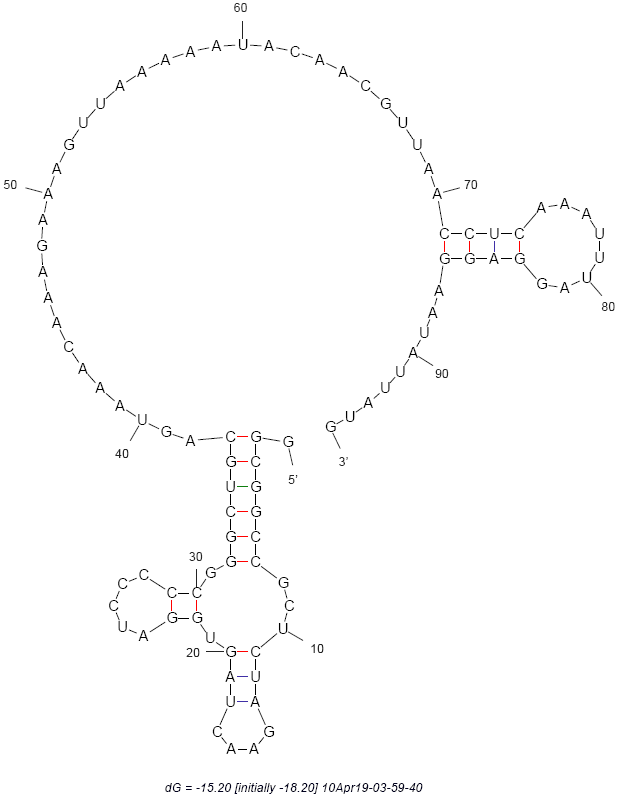

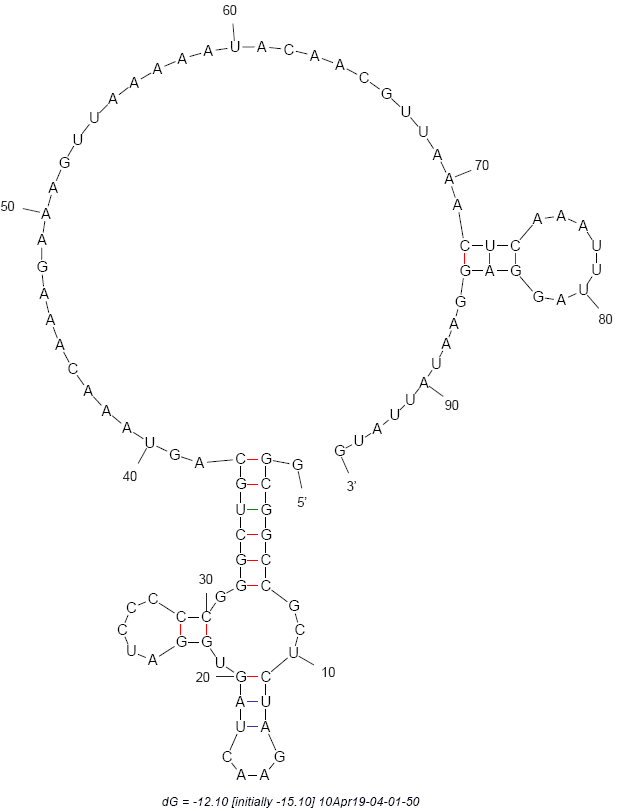

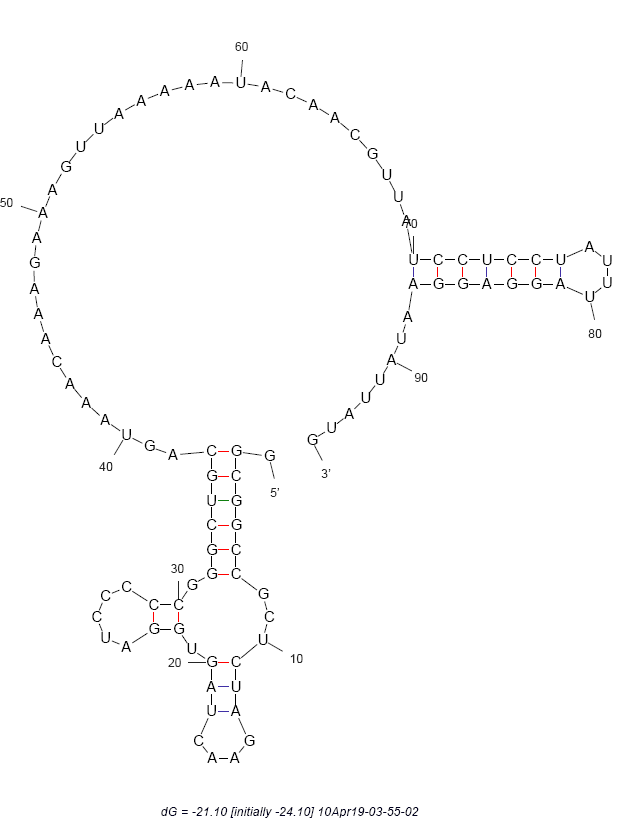


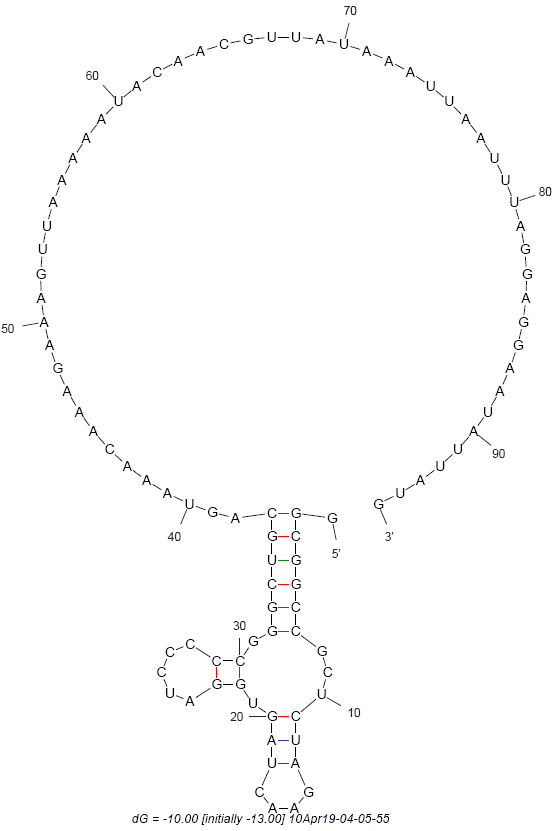


11,1 5,2 2,1 0

>upstream sequence -20

GCCGCTGCAGTAAACAAAGAAAGTTAAAAATACAACGTTATAAGCGCGAAAACGCGCATTAATTTAGGAGGAATATTATGCACACAATTAAATCTTTATTTTTAGCC


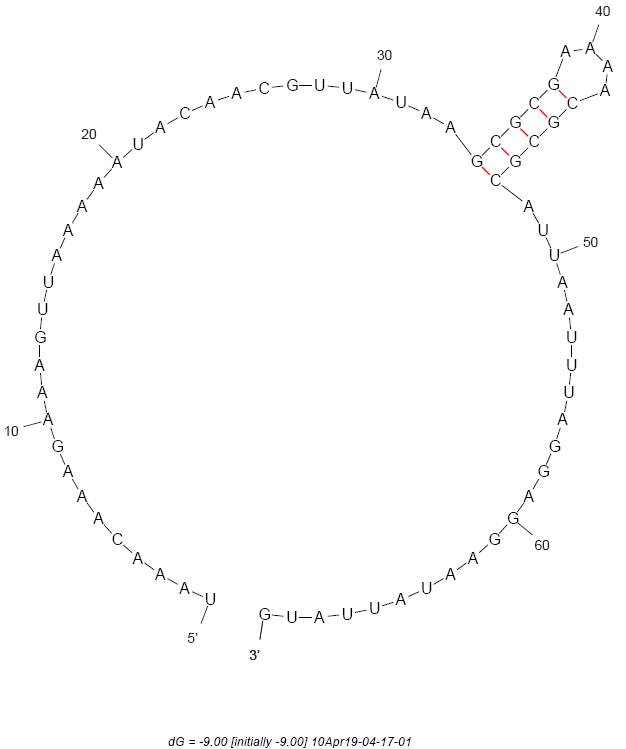


>upstream sequence 11,1inv

GCCGCTGCAGTAAACAAAGAAAGTTAAAAATACAACGTTATAGGAGGATTTCCTCCTAATATTATGCACACAATTAAATCTTTATTTTTAGCC

>upstream sequence 5,4inv

GCCGCTGCAGTAAACAAAGAAAGTTAAAAATACAACGTTAAGAGGAAATTTAGCCTCAATATTATGCACACAATTAAATCTTTATTTTTAGCC


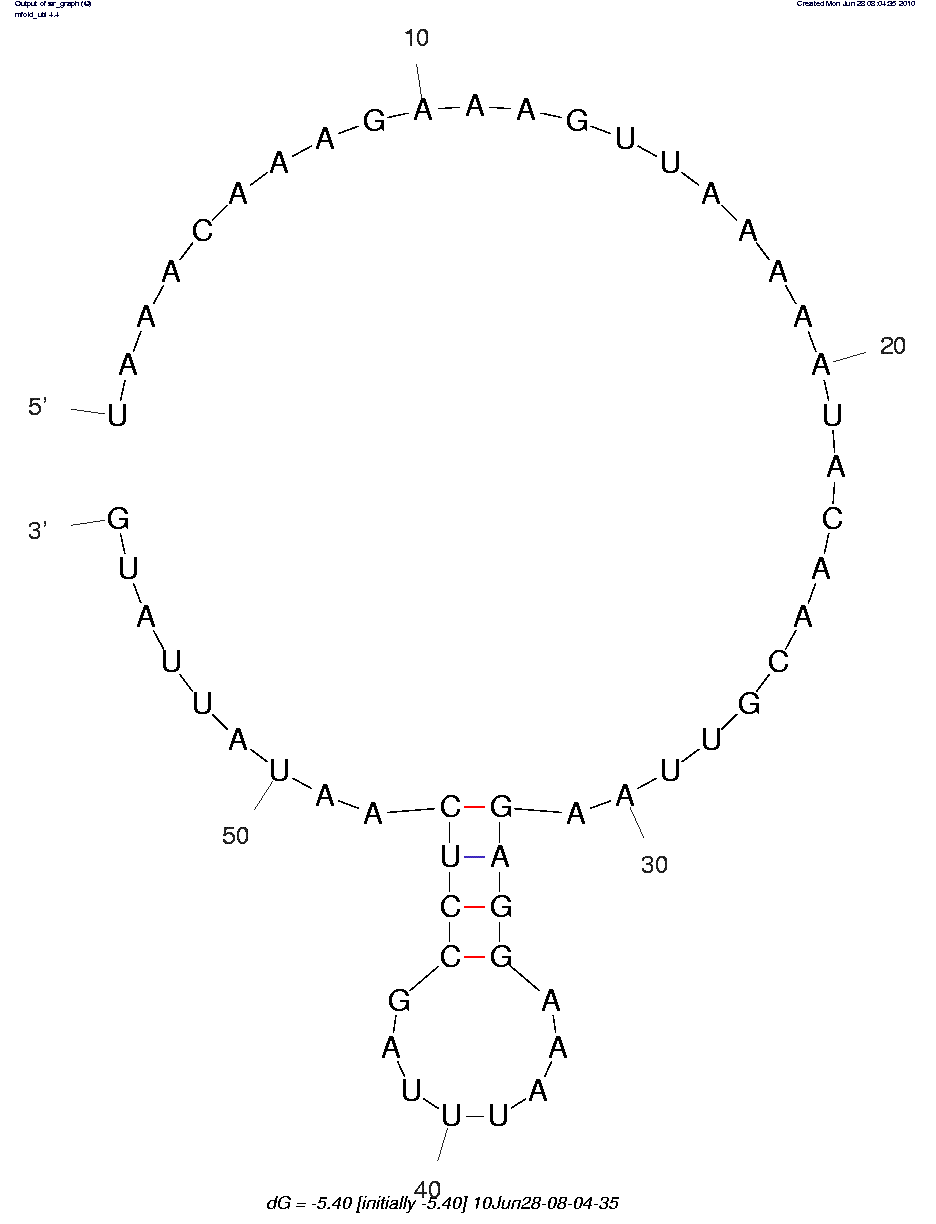

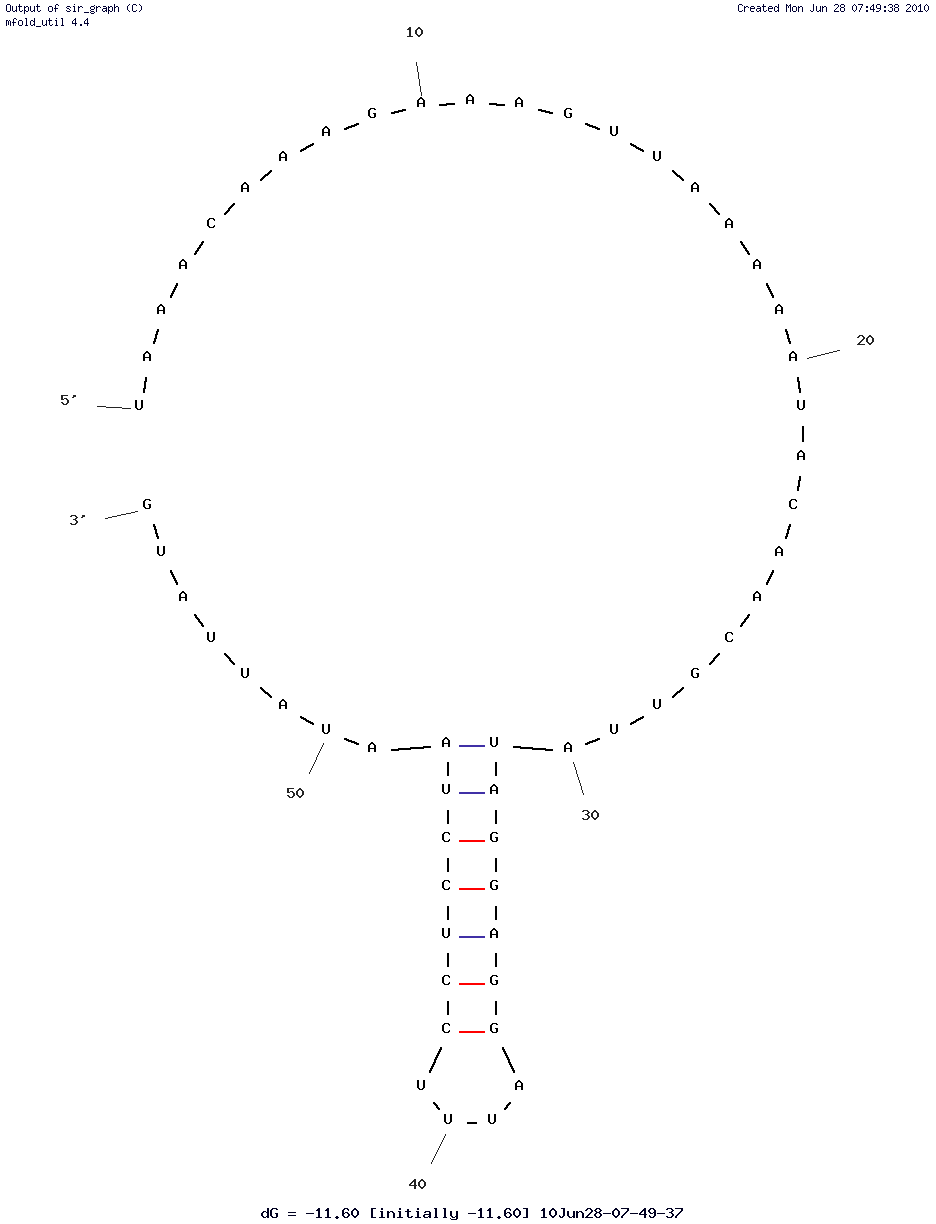

Supplement: Figure S17 — mRNA secondary structure prediction of 11,1, 5,2, 2,1, 0, 11,1inv, 5,4inv and -20 start codon upstream regions. (DOC) [file pone.0022914.s017.doc]
