# Supplementary material for: Inability of Prevotella bryantii to Form a Functional Shine-Dalgarno Interaction Reflects Unique Evolution of Ribosome Binding Sites in Bacteroidetes
Source: PLoS One. 2011 Aug 12;6(8):e22914. doi: 10.1371/journal.pone.0022914 (PMC3155529; doi:10.1371/journal.pone.0022914)
Supplement: Figure S18 — A typical western blot used for quantification. (DOC) [file pone.0022914.s018.doc]

1 2 3 4 5


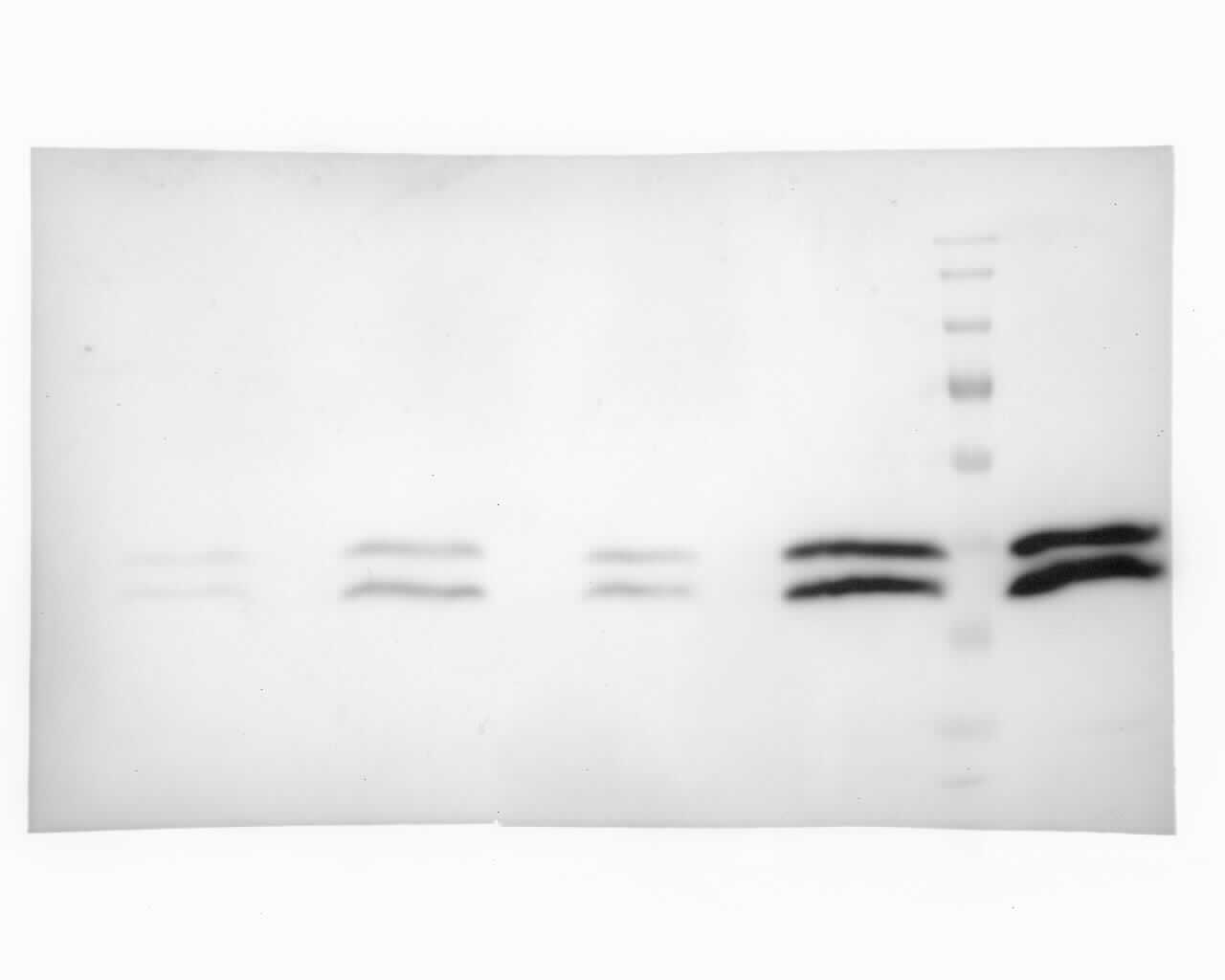


43 kDa

34 kDa

A typical western blot of NucB concentrated from culture supernatant used for relative quantification.

Lanes: 1, SD6; 2, SD8; 3, SD10; 4, *nucB* original upstream sequence; 5. PINA-1201 original upstream sequence.
