# Supplementary material for: Inability of Prevotella bryantii to Form a Functional Shine-Dalgarno Interaction Reflects Unique Evolution of Ribosome Binding Sites in Bacteroidetes
Source: PLoS One. 2011 Aug 12;6(8):e22914. doi: 10.1371/journal.pone.0022914 (PMC3155529; doi:10.1371/journal.pone.0022914)
Supplement: Figure S19 — mRNA secondary structure prediction of PINA_1201, nucB , SD6, SD8 and SD10 start codon upstream regions. (DOC) [file pone.0022914.s019.doc]

Secondary structure of PINA_1201 start codon upstream sequence as predicted by mfold.

Secondary structure of *nucB* start codon upstream sequence as predicted by mfold.

Secondary structure of SD10 start codon upstream sequence as predicted by mfold.

Secondary structure of SD8 start codon upstream sequence as predicted by mfold.

Secondary structure of SD6 start codon upstream sequence as predicted by mfold.
