# Supplementary material for: Inability of Prevotella bryantii to Form a Functional Shine-Dalgarno Interaction Reflects Unique Evolution of Ribosome Binding Sites in Bacteroidetes
Source: PLoS One. 2011 Aug 12;6(8):e22914. doi: 10.1371/journal.pone.0022914 (PMC3155529; doi:10.1371/journal.pone.0022914)
Supplement: Table S1 — GC content in the start codon upstream regions in major bacterial phyla. (DOC) [file pone.0022914.s020.doc]

|  | **genome GC%** | **% GC of 30 bp preceding the start codon** | **∆ GC** | **genome size [Mb]** |
| --- | --- | --- | --- | --- |
|  |  |  |  |  |
| ***ACTINOBACTERIA*** |  |  |  |  |
|  |  |  |  |  |
| *Acidothermus cellulolyticus* 11B | 66.9 | 66.4 | 0.5 | 2.4 |
| *Arthrobacter aurescens* TC1 | 62.3 | 57.6 | 4.7 | 4.6 |
| *Bifidobacterium longum* NCC2705 | 60.2 | 52 | 8.2 | 2.4 |
| *Corynebacterium efficiens* YS-314 | 63.1 | 56.1 | 7 | 3.1 |
| *Corynebacterium glutamicum* ATCC 13032 | 53.8 | 46.6 | 7.2 | 3.1 |
| *Corynebacterium diphtheriae* NCTC 13129 | 53.5 | 47.7 | 5.8 | 2.5 |
| *Corynebacterium aurimucosum* ATCC 700975 | 60.6 | 52.8 | 7.8 | 2.8 |
| *Mycobacterium avium* 104 | 69 | 64.6 | 4.4 | 5.5 |
| *Propionibacterium acnes* KPA171202 | 60 | 57.9 | 2.1 | 2.6 |
| *Rubrobacter xylanophilus* DSM 9941 | 70.5 | 66.5 | 4 | 3.2 |
| *Streptomyces avermitilis* MA-4680 | 70.7 | 66.4 | 4.3 | 9.1 |
|  |  |  | mean | ∆ GC STD |
|  |  |  | 5.090909 | 2.409752911 |
| ***ACIDOBACTERIA*** |  |  |  |  |
|  |  |  |  |  |
| *Acidobacterium capsulatum* ATCC 51196 | 60.5 | 55.5 | 5 | 4.1 |
| Candidatus *Koribacter versatilis* ellin345 | 58.4 | 53.9 | 4.5 | 5.8 |
| *Solibacter usitatus* Ellin6076 | 61.9 | 54.1 | 7.8 | 10 |
|  |  |  | mean | ∆ GC STD |
|  |  |  | 5.766667 | 1.77857621 |
|  |  |  |  |  |
| ***CHLAMYDIAE*** |  |  |  |  |
|  |  |  |  |  |
| *Chlamydia muridarum* Nigg | 40.3 | 35.8 | 4.5 | 1.1 |
| *Chlamydia trachomatis* 434/Bu | 41.3 | 37 | 4.3 | 1 |
| *Chlamydophila pneumoniae* AR39 | 40.6 | 33.4 | 7.2 | 1.23 |
| Candidatus *Protochlamydia amoebophila* UWE25 | 34.7 | 29.4 | 5.3 | 2.41 |
|  |  |  | mean | ∆ GC STD |
|  |  |  | 5.325 | 1.322560648 |
|  |  |  |  |  |
| ***CHLOROBI*** |  |  |  |  |
|  |  |  |  |  |
| *Chlorobaculum parvum* NCIB 8327 | 55.8 | 44.6 | 11.2 | 2.3 |
| *chlorobium chlorochromatii* CaD3 | 44.3 | 34.7 | 9.6 | 2.6 |
| *Chlorobium phaeobacteroides* DSM 266 | 48.9 | 39.6 | 9.3 | 2.7 |
| *Chlorobium phaeovibrioides* DSM 265 | 53 | 44.7 | 8.3 | 2 |
| *Chlorobium tepidum* TLS | 56.5 | 46.1 | 10.4 | 2.2 |
| *Chlorobium limicola* DSM 245 | 51.3 | 40.9 | 10.4 | 2.8 |
| *Chloroherpeton thalassium* ATCC 35110 | 45 | 32.6 | 12.4 | 3.3 |
| *Pelodictyon luteolum* DSM 273 | 57.3 | 49 | 8.3 | 2.4 |
| *Pelodictyon phaeoclathratiforme* BU-1 | 48.1 | 38.4 | 9.7 | 3 |
| *Prosthecochloris aestuarii* DSM 271 | 50.1 | 40.5 | 9.6 | 2.6 |
|  |  |  | mean | ∆ GC STD |
|  |  |  | 9.92 | 1.253262409 |
|  |  |  |  |  |
| ***CHLOROFLEXI*** |  |  |  |  |
|  |  |  |  |  |
| *Chloroflexus aurantiacus* J-10-fl | 56.7 | 50.6 | 6.1 | 5.3 |
| *Herpetosiphon aurantiacus* ATCC 23779 | 50.9 | 46.1 | 4.8 | 6.7 |
| *Roseiflexus castenholzii* DSM 13941 | 60.7 | 54.5 | 6.2 | 5.7 |
| *Thermomicrobium roseum* DSM 5159 | 63.7 | 63.9 | -0.2 | 2.9 |
|  |  |  | mean | ∆ GC STD |
|  |  |  | 4.225 | 3.018139604 |
|  |  |  |  |  |
| ***CYANOBACTERIA*** |  |  |  |  |
|  |  |  |  |  |
| *Acaryochloris marina* MBIC11017 | 47 | 42.7 | 4.3 | 8.4 |
| *Anabaena variabilis* ATCC 29413 | 41.4 | 34.7 | 6.7 | 7.1 |
| *Gloeobacter violaceus* PCC 7421 | 62 | 55.8 | 6.2 | 4.7 |
| *Nostoc punctiforme* PCC 73102 | 41.4 | 34.2 | 7.2 | 9 |
| *Prochlorococcus marinus* NATL1A | 35 | 28.2 | 6.8 | 1.9 |
| *Prochlorococcus marinus* AS9601 | 31.3 | 21.6 | 9.7 | 1.7 |
| *Synechococcus elongatus* PCC 6301 | 55.5 | 53.3 | 2.2 | 2.7 |
| *Synechococcus* sp. JA-3-3ab | 60.2 | 55 | 5.2 | 2.9 |
|  |  |  | mean | ∆ GC STD |
|  |  |  | 6.0375 | 2.212262901 |
|  |  |  |  |  |
| ***FIBROBACTERES*** |  |  |  |  |
|  |  |  |  |  |
| *Fibrobacter succinogenes* S85 | 48 | 36.5 | 11.5 | 3.8 |
|  |  |  |  |  |
| ***FIRMICUTES*** |  |  |  |  |
|  |  |  |  |  |
| *Bacillus licheniformis* ATCC 14580 | 46.2 | 40.1 | 6.1 | 4.2 |
| *Bacillus subtilis* subsp. *subtilis* str. 168 | 43.5 | 39.4 | 4.1 | 4.2 |
| *Clostridium acetobutylicum* ATCC 824 | 30.9 | 28.7 | 2.2 |  |
| *Clostridium thermocellum* ATCC 27405 | 39 | 35.12 | 3.88 | 3.8 |
| *Listeria monocytogenes* str. 4b F2365 | 38 | 35 | 3 | 2.9 |
| *Moorella thermoacetica* ATCC 39073 | 55.8 | 47.8 | 8 | 2.6 |
| *Mycoplasma gallisepticum* strain R | 31.5 | 24.1 | 7.4 | 1 |
| *Mycoplasma mycoides* subsp. *mycoides* SC str. PG1 | 24 | 20.7 | 3.3 | 1.2 |
| *Streptococcus pneumoniae* D39 | 39.7 | 33.4 | 6.3 | 2 |
|  |  |  | mean | ∆ GC STD |
|  |  |  | 4.92 | 2.073547685 |
|  |  |  |  |  |
| ***AQUIFICAE*** |  |  |  |  |
|  |  |  |  |  |
| *Aquifex aeolicus* VF5 | 43.5 | 34.8 | 8.7 | 1.6 |
| *Hydrogenobaculum* sp. Y04AAS1 | 34.8 | 28.5 | 6.3 | 1.6 |
| *Persephonella marina* EX-H1 | 37.2 | 32.1 | 5.1 | 2 |
| *Sulfurihydrogenibium azorense* Az-Fu1 | 32.8 | 29.4 | 3.4 | 1.6 |
|  |  |  | mean | ∆ GC STD |
|  |  |  | 5.875 | 2.227666941 |
|  |  |  |  |  |
| ***PROTEOBACTERIA*** |  |  |  |  |
|  |  |  |  |  |
| ***ALFA*** |  |  |  |  |
| *Bartonella quintana* str. Toulouse | 38.8 | 33.6 | 5.2 | 1.6 |
| *Bartonella tribocorum* CIP 105476 | 38.8 | 34.9 | 3.9 | 2.6 |
| *Caulobacter crescentus* CB15 | 67.2 | 63.7 | 3.5 | 4 |
| *Erythrobacter litoralis* HTCC2594 | 63.1 | 60 | 3.1 | 3 |
| *Gluconobacter oxydans* 621H | 60.8 | 56.9 | 3.9 | 2.9 |
| *Magnetospirillum magneticum* AMB-1 | 65.1 | 61.2 | 3.9 | 5 |
| *Nitrobacter hamburgensis* X14 | 61.6 | 57.8 | 3.8 | 5 |
| *Phenylobacterium zucineum* HLK1 | 71.1 | 68.1 | 3 | 4.4 |
| *Rhizobium leguminosarum* bv. viciae | 55 | 54.8 | 0.2 | 7.8 |
| *Rhodobacter sphaeroides* ATCC 17025 | 68.2 | 65.9 | 2.3 | 4.5 |
| *Silicibacter pomeroyi* DSS-3 | 64.2 | 59.8 | 4.4 | 4.1 |
| *Sphingomonas wittichii* RW1 | 67.9 | 63.7 | 4.2 | 5.9 |
| *Zymomonas mobilis* subsp. *mobilis* ZM4 | 46.3 | 40.6 | 5.7 | 2 |
|  |  |  | mean | ∆ GC STD |
|  |  |  | 3.623077 | 1.358402153 |
|  |  |  |  |  |
| ***RICKETTSIALES*** |  |  |  |  |
|  |  |  |  |  |
| *Anaplasma phagocytophilum* HZ | 41.6 | 39.7 | 1.9 | 1.5 |
| *Ehrlichia canis* str. Jake | 29 | 23 | 6 | 1.3 |
| *Ehrlichia chaffeensis* str. Arkansas | 30.1 | 24.5 | 5.6 | 1.2 |
| *Ehrlichia ruminantium* strain Welgevonden | 27.5 | 23.1 | 4.4 | 1.5 |
| *Neorickettsia sennetsu* strain Miyayama | 41.1 | 38.2 | 2.9 | 0.86 |
| *Orientia tsutsugamushi* str. Boryong | 30.5 | 25.3 | 5.2 | 2.1 |
| *Rickettsia akari* str. Hartford | 32.3 | 25.8 | 6.5 | 1.2 |
| *Rickettsia typhi* str. Wilmington | 28.9 | 20.4 | 8.5 | 1.1 |
| *Wolbachia pipientis* strain wPip | 34.2 | 26.8 | 7.4 | 1.5 |
| *Wolbachia* endosymbiont strain TRS of *Brugia malayi* | 34.2 | 28 | 6.2 | 1.1 |
|  |  |  | mean | ∆ GC STD |
|  |  |  | 5.46 | 1.982254609 |
| ***BETA*** |  |  |  |  |
|  |  |  |  |  |
| *Azoarcus* sp. BH72 | 67.9 | 64.5 | 3.4 | 4.4 |
| *Burkholderia mallei* ATCC 23344 | 68.5 | 61.8 | 6.7 | 5.8 |
| *Methylobacillus flagellatus* KT | 55.7 | 46.8 | 8.9 | 3 |
| *Neisseria meningitidis* MC58 | 51.5 | 41.4 | 10.1 | 2.3 |
| *Nitrosomonas europaea* ATCC 19718 | 50.7 | 42.8 | 7.9 | 2.8 |
| *Nitrosospira multiformis* ATCC 25196 | 53.9 | 46.3 | 7.6 | 3.3 |
| *Thiobacillus denitrificans* ATCC 25259 | 66.1 | 60.4 | 5.7 | 2.9 |
|  |  |  | mean | ∆ GC STD |
|  |  |  | 7.185714 | 2.194256572 |
|  |  |  |  |  |
| ***GAMA*** |  |  |  |  |
|  |  |  |  |  |
| *Acinetobacter baumannii* ATCC 17978 | 38.9 | 34.5 | 4.4 | 4.02 |
| *Aeromonas hydrophila* subsp. *hydrophila* ATCC 7966 | 61.5 | 52.5 | 9 | 4.7 |
| *Escherichia coli* K12 MG1655 | 50.8 | 43.1 | 7.7 | 4.6 |
| *Teredinibacter turnerae* T7901 | 50 | 45.3 | 4.7 | 5.2 |
| *Yersinia pestis* Antiqua | 47.7 | 42.1 | 5.6 | 4.9 |
| *Xanthomonas campestris* pv. *campestris* str. 8004 | 65 | 62.9 | 2.1 | 5.2 |
|  |  |  | mean | ∆ GC STD |
|  |  |  | 5.583333 | 2.468535328 |
|  |  |  |  |  |
| ***DELTA*** |  |  |  |  |
|  |  |  |  |  |
| *Desulfotalea psychrophila* LSv54 | 46.6 | 38 | 8.6 | 3.7 |
| *Desulfovibrio vulgaris* subsp. *vulgaris* DP4 | 63.2 | 60.3 | 2.9 | 3.7 |
| *Geobacter uraniireducens* Rf4 | 54.2 | 45.8 | 8.4 | 5.1 |
| *Myxococcus xanthus* DK 1622 | 68.9 | 66.6 | 2.3 | 9.1 |
| *Sorangium cellulosum* 'So ce 56' | 71.4 | 68.9 | 2.5 | 13 |
| *Syntrophobacter fumaroxidans* MPOB | 59.9 | 55.8 | 4.1 | 5 |
|  |  |  | mean | ∆ GC STD |
|  |  |  | 4.8 | 2.933939331 |
|  |  |  |  |  |
| ***EPSILON*** |  |  |  |  |
|  |  |  |  |  |
| *Arcobacter butzleri* RM4018 | 27 | 21.3 | 5.7 | 2.3 |
| *Campylobacter jejuni* RM1221 | 30.3 | 24.1 | 6.2 | 1.8 |
| *Helicobacter pylori* J99 | 39.2 | 31.4 | 7.8 | 1.6 |
| *Nitratiruptor* sp. SB155-2 | 39.7 | 34.7 | 5 | 1.9 |
| *Sulfurovum* sp. NBC37-1 | 43.9 | 33.8 | 10.1 | 2.6 |
|  |  |  | mean | ∆ GC STD |
|  |  |  | 6.96 | 2.035436071 |
|  |  |  |  |  |
| ***SPIROCHAETAE*** |  |  |  |  |
|  |  |  |  |  |
| *Borrelia afzelii* PKo | 27.8 | 24.4 | 3.4 | 1.2 |
| *Borrelia burgdorferi* B31 | 28.2 | 23.7 | 4.5 | 1.5 |
| *Brachyspira hyodysenteriae* WA1 | 27.1 | 18.9 | 8.2 | 3 |
| *Leptospira biflexa* serovar Patoc strain 'Patoc 1 (Ames)' | 38.9 | 35.4 | 3.5 | 4 |
| *Leptospira interrogans* serovar lai str. 56601 | 35 | 31.5 | 3.5 | 4.7 |
| *Treponema denticola* ATCC 35405 | 37.9 | 30.7 | 7.2 | 2.8 |
|  |  |  | mean | ∆ GC STD |
|  |  |  | 5.05 | 2.11541958 |
|  |  |  |  |  |
| ***THERMOTOGAE*** |  |  |  |  |
|  |  |  |  |  |
| *Petrotoga mobilis* SJ95 | 34.1 | 32.8 | 1.3 | 2.2 |
| *Thermosipho melanesiensis* BI429 | 31.4 | 32 | -0.6 | 1.9 |
| *Thermotoga maritima* MSB8 | 46.2 | 45 | 1.2 | 1.9 |
|  |  |  | mean | ∆ GC STD |
|  |  |  | 0.633333 | 1.069267662 |
|  |  |  |  |  |
| ***THERMUS DEINOCOCCUS*** |  |  |  |  |
|  |  |  |  |  |
| *Deinococcus geothermalis* DSM 11300 | 66.5 | 64.2 | 2.3 | 3.3 |
| *Thermus thermophilus* HB8 | 69.5 | 64.9 | 4.6 | 2.1 |
|  |  |  |  |  |
| ***BACTEROIDETES*** |  |  |  |  |
|  |  |  |  |  |
| ***BATEROIDACEAE*** |  |  |  |  |
|  |  |  |  |  |
| *Bacteroides fragilis* NCTC 9343 | 43.1 | 27.8 | 15.3 | 5.2 |
| Bacteroides fragilis *YCH46* | 43.2 | 28.4 | 14.8 | 5.3 |
| *Bacteroides thetaiotaomicron* VPI-5482 | 42.9 | 27.2 | 15.7 | 6.3 |
| *Bacteroides vulgatus* ATCC8482 | 42.2 | 26.8 | 15.4 | 5.2 |
| *Bacteroides cellulosilyticus* DSM 14838 | 42.7 | 29.2 | 13.5 | 6.7 |
| *Bacteroides caccae* ATCC 43185 | 41 | 27.9 | 13.1 | 4.6 |
| *Bacteroides coprocola* DSM 17136 | 41 | 30.3 | 10.7 | 4.3 |
| *Bacteroides ovatus* ATCC 8483 | 41 | 28.2 | 12.8 | 6.5 |
| *Bacteroides uniformis* ATCC 8492 | 46 | 31.4 | 14.6 | 4.7 |
|  |  |  |  |  |
| ***PREVOTELLACEAE*** |  |  |  |  |
|  |  |  |  |  |
| *Prevotella intermedia* 17 | 43.5 | 33.7 | 9.8 | 2.7 |
| *Prevotella ruminicola* 23 | 47.7 | 30 | 17.7 | 3.6 |
| *Prevotella copri* DSM 18205 | 44.9 | 28.4 | 16.5 | 3.3 |
| *Prevotella melaninogenica* ATCC 25845 | 41 | 28.2 | 12.8 | 3.2 |
|  |  |  |  |  |
| ***PORPHYROMONADACEAE*** |  |  |  |  |
|  |  |  |  |  |
| *Parabacteroides merdae* ATCC 43184 | 45.3 | 30.7 | 14.6 | 4.4 |
| *Parabacteroides distasonis* ATCC 8503 | 45.1 | 27.8 | 17.3 | 4.8 |
| *Parabacteroides johnsonii* DSM 18315 | 45 | 31.4 | 13.6 | 4.6 |
| *Porphyromonas gingivalis ATCC 33277* | 48.4 | 36.4 | 12 | 2.4 |
| *Porphyromonas gingivalis* W38 | 48.3 | 37.4 | 10.9 | 2.3 |
| *Porphyromonas uenonis* 60-3 | 52.3 | 40.9 | 11.4 | 2.2 |
| *Porphyromonas endodontalis* ATCC 35406 | 47 | 36.7 | 10.3 | 2.1 |
|  |  |  |  |  |
| ***RIKENELLACEAE*** |  |  |  |  |
|  |  |  |  |  |
| *Alistipes putredinis* DSM 17216 | 53.3 | 38.3 | 15 | 2.6 |
|  |  |  |  |  |
| ***FLAVOBACTERIACEAE*** |  |  |  |  |
|  |  |  |  |  |
| *Capnocytophaga ochracea* DSM 7271 | 39.5 | 26.5 | 13 | 2.6 |
| *Chryaseobacteriu gleum* ATCC 35910 | 36.8 | 24.1 | 12.7 | 5.6 |
| *Flavobacterium johnsoniae* UW101 | 34.1 | 22.5 | 11.6 | 6.1 |
| *Flavobacterium psychrophilum* JIP02/86 | 32.5 | 20.8 | 11.7 | 2.9 |
| *Gramella forsetii* KT0803 | 36.6 | 25.5 | 11.1 | 3.8 |
| *Kordia algicida* OT-1 | 34.3 | 25.1 | 9.2 | 5 |
| *Leeuwenhoekiella blandensis* MED217 | 39.8 | 29.1 | 10.7 | 4.2 |
| *Polaribacter irgensii* 23-P | 34 | 24.7 | 9.3 | 2.7 |
| *Robiginitalea biformata* HTCC2501 | 55 | 41.9 | 13.1 | 3.5 |
|  |  |  |  |  |
|  |  |  |  |  |
| ***SPHINGOBACTERIACEAE*** |  |  |  |  |
|  |  |  |  |  |
| *Sphingobacterium spiritivorum* ATCC 33861 | 39.8 | 27.5 | 12.3 | 5.1 |
| *Pedobacter heparinus* DSM 2366 | 42 | 29.4 | 12.6 | 5.1 |
|  |  |  |  |  |
| ***CYTOPHAGACEAE*** |  |  |  |  |
|  |  |  |  |  |
| *Algoriphagus* sp. PR1 | 38 | 30 | 8 | 4.8 |
| *Cytophaga hutchinsonii* ATCC 33406 | 38.8 | 28 | 10.8 | 4.4 |
| *Dyadobacter fermentans* DSM 18053 | 51.5 | 38.3 | 13.2 | 6.9 |
| *Microscilla marina* ATCC 23134 | 40 | 30.2 | 9.8 | 9.8 |
| *Spirosoma linguale* DSM 74 | 50 | 40.2 | 9.8 | 8.5 |
|  |  |  |  |  |
| ***CHITINOPHAGACEAE*** |  |  |  |  |
|  |  |  |  |  |
| *Chitinophaga pinensis* DSM 2588 | 45.2 | 33.3 | 11.9 | 9.1 |
|  |  |  | mean | ∆ GC STD |
|  |  |  | 12.59474 | 2.335269805 |
| ***RHODOTHERMACEAE*** |  |  |  |  |
|  |  |  |  |  |
| *Salinibacter ruber* DSM 13855 | 66.1 | 60.72 | 5.38 | 3.6 |
| *Rhodothermus marinus* DSM 4252 | 64.3 | 55.8 | 8.5 | 3.4 |
|  |  |  |  |  |
|  |  |  |  |  |
|  |  |  |  |  |
