# Supplementary material for: Inability of Prevotella bryantii to Form a Functional Shine-Dalgarno Interaction Reflects Unique Evolution of Ribosome Binding Sites in Bacteroidetes
Source: PLoS One. 2011 Aug 12;6(8):e22914. doi: 10.1371/journal.pone.0022914 (PMC3155529; doi:10.1371/journal.pone.0022914)
Supplement: Table S3 — Primers used in the study. (DOC) [file pone.0022914.s022.doc]

| name | sequence from 5` |
| --- | --- |
| nucB | GCCGCTGCAGTAACGAAATTTCAATAGGTAATTC |
| SD addition | GCCGCTGCAGTAACGAAATTTCAATAGGTAATTCTATATAAAGGAGGTTCACTTATGCAC |
| PINA_1201 | GCCGCTGCAGTAAGTAAAAAATACAAACATATAAATTAATAGGAGGAAAAGATT ATGCACACAATTAAATCTTTATTTTTAGCC |
| partial removal | GCCGCTGCAGTAAGTAAAAAATACAAACATATAAATTAATAGGACCAAAAGATT ATGCACACAATTAAATCTTTATTTTTAGCC |
| full removal | GCCGCTGCAGTAAGTAAAAAATACAAACATATAAATTAATTCCCCCAAAAGATTATGCACACAATTAAATCTTTATTTTTAGCC |
| SD6 | GCCGCTGCAGTAATGAGCGGATAACAATTTGGGATCCAAAGGAGAAAAACATG CACACAATTAAATCTTTATTTTTAGCC |
| SD8 | GCCGCTGCAGTAATGAGCGGATAACAATTTGGGATCCAAAGGAGGTAAAAACATG CACACAATTAAATCTTTATTTTTAGCC |
| SD10 | GCCGCTGCAGTAATGAGCGGATAACAATTTGGGATCCAAAGGAGGTGAAAAAACATGCACACAATTAAATCTTTATTTTTAGCC |
| pstnucBrhis | GCCGCTGCAGTTATTAATGATGATGATGATGATGAGAACCCCCGTTAAGTTTCCAGGCTCTTAG |
| 0 | GCCGCTGCAGTAAACAAAGAAAGTTAAAAATACAACGTTATAAATTAATTTAGGAGGAATATTATGCACACAATTAAATCTTTATTTTTAGCC |
| 11,1 | GCCGCTGCAGTAAACAAAGAAAGTTAAAAATACAACGTTATCCTCCTATTTAGGAGGAATATTATGCACACAATTAAATCTTTATTTTTAGCC |
| 5,2 | GCCGCTGCAGTAAACAAAGAAAGTTAAAAATACAACGTTAACCTCAAATTTAGGAGGAATATTATGCACACAATTAAATCTTTATTTTTAGCC |
| 2,1 | GCCGCTGCAGTAAACAAAGAAAGTTAAAAATACAACGTTAAACTCAAATTTAGGAGGAATATTATGCACACAATTAAATCTTTATTTTTAGCC |
| mg0 | GCCGCTGCAGCTAAACAAAGAAAGTTAAAAATACAACGTTATAAATTAATTTAGGAGGAATATTATGGGCGTGATCAAGCCCGAC |
| mg11,1 | GCCGCTGCAGCTAAACAAAGAAAGTTAAAAATACAACGTTATCCTCCTATTTAGGAGGAATATTATGGGCGTGATCAAGCCCGAC |
| mg5,2 | GCCGCTGCAGCTAAACAAAGAAAGTTAAAAATACAACGTTAACCTCAAATTTAGGAGGAATATTATGGGCGTGATCAAGCCCGAC |
| mg reverse pst his | GCCGCTGCAGTTATTAATGATGATGATGATGATGAGAACCCCCGCCGGCCTGGCGGGGTAG |
| -20 | GCCGCTGCAGTAAACAAAGAAAGTTAAAAATACAACGTTATAAGCGCGAAAACGCGCATTAATTTAGGAGGAATATTATGCACACAATTAAATCTTTATTTTTAGCC |
| mgrtF | CCTTCCCCGAGGGCTACTC |
| mgrtR | CAGATTCCCTGGTCCTCGTATG |
| ecort16SF | TGGCTTCCGGAGCTAACG |
| ecort16SR | TTTAACCTTGCGGCCGTACT |
| 11,1inv | GCCGCTGCAGTAAACAAAGAAAGTTAAAAATACAACGTTATAGGAGGATTTCCTCCTAATATTATGCACACAATTAAATCTTTATTTTTAGCC |
| 5,2inv | GCCGCTGCAGTAAACAAAGAAAGTTAAAAATACAACGTTAAGAGGAAATTTAGCCTCAATATTATGCACACAATTAAATCTTTATTTTTAGCC |
